# Iodophor-Catalyzed Disulfenylation of Amino Naphthalenes with Aryl Sulfonyl Hydrazines

**DOI:** 10.3390/molecules29112411

**Published:** 2024-05-21

**Authors:** Yutong Yuan, Jing He, Xiaowei Ma, Sheng Han, Yan Liu

**Affiliations:** State Key Laboratory Incubation Base for Green Processing of Chemical Engineering, School of Chemistry and Chemical Engineering, Shihezi University, Shihezi 832004, China; 18616149673@163.com (Y.Y.); hejing@shzu.edu.cn (J.H.); hansheng654321@sina.com (S.H.)

**Keywords:** C-H sulfenylation, water, sulfonyl hydrazine, TBAI (tetrabutylammonium iodide)

## Abstract

An iodophor-catalyzed direct disulfenylation of amino naphthalenes with aryl sulfonyl hydrazines in water was developed. A series of aryl sulfides were obtained in moderate to excellent yields. The advantages of this green protocol were the simple reaction conditions (metal-free, water as the solvent, under air), the odorless and easily available sulfur reagent, the broad substrate scope, and gram-scale synthesis. Moreover, the potential application of aryl sulfides was exemplified by further transformations.

## 1. Introduction

**Scheme 1:**
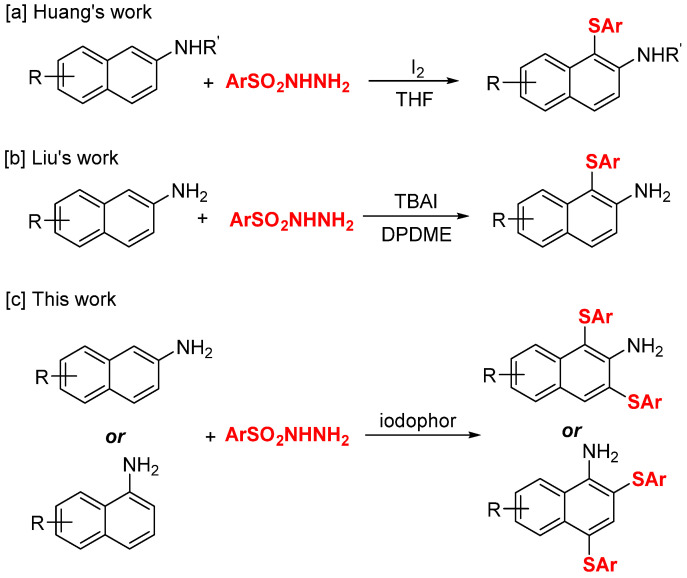
Sulfenylation of naphthalene amines. [**a**] Huang’s work; [**b**] Liu’s work; [**c**] This work.

As important organosulfides, diaryl sulfides have a wide range of applications in the fields of organic synthesis, medicinal chemistry, and materials science due to their unique biological activities and physical properties [1,2,3,4]. Therefore, the development of green and efficient strategies for the synthesis of diaryl sulfides is one of the hot spots in current research [5]. In recent years, the synthesis of diaryl sulfides by direct C-H sulfenylation of arenes has received much attention because of the shortening of the reaction steps and the reduction in waste generation [6,7]. In particular, the use of water as a safe, cheap, and environmentally friendly solvent makes sulfenylation greener and more economical. Examples of relevant processes include the following: (i) copper-catalyzed three-component reaction of arenes, iodoaromatics (or iodoalkanes), and sulfur [8]; (ii) iodine-mediated C-H sulfenylation of arenes [9,10,11,12]; (iii) cobalt-catalyzed aerobic couplings of C-H and thiols [13]; (iv) potassium persulfate–glucose-mediated sulfenylation of indole and thiophenols [14]. In 2015, Kang et al. reported an iodine-mediated thiolation of substituted naphthylamines and aryl sulfonyl hydrazides via direct C-H bond functionalization (Scheme 1a) [15]. In 2020, Liu et al. developed the TBAI-promoted sulfenylation of 2-aminonaphthalene and aryl sulfonyl hydrazides in the presence of DPDME (dipropylene glycol dimethyl ether) as a green solvent (Scheme 1b) [16]. However, all of the above reactions result in mono-substituted aryl thioethers. Double-substituted aryl sulfides have not been realized as yet.

It is well known that iodophors are now widely used as bactericidal products for surface disinfection, wound treatment, pre- and post-operative care, mouthwashes, gargles, and nasal sprays [17]. Studies have shown that iodophor as a highly effective topical disinfectant is not significantly cytotoxic or irritating [18]. However, to the best of our knowledge, the use of iodophor as a catalyst for organic synthesis reactions has not been reported. Considering that povidone iodine as a commercially readily available reagent has the advantage of possessing a two-component reagent (iodine and polyvinylpyrrolidone surfactant), this provides a rare opportunity for iodine-catalyzed organic reactions in the aqueous phase. On the basis of our previous research work [19,20,21,22], we herein report the iodophor-catalyzed disulfenylation of naphthylamine with aryl sulfonyl hydrazines; a series of 1,3-bis(arylthio)naphthalene-2-amines and 2,4-bis(arylthio)naphthalene-1-amines were obtained in moderate to excellent yields (Scheme 1c).

## 2. Results and Discussion

Initially, 0.3 mmol of 2-aminonaphthalene **1a** and 0.6 mmol of phenylsulfonyl hydrazine **2a** were chosen for a model reaction to investigate the feasibility of the Sulfite reaction (Table 1). The results showed that the product 1,3-bis(phenylthio)naphthalene-2-amine **3a** was successfully obtained in the presence of 7 mL of iodophor at 100 °C for 24 h (Table 1, entry 1). The dosage study of iodophor showed that 2 mL was the most efficient in producing the corresponding product **3a** in a 60% yield (entries 2–5). When the ratio of **1a** to **2a** was 1:3, 2 mL of iodophor was still the most catalytically effective and the desired product was obtained in a 90% yield (entries 6–8). Changes in reaction temperature are detrimental to the reaction (entries 9–11). Shortening the reaction time resulted in a significant decrease in the reaction yield (entries 12). The yield of product **3a** declined to 71% when the ratio of **1a** to **2a** was adjusted to 1:4 (entries 13). Finally, the optimum reaction conditions were determined: **1a** (0.3 mmol), **2a** (0.9 mmol), and iodophor (2 mL) under air at 100 °C for 24 h.

Under optimal reaction conditions, we investigated the range of 1,3-bisulfenylation reactions of *β*-naphthylamine with aryl sulfonyl hydrazides, and the results are summarized in Table 2. Benzenesulfonyl hydrazide substrates with various substituents attached to the 4-position such as methyl, methoxy, tert-butyl, fluoro, chloro, bromo, trifluoromethyl, and trifluoromethoxy were able to react smoothly with 2-naphthylamine, and the 1,3-bisulfenylated products **3a**–**3h** were obtained in moderate yields. The electronic effect of the substituent group seems to have no influence on the reaction. Likewise, the meso-substituted aryl sulfonyl hydrazides showed similar reactivity to afford the products **3i** and **3j**. It is worth mentioning that substrates with some spatial site resistance such as 2-methyl-, 2-fluoro-, 2,4-dimethyl-, and 2,4-dichloro-substituted benzenesulfonyl hydrazides are also suitable reactants to provide the desired products **3k**–**3n** in good yields. Heterocyclic substrates such as 2-thiophenesulfonyl hydrazide were also successfully converted to 1,3-bis(thiophen-2-ylthio)-2-naphthyl amine product **3o** in a 55% yield. 6-Bromon-2-naphthyl-amine was used as a substrate to react successfully with phenylsulfonyl hydrazide to give the desired product **3p** in a 60% yield. Predictably, the presence of bromine substituent offered the possibility of further conversions of the product **3p**.

Subsequently, we investigated the 1,3-disulfenylation of 1-aminonaphthalene with aryl sulfonyl hydrazides (Table 3). The results showed that aryl sulfonyl hydrazides with all types of substituents reacted equally well, and a series of 2,4-diaryl sulfide products **4a**–**4j** **[12]** were obtained in good yields. However, for benzenesulfonyl hydrazides with strong electron-withdrawing groups (4-nitro, 3-bromo, and 3-trifluoromethyl) in the para- or meso-positions as reactants, only mono-substituted 4-sulfenylated products **4k**–**4m** **[12]** could be obtained, and we speculate that this may be mainly due to the electronic effect of the substituent groups.

**Scheme 2:**
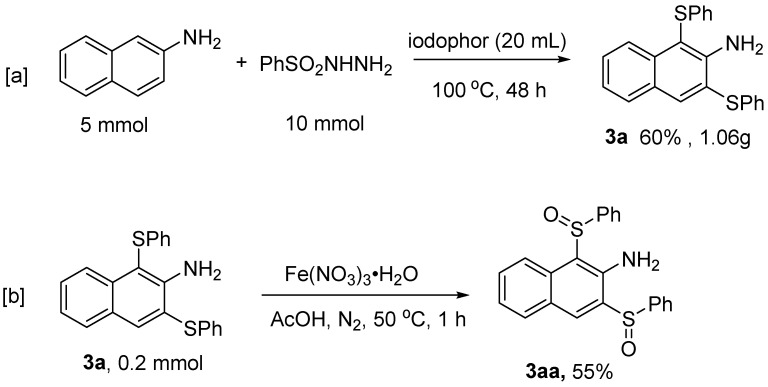
[**a**] Gram-scale synthesis and transformations of **3a**. [**b**] The oxidation reaction of **3a**.

To demonstrate the utility of this reaction, we performed a scale-up reaction based on optimal conditions (Scheme 2). The reaction of 5 mmol of 2-aminonaphthalene with phenylsulfonyl hydrazine (10 mmol) in the presence of 20 mL of iodophor afforded 1.06 g of **3a** product in a 60% yield (Scheme 2a). Subsequently, further derivatization of **3a** was explored. Under the condition of acetic acid as a solvent, ferric nitrate hydrate was able to oxidize **3a** to the corresponding sulfoxide product 1,3-bis(phenylsulfinyl)-2-naphthyl amine **3aa** in 55% yield (Scheme 2b).

**Scheme 3:**
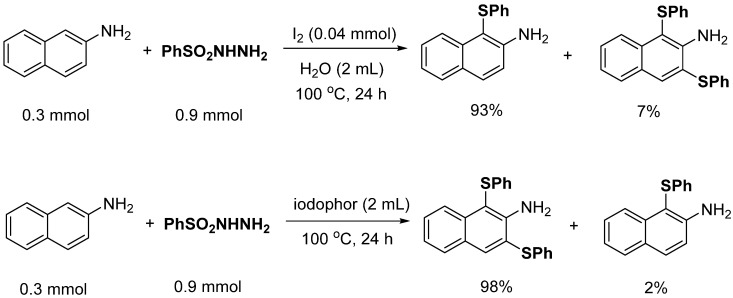
Control experiment of catalytic system.

In order to determine the innovation of the iodophor catalytic system and the selectivity of sulfinylation, related control experiments were designed and carried out. The controls used the same substrate concentration ratio, iodine dosage, temperature, and reaction time. According to the reaction (Scheme 3), a substituted 1-(phenylthio)-2-naphthyl-amine is mainly formed in the reaction system of iodine, while a disubstituted 1,3-bis(phenylthio)-2-naphthyl-amine is mainly formed in the reaction system of iodophor according to the reaction (Scheme 3). Therefore, it is speculated that iodophor provides a method to generate disulfide products.

**Scheme 4:**
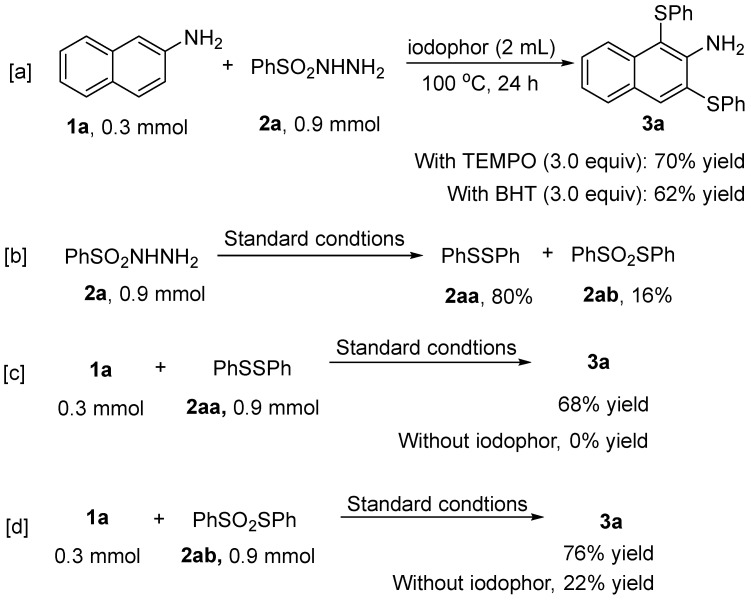
The control experiments. [**a**] Reactions in the presence of radical trapping reagents. [**b**] The reactivity of **2a** alone. [**c**] The reactivity of **2aa** without iodophor. [**d**] The reactivity of **2ab** without iodophor.

In order to probe the mechanism of this reaction, a series of control experiments were performed (Scheme 4). The reaction of 2-aminonaphthalene with phenylsulfonyl hydrazine occurred equally well without any significant decrease in the yield of the product 3a when the radical trapping agents TEMPO (2,2,6,6- tetramethylpiperidine oxide) or BHT (butylated hydroxytoluene) were added to the system of this reaction (Scheme 4a), which suggests that the disulfenylation reaction should not be a free radical-involved process. When benzenesulfonyl hydrazide was reacted under standard conditions alone, diphenyl disulfide **2aa** and S-phenyl benzenesulfonyl thioate **2ab** were obtained in 80% and 16% yields, respectively (Scheme 4b), which suggests that **2aa** and **2ab** may be important intermediates produced during the reaction. In order to prove this conjecture, the feasibility of 2-aminonaphthalene disulfenylation reaction was explored using **2aa** and **2ab** as sulfur sources, respectively. It was shown that the reaction of 2-aminonaphthalene with **2aa** had difficulty proceeding in the absence of iodophor (Scheme 4c), whereas the reaction of 2-aminonaphthalene with **2ab** was possible under the same conditions, and the presence of iodophor significantly increased the yield of the disulfenylation reaction (Scheme 4d). All these facts demonstrate the important role played by povidone iodine in catalyzing the disulfenylation reaction.

**Scheme 5:**
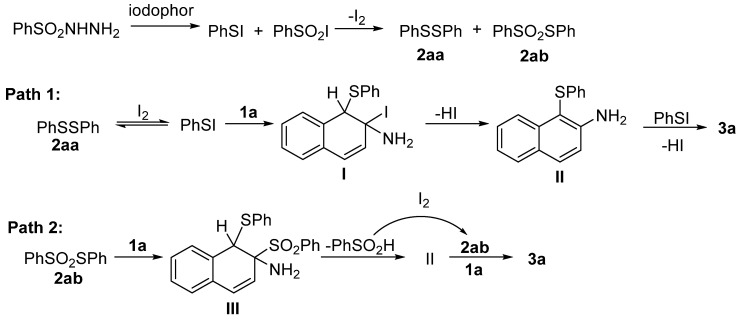
Proposed reaction mechanism.

Based on the above investigation and analysis, a plausible mechanism is outlined in Scheme 5. The reaction is initiated by benzenesulfonyl hydrazine, which can be transformed by I_2_ to form the intermediate **2aa** and **2ab**. Next, the sulfenylation of naphthylamine is mainly achieved through the following two routes: (1) Phenyl hypoiodothioite formed by the reaction of **2aa** with I_2_ undergoes an electrophilic addition reaction with β-naphthylamine to give intermediate I. This intermediate loses HI to provide intermediate II. Subsequently, intermediate II repeats the above reaction process to obtain product **3a** (Path 1). (2) The intermediate **2ab** as an electrophile reacts with **1a** to form III. The generated benzenesulfinic acid can be converted into **2ab** in the presence of I_2_. Finally, the intermediate II reacts with the generated **2ab** to obtain the product **3a** (Path 2).

## 3. Materials and Methods

Unless otherwise stated, all reactions were carried out experimentally in Schlenk tubes. Melting points were determined with a fusion meter and no other corrections were made. Materials obtained from commercial suppliers were used directly without further purification. Chromatography was performed using Qingdao Ocean Chemical 200–300 mesh silica gel. Spectra of ^1^H NMR, ^13^C NMR (in the Supplementary Materials) were recorded on a Bruker Avance III HD 400 MHz spectrometer in CDCl_3_ or DMSO-d_6_ solution, and chemical shifts (δ) relative to the internal standard tetramethylsilane (TMS) (0 ppm) were reported. High-resolution mass spectrometry (HRMS) was performed on a Thermo Scientific LTQ or a bitrap XL mass spectrometer, Thermo-fisher Q Exactive.

### 3.1. General Procedure for Iodophor-Catalyzed Disulfenylation of Amino Naphthalenes with Aryl Sulfonyl Hydrazines

A mixture of amino naphthalene (0.3 mmol), aryl sulfonyl hydrazine (0.9 mmol), and iodophor (2 mL) was added to a Schlenk tube. The solution was stirred continuously at 100 °C for 24 h. After completion of the reaction, the mixture was saturated with NaCl (10 mL) and extracted with CH_2_Cl_2_ (10 mL × 3). The combined CH_2_Cl_2_ extracts were dried with anhydrous Na_2_SO_4_, filtered, and concentrated under reduced pressure. The crude residue was purified by fast column chromatography on silica gel by using PE (petroleum ether)/EA (ethyl acetate) as eluents.

### 3.2. Gram Synthesis Procedure for ***3a***

A mixture of β-amino naphthalene (5 mmol), phenylsulfonyl hydrazine (10 mmol), and iodophor (20 mL) was added to a Schlenk tube. The solution was stirred continuously at 100 °C for 48 h. After completion of the reaction, the mixture was saturated with NaCl (20 mL) and extracted with CH_2_Cl_2_ (20 mL × 3). The combined CH_2_Cl_2_ extracts were dried with anhydrous Na_2_SO_4_, filtered, and concentrated under reduced pressure. The crude residue was purified by fast column chromatography on silica gel by using PE/EA as eluents (**3a**, 60% yield, 1.06 g).

### 3.3. Synthesis Procedure of ***3aa***

To a 10 mL reaction tube, we added **3a** (0.2 mmol), acetic acid (1 mL), ferric nitrate nonahydrate (0.5 equiv.), and iodophor (2 mL). The solution was stirred at 50 °C for 1 h. When the reaction was complete, it was neutralized with saturated sodium bicarbonate solution and extracted with ethyl acetate (20 mL). The solvent was removed under reduced pressure and the crude product was purified by fast chromatography on silica gel (PE/EA) to afford the final product 1,3-bis(phenylsulfinyl)-2-naphthyl-amine **3aa** in a 55% yield.

*1,3-bis(phenylthio)-2-naphthyl-amine* (**3a**): red oily liquid; ^1^H NMR (400 MHz, CDCl_3_) δ 8.19 (d, J = 8.5 Hz, 1H), 7.69–7.60 (m, 2H), 7.48 (d, J = 8.3 Hz, 3H), 7.32 (d, J = 7.4 Hz, 2H), 7.19–7.15 (m, 1H), 7.06 (t, J = 7.5 Hz, 2H), 7.00–6.88 (m, 4H), 4.61 (s, 2H). ^13^C NMR (101 MHz, CDCl_3_) δ 148.22, 142.63, 136.31, 133.41, 131.54, 131.18, 129.19, 128.70, 128.56, 128.12, 127.49, 127.27, 125.58, 124.76, 123.93, 122.29, 117.45, 104.25. HRMS (ESI): *m*/*z* calcd for C_22_H_18_NS_2_ (M+H)^+^: 360.0883, found: 360.0881.

*1,3-bis((4-methoxyphenyl)thio)-2-naphthyl-amine* (**3b**): red solid; mp = 68–71 °C; ^1^H NMR (400 MHz, CDCl_3_) δ 8.31 (s, 1H), 7.71 (d, J = 9.4 Hz, 3H), 7.47–7.42 (m, 2H), 7.03–7.00 (m, 4H), 6.73 (d, J = 9.1 Hz, 3H), 4.74 (s, 2H), 3.71 (d, J = 1.2 Hz, 6H). ^13^C NMR (101 MHz, CDCl_3_) δ 148.91, 136.48, 134.59, 132.64, 131.91, 130.97, 130.97, 129.43, 129.43, 128.66, 128.61, 128.29, 127.60, 126.88, 123.89, 123.02, 117.79, 102.51, 32.08, 29.86. HRMS (ESI): *m*/*z* calcd for C_24_H_21_NO_2_S_2_ (M+H)^+^: 420.1086, found: 420.1092.

*1,3-bis((4-fluorophenyl)thio)-2-naphthyl-amine* (**3c**): red oily liquid; ^1^H NMR (400 MHz, CDCl_3_) δ 8.14 (d, J = 8.4 Hz, 1H), 7.80 (d, J = 8.8 Hz, 1H), 7.74 (d, J = 8.0 Hz, 1H), 7.45 (t, J = 8.4 Hz, 3H), 7.39 (d, J = 3.3 Hz, 2H), 7.21 (d, J = 8.6 Hz, 1H), 7.07 (d, J = 8.8 Hz, 1H), 6.88 (d, J = 8.5 Hz, 1H), 6.33 (d, J = 8.6 Hz, 1H), 5.30 (s, 1H), 4.72 (s, 2H). ^13^C NMR (101 MHz, CDCl_3_) δ 134.17 (d, J = 87.4 Hz), 132.81 (d, J = 33.4 Hz). δ 134.60, 133.73, 132.97, 132.64, 129.78, 129.78, 129.43, 128.76, 128.66, 128.29, 128.12, 128.12, 127.60, 126.88, 123.89, 123.89, 123.01, 117.79. HRMS (APCI): *m*/*z* calcd for C_22_H_15_F_2_NS_2_ (M+H)^+^: 396.0690, found: 396.0686.

*1,3-bis((4-chlorophenyl)thio)-2-naphthyl-amine* (**3d**): red solid; mp = 80–83 °C; ^1^H NMR (400 MHz, CDCl_3_) δ 8.21 (d, J = 8.5 Hz, 1H), 7.77 (dd, J = 22.6, 8.4 Hz, 3H), 7.47–7.39 (m, 3H), 7.30 (d, J = 7.4 Hz, 2H), 7.23 (d, J = 7.9 Hz, 2H), 7.09 (d, J = 8.8 Hz, 1H), 7.02 (d, J = 7.8 Hz, 1H), 5.30 (s, 2H). ^13^C NMR (101 MHz, CDCl_3_) δ 138.57, 136.50, 135.81, 132.53, 132.53, 129.50, 129.50, 128.74, 128.66, 128.19, 124.00, 124.00, 123.00, 122.78, 121.97, 121.06, 117.81, 117.81. HRMS (APCI): *m*/*z* calcd for C_22_H_15_Cl_2_NS_2_ (M+H)^+^: 428.0100, found: 428.0095.

*1,3-bis((4-bromophenyl)thio)-2-naphthyl-amine* (**3e**): red oily liquid; ^1^H NMR (400 MHz, CDCl_3_) δ 8.35–8.23 (m, 1H), 7.78–7.73 (m, 1H), 7.61 (d, J = 7.7 Hz, 1H), 7.49–7.46 (m, 1H), 7.40 (d, J = 9.8 Hz, 2H), 7.33 (d, J = 7.6 Hz, 1H), 7.17 (s, 1H), 7.05 (t, J = 7.4 Hz, 2H), 6.93 (t, J = 6.6 Hz, 2H), 6.69 (d, J = 7.8 Hz, 1H), 4.28 (s, 2H). ^13^C NMR (101 MHz, CDCl_3_) δ 137.16, 131.93, 129.19, 129.19, 129.09, 129.09, 129.04, 128.48, 127.90, 127.64, 127.64, 127.28, 127.28, 125.96, 125.15, 124.36, 122.71, 117.76. HRMS (APCI): *m*/*z* calcd for C_22_H_15_Br_2_NS_2_ (M+H)^+^: 515.9090, found: 515.9085.

*1,3-bis((4-(trifluoromethyl)phenyl)thio)-2-naphthyl-amin* (**3f**): red solid; mp = 85–87 °C; ^1^H NMR (400 MHz, CDCl_3_) δ 8.17 (s, 1H), 7.86–7.65 (m, 3H), 7.49–7.36 (m, 3H), 7.30 (d, J = 7.7 Hz, 3H), 7.07 (d, J = 8.6 Hz, 3H), 4.73 (s, 2H). ^13^C NMR (101 MHz, CDCl_3_), δ 128.62 (d, J = 8.4 Hz), δ 148.78, 142.18, 136.51, 132.54, 132.54, 128.67, 128.58, 128.23, 125.94, 125.94, 125.66, 125.66, 125.66, 123.92, 122.97, 122.97, 117.77, 117.77, 102.97, 98.23. HRMS (APCI): *m*/*z* calcd for C_24_H_15_F_6_NS_2_ (M+H)^+^: 494.0477, found: 494.0550.

*1,3-bis((4-(tert-butyl)phenyl)thio)-2-naphthyl-amine* (**3g**): red solid; mp = 103 -106 °C; ^1^H NMR (400 MHz, CDCl_3_) δ 8.31 (d, J = 8.5 Hz, 1H), 7.76–7.71 (m, 2H), 7.55–7.33 (m, 3H), 7.19 (d, J = 8.4 Hz, 3H), 7.01 (dd, J = 38.8, 8.6 Hz, 4H), 4.71 (s, 2H), 1.43–1.10 (m, 18H). ^13^C NMR (101 MHz, CDCl_3_) δ 148.39, 148.10, 136.75, 136.33, 133.23, 131.67, 131.67, 128.40, 128.31, 127.70, 127.46, 126.05, 126.05, 125.61, 124.38, 122.53, 117.63, 105.11, 34.31, 31.28, 31.28, 31.16. HRMS (APCI): *m*/*z* calcd for C_30_H_33_NS_2_ (M+H)^+^: 472.2132, found: 471.2127.

*1,3-bis(p-tolylthio)-2-naphthyl-amine* (**3h**): red oily liquid; ^1^H NMR (400 MHz, CDCl_3_) δ 8.28 (s, 1H), 7.73 (d, J = 3.8 Hz, 1H), 7.47 (d, J = 8.0 Hz, 2H), 7.25–7.20 (m, 4H), 7.15 (d, J = 7.8 Hz, 2H), 6.99–6.94 (m, 3H), 4.59 (s, 2H), 2.40 (d, J = 16.4 Hz, 6H). ^13^C NMR (101 MHz, CDCl_3_) δ 148.46, 144.70, 144.70, 142.16, 142.16, 140.57, 136.61, 133.30, 131.74, 130.32, 129.86, 127.81, 127.71, 126.17, 124.40, 122.62, 117.77, 105.31, 21.78, 20.99. HRMS (APCI): *m*/*z* calcd for C_24_H_21_NS_2_ (M+H)^+^: 388.1122, found: 388.1188.

*1,3-bis((3-bromophenyl)thio)-2-naphthyl-amine* (**3i**): brown liquid; ^1^H NMR (400 MHz, CDCl_3_) δ 8.18 (s, 1H), 7.65 (s, 1H), 7.48 (d, J = 8.6 Hz, 2H), 7.33 (d, J = 11.7 Hz, 2H), 7.23 (s, 1H), 7.17 (s, 1H), 7.05 (d, J = 7.6 Hz, 2H), 6.95 (t, J = 8.1 Hz, 3H), 4.38 (s, 2H). ^13^C NMR (101 MHz, CDCl_3_) δ 148.51, 136.68, 136.68, 133.73, 131.90, 131.90, 129.53, 129.06, 129.06, 128.90, 128.46, 127.86, 127.64, 125.93, 125.93, 125.12, 124.31, 122.68, 122.68, 117.78, 117.78, 104.71. HRMS (APCI): *m*/*z* calcd for C_22_H_15_Br_2_NS_2_ (M+Na)^+^: 537.8915, found: 537.8910.

*1,3-bis((3-(trifluoromethyl)phenyl)thio)-2-naphthyl-amine* (**3j**): red solid; mp = 88–91 °C; ^1^H NMR (400 MHz, CDCl_3_) δ 8.20 (d, J = 8.5 Hz, 1H), 7.77 (dd, J = 22.3, 8.4 Hz, 3H), 7.47–7.40 (m, 2H), 7.32–7.28 (m, 2H), 7.22 (t, J = 7.8 Hz, 2H), 7.04 (dd, J = 22.4, 8.4 Hz, 3H), 4.73 (s, 2H).^13^C NMR (101 MHz, CDCl_3_), δ 128.55 (d, J = 103.5 Hz)**,** δ 137.42 (d, J = 208.3 Hz), δ 148.64, 138.46, 136.39, 132.37, 132.37, 131.47, 130.49, 129.35, 129.35, 128.57, 128.51, 128.51, 128.03, 125.96, 123.82, 123.82, 122.77, 122.61, 121.76, 117.60, 103.07, 103.07. HRMS (APCI): *m*/*z* calcd for C_24_H_15_F_6_NS_2_ (M-H)^+^: 494.0464, found: 494.0477.

*1,3-bis(o-tolylthio)-2-naphthyl-amine* (**3k**): red solid; mp = 101–103 °C; ^1^H NMR (400 MHz, CDCl_3_) δ 8.21 (d, J = 8.5 Hz, 1H), 7.76 (dd, J = 17.6, 8.3 Hz, 3H), 7.43 (t, J = 7.6 Hz, 2H), 7.18 (d, J = 7.4 Hz, 1H), 7.09 (d, J = 8.8 Hz, 2H), 6.99 (t, J = 7.0 Hz, 2H), 6.87 (d, J = 7.5 Hz, 1H), 6.44 (d, J = 7.9 Hz, 1H), 4.41 (s, 2H), 2.41 (d, J = 128.3 Hz, 6H). ^13^C NMR (101 MHz, CDCl_3_) δ 148.50, 136.82, 135.67, 135.01, 131.89, 130.23, 129.90, 128.68, 128.50, 128.50, 127.90, 127.90, 126.65, 126.22, 124.78, 124.78, 124.40, 124.30, 122.79, 122.79, 117.83, 104.34, 20.18, 20.18. HRMS (ESI): *m*/*z* calcd for C_24_H_21_NS_2_ (M+H)^+^: 388.1187, found: 388.1194.

*1,3-bis((2-fluorophenyl)thio)-2-naphthyl-amine* (**3l**): red liquid; ^1^H NMR (400 MHz, CDCl_3_) δ 8.16 (s, 1H), 7.82–7.68 (m, 3H), 7.55–7.42 (m, 2H), 7.39 (d, J = 7.9 Hz, 3H), 7.29 (s, 1H), 7.11–7.02 (m, 3H), 4.72 (s, 2H). ^13^C NMR (101 MHz, CDCl_3_), 134.94 (d, J = 103.9 Hz), 126.91 (d, J = 61.8 Hz), 124.96 (d, J = 57.1 Hz) 118.64 (d, J = 35.2 Hz), δ 143.86, 141.85, 138.04, 135.46, 134.43, 132.09, 131.66, 130.70, 128.40, 128.37, 127.93, 127.21, 126.60, 126.14, 125.66, 125.25, 124.68, 121.25, 120.59, 118.82, 118.47, 109.51. HRMS (ESI): *m*/*z* calcd for C_22_H_15_F_2_NS_2_ (M-H)^+^: 395.0614, found: 395.0614.

*1,3-bis((2,4-dimethylphenyl)thio)-2-naphthyl-amine* (**3m**): brown liquid; ^1^H NMR (400 MHz, CDCl_3_) δ 8.22 (d, J = 8.5 Hz, 1H), 7.76 (dd, J = 17.1, 8.4 Hz, 3H), 7.43 (t, J = 7.6 Hz, 2H), 7.07 (dd, J = 11.9, 8.2 Hz, 3H), 6.80 (d, J = 7.4 Hz, 2H), 6.27 (s, 2H), 2.52 (s, 6H), 2.01 (s, 6H). ^13^C NMR (101 MHz, CDCl_3_) δ 148.16, 138.54, 136.58, 135.94, 134.92, 134.11, 132.48, 132.28, 131.71, 131.48, 130.51, 130.39, 129.83, 128.36, 128.13, 127.50, 125.43, 124.47, 124.17, 122.40, 117.50, 104.23, 20.93, 20.26, 19.92, 19.41. HRMS (ESI): *m*/*z* calcd for C_22_H_13_Cl_4_NS_2_ (M+Na)^+^: 438.1317, found: 438.1326.

*1,3-bis((2,4-dichlorophenyl)thio)-2-naphthyl-amine* (**3n**): red oily liquid; ^1^H NMR (400 MHz, CDCl_3_) δ 8.21 (d, J = 8.6 Hz, 1H), 7.75 (dd, J = 20.4, 8.4 Hz, 3H), 7.45 (s, 1H), 7.18 (s, 2H), 7.08–6.98 (m, 3H), 6.88 (d, J = 8.0 Hz, 1H), 5.35 (s, 2H). ^13^C NMR (101 MHz, CDCl_3_) δ 143.51, 143.09, 136.73, 133.75, 133.75, 131.54, 129.57, 129.21, 129.19, 129.11, 129.11, 128.93, 127.70, 127.70, 127.64, 127.10, 126.47, 125.52, 125.45, 121.66, 118.71, 108.21. HRMS (ESI): *m*/*z* calcd for C_22_H_13_Cl_4_NS_2_ (M+H)^+^: 495.9310, found: 495.9322.

*1,3-bis(thiophen-2-ylthio)-2-naphthyl-amine* (**3o**): red oily liquid; ^1^H NMR (400 MHz, CDCl_3_) δ 7.71 (dd, J = 15.2, 8.4 Hz, 3H), 7.62 (d, J = 8.2 Hz, 1H), 7.40 (t, J = 7.5 Hz, 2H), 7.26 (t, J = 7.4 Hz, 2H), 7.01–6.95 (m, 3H), 3.77 (s, 2H). ^13^C NMR (101 MHz, CDCl_3_) δ 144.21, 135.02, 129.31, 129.31, 128.08, 128.08, 127.83, 127.83, 126.45, 126.45, 125.91, 125.91, 122.58, 122.58, 118.34, 118.34, 108.70, 108.70. HRMS (ESI): *m*/*z* calcd for C_18_H_13_NS_2_ (M+H)^+^: 371.9998, found: 372.0009.

*6-bromo-1,3-bis(phenylthio)-2-naphthyl-amine* (**3p**): red liquid; ^1^H NMR (400 MHz, CDCl_3_) δ 8.14 (d, J = 9.0 Hz, 1H), 7.85 (s, 1H), 7.66 (d, J = 8.8 Hz, 1H), 7.57 (d, J = 7.0 Hz, 1H), 7.49–7.46 (m, 1H), 7.44–7.39 (m, 1H), 7.34 (d, J = 6.9 Hz, 2H), 7.17 (t, J = 7.6 Hz, 2H), 7.08 (t, J = 7.9 Hz, 2H), 6.99 (d, J = 7.7 Hz, 2H), 4.79 (s, 2H). ^13^C NMR (101 MHz, CDCl_3_) δ 148.82, 136.74, 136.49, 135.46, 133.75, 131.54, 130.99, 130.90, 130.34, 129.57, 129.19, 129.19, 128.93, 127.71, 126.38, 125.98, 125.98, 125.39, 125.39, 118.79, 116.29, 104.86. HRMS (APCI): *m*/*z* calcd for C_22_H_16_BrNS_2_ (M+H)^+^ 437.9980, found: 437.9981.

*2,4-bis(phenylthio)naphthalen-1-amine* (**4a**) [16]: white solid; mp 100–102 °C; ^1^H NMR (400 MHz, CDCl_3_) δ 8.38 (d, J = 9.3 Hz, 1H), 7.97 (s, 1H), 7.79 (d, J = 7.8 Hz, 1H), 7.54–7.42 (m, 2H), 7.18 (t, J = 7.5 Hz, 2H), 7.1–7.06 (m, 5H), 7.03 (t, J = 7.5 Hz, 3H), 5.20 (s, 2H); ^13^C NMR (101 MHz, CDCl_3_) δ 147.85, 143.47, 139.24, 136.60, 136.07, 129.19, 128.91, 128.31, 127.20, 126.62, 126.49, 125.99, 125.73, 125.11, 123.89, 122.03, 117.26, 108.14, 77.16

*2,4-bis(p-tolylthio)naphthalen-1-amine* (**4b**) [16]: white solid; mp 101–103 °C; ^1^H NMR (400 MHz, CDCl_3_) δ 8.38 (d, J = 9.0 Hz, 1H), 7.93 (s, 1H), 7.76 (d, J = 8.0 Hz, 1H), 7.49 (t, J = 6.8 Hz, 1H), 7.43 (t, J = 7.5 Hz, 1H), 7.05–6.97 (m, 4H), 6.95 (t, J = 6.5 Hz, 4H), 5.14 (s, 2H), 2.24 (s, 3H), 2.21 (s, 3H); ^13^C NMR (101 MHz, CDCl_3_) δ 147.29, 142.79, 135.80, 135.72, 135.41, 135.03, 132.91, 129.95, 129.70, 128.06, 127.16, 127.04, 125.86, 123.92, 121.97, 118.03, 109.02, 77.16, 21.03, 20.99;

*2,4-bis((4-chlorophenyl)thio)naphthalen-1-amine* (**4c**) [16]: white solid; mp 97–99 °C; ^1^H NMR (400 MHz, CDCl_3_) δ 8.32 (d, J = 8.7 Hz, 1H), 7.92 (s, 1H), 7.81 (d, J = 8.9 Hz, 1H), 7.58–7.47 (m, 2H), 7.15 (d, J = 8.6 Hz, 2H), 7.09 (d, J = 8.6 Hz, 2H), 7.00 (d, J = 8.7 Hz, 2H), 6.93 (d, J = 8.6 Hz, 2H), 5.24 (s, 2H); ^13^C NMR (101 MHz, CDCl_3_) δ 148.03, 143.23, 137.74, 135.91, 135.08, 131.69, 130.99, 129.31, 129.02, 128.62, 127.88, 127.75, 127.03, 126.23, 123.86, 122.11, 116.99, 107.63, 77.16;

*2,4-bis((4-bromophenyl)thio)naphthalen-1-amine* (**4d**): white solid; mp 115–118 °C; ^1^H NMR (400 MHz, CDCl_3_) δ 8.32 (s, 1H), 7.93 (s, 1H), 7.84 (d, J = 10.8 Hz, 2H), 7.59–7.49 (m, 3H), 7.33 (d, J = 8.5 Hz, 2H), 6.92 (dd, J = 29.1, 8.6 Hz, 4H), 5.26 (s, 2H). ^13^C NMR (101 MHz, CDCl_3_) δ 143.65, 133.85, 132.57, 132.41, 132.27, 129.13, 129.03, 128.48, 128.36, 128.28, 127.63, 127.63, 126.62, 125.99, 122.47, 121.97, 119.21, 107.82. HRMS (APCI): *m*/*z* calcd for C_22_H_15_Br_2_NS_2_ (M-H)^+^ 513.8941, found: 513.8939.

*2,4-bis((4-fluorophenyl)thio)naphthalen-1-amine* (**4e**): white solid; mp 103–105 °C; ^1^H NMR (400 MHz, CDCl_3_) δ 8.37 (d, J = 7.3 Hz, 1H), 7.91 (s, 1H), 7.85 (d, J = 7.2 Hz, 1H), 7.58–7.51 (m, 2H), 7.10 (dd, J = 8.6, 5.3 Hz, 2H), 7.04 (dd, J = 8.6, 5.3 Hz, 2H), 6.90 (dt, J = 25.3, 8.6 Hz, 4H), 5.25 (s, 2H). ^13^C NMR (101 MHz, CDCl_3_), 126.31 (d, J = 90.0 Hz), 122.72 (d, J = 189.1 Hz), δ 147.21, 142.32, 135.43, 128.66, 128.58, 128.50, 128.10, 126.76, 125.86, 123.66, 121.78, 117.90, 116.16, 116.16, 115.94, 115.87, 115.66, 108.55. HRMS (APCI): *m*/*z* calcd for C_22_H_15_F_2_NS_2_ (M-H)^+^ 394.0545, found: 394.0541.

*2,4-bis((4-methoxyphenyl)thio)naphthalen-1-amine* (**4f**): red solid; mp 75–78 °C; ^1^H NMR (400 MHz, CDCl_3_) δ 8.41 (s, 1H), 7.82 (d, J = 6.8 Hz, 2H), 7.52 (d, J = 8.5 Hz, 2H), 7.11 (dd, J = 13.9, 8.8 Hz, 4H), 6.76 (dd, J = 18.7, 8.8 Hz, 4H), 5.35 (s, 2H), 3.75 (d, J = 9.3 Hz, 6H). ^13^C NMR (101 MHz, CDCl_3_) δ 158.58, 158.23, 141.17, 129.90, 129.90, 129.83, 129.01, 127.85, 127.84, 127.04, 125.91, 124.05, 121.95, 121.95, 121.95, 114.98, 114.98, 114.77, 55.50, 55.46. HRMS (APCI): *m*/*z* calcd for C_24_H_21_NO_2_S_2_ (M+H)^+^ 420.1088, found: 420.1086.

*2,4-bis((4-(tert-butyl)phenyl)thio)naphthalen-1-amine* (**4g**): white solid; mp 103–106 °C ^1^H NMR (400 MHz, CDCl_3_) δ 8.45 (d, J = 8.9 Hz, 1H), 7.97 (s, 1H), 7.86 (d, J = 7.4 Hz, 1H), 7.56–7.50 (m, 2H), 7.25 (d, J = 9.9 Hz, 2H), 7.18 (d, J = 8.4 Hz, 2H), 7.06 (d, J = 8.4 Hz, 2H), 6.99 (d, J = 8.4 Hz, 2H), 5.24 (s, 2H), 1.27 (s, 9H), 1.24 (s, 9H). ^13^C NMR (101 MHz, CDCl_3_) δ 148.85, 147.47, 143.19, 143.19, 135.55, 135.55, 132.99, 132.99, 128.04, 127.22, 126.52, 126.38, 126.15, 125.86, 125.80, 125.80, 123.81, 121.88, 117.74, 108.71, 34.40, 31.28. HRMS (APCI): *m*/*z* calcd for C_30_H_33_NS_2_ (M-H)^+^ 470.1983, found: 470.1981.

*2,4-bis(o-tolylthio)naphthalen-1-amine* (**4h**): red liquid; ^1^H NMR (400 MHz, CDCl_3_) δ 8.33 (s, 1H), 7.88–7.81 (m, 3H), 7.50 (dd, J = 26.8, 8.5 Hz, 4H), 7.18 (d, J = 7.6 Hz, 2H), 7.05–6.97 (m, 3H), 5.20 (s, 2H), 2.47 (d, J = 10.9 Hz, 6H). ^13^C NMR (101 MHz, CDCl_3_) δ 147.29, 142.62, 137.81, 133.34, 130.12, 129.76, 127.90, 126.83, 126.40, 126.14, 125.65, 125.25, 124.93, 124.64, 121.70, 118.81, 118.56, 117.09, 109.52, 107.78, 19.90, 19.90. HRMS (APCI): *m*/*z* calcd for C_24_H_21_NS_2_ (M-H)^+^ 388.1046, found: 386.1042.

*2,4-bis((2-fluorophenyl)thio)naphthalen-1-amine]* (**4i**): red solid; mp 75–78 °C; ^1^H NMR (400 MHz, CDCl_3_) δ 8.38 (s, 1H), 7.97 (s, 1H), 7.55 (d, J = 9.0 Hz, 2H), 7.10–7.01 (m, 4H), 6.94 (d, J = 8.0 Hz, 2H), 6.84 (d, J = 8.3 Hz, 2H), 6.65 (d, J = 7.9 Hz, 2H), 5.35 (s, 2H). ^13^C NMR (101 MHz, CDCl_3_), 132.64 (d, J = 222.7 Hz), 127.84 (d, J = 28.6 Hz), δ 136.73, 136.73, 133.75, 131.54, 129.57, 129.57, 129.22, 129.11, 128.93, 127.98, 127.70, 127.10, 126.66, 126.66, 126.52, 126.47, 126.47, 125.52, 125.45, 121.66, 118.71, 118.71.HRMS (APCI): *m*/*z* calcd for C_22_H_15_F_2_NS_2_ (M-H)^+^ 394.0544, found:395.0541.

*2,4-bis(thiophen-2-ylthio)naphthalen-1-amine* (**4j**): red liquid; ^1^H NMR (400 MHz, CDCl_3_) δ 7.67 (d, J = 8.6 Hz, 1H), 7.36 (d, J = 7.6 Hz, 1H), 7.27 (d, J = 8.5 Hz, 1H), 7.12–7.09 (m, 2H), 7.03 (d, J = 5.0 Hz, 2H), 6.93 (dq, J = 15.2, 7.8 Hz, 4H), 6.77–6.67 (m, 2H), 2.04–1.98 (m, 12H). ^13^C NMR (101 MHz, CDCl_3_) δ 141.18, 140.71, 138.85, 136.52, 135.85, 134.76, 134.42, 133.49, 132.80, 132.60, 132.57, 130.82, 130.70, 130.25, 128.74, 126.94, 126.90, 126.47, 126.34, 125.48, 121.66, 118.94, 20.65, 20.57, 20.22, 20.11. HRMS (APCI): *m*/*z* calcd for C_26_H_25_NS_2_ (M-H)^+^ 415.1430, found:415.1428.

*4-((3-bromophenyl)thio)naphthalen-1-amine* (**4k**): red liquid; ^1^H NMR (400 MHz, CDCl_3_) δ 8.32 (s, 1H), 7.84 (s, 1H), 7.71 (s, 1H), 7.51 (d, J = 4.1 Hz, 2H), 7.15 (d, J = 7.7 Hz, 2H), 6.98 (s, 1H), 6.89 (s, 1H), 6.80 (s, 1H), 4.43 (s, 2H). ^13^C NMR (101 MHz, CDCl_3_) δ 145.03, 142.53, 137.24, 135.52, 130.15, 128.56, 127.90, 127.57, 126.83, 125.59, 124.65, 124.42, 123.00, 121.42, 116.10, 109.53. HRMS (APCI): *m*/*z* calcd for C_16_H_16_BrNS (M+H)^+^ 329.9948, found:329.9946.

*4-((3-(trifluoromethyl)phenyl)thio)naphthalen-1-amin* (**4l**): red liquid; ^1^H NMR (400 MHz, CDCl_3_) δ 8.25 (s, 1H), 7.79 (s, 1H), 7.64 (s, 1H), 7.44 (d, J = 9.6 Hz, 2H), 7.21 (s, 2H), 7.13 (s, 1H), 6.97 (s, 1H), 6.75 (s, 1H), 4.38 (s, 2H). ^13^C NMR (101 MHz, CDCl_3_), δ 143.07 (d, J = 352.6 Hz), 136.07 (d, J = 180.5 Hz), δ 144.83, 141.32, 136.97, 135.18, 132.11, 128.88, 128.73, 127.31, 126.40, 125.31, 124.11, 122.32, 121.16, 119.11, 115.44, 109.22, 109.22. HRMS (APCI): *m*/*z* calcd for C_16_H_16_BrNS (M+H)^+^ 320.0711,found:320.0715.

*4-((4-nitrophenyl)thio)naphthalen-1-amin* (**4m**) [16]: yellow liquid; ^1^H NMR (400 MHz, CDCl_3_) δ 8.03 (d, J = 8.9 Hz, 2H), 7.90–7.78 (m, 2H), 7.63–7.49 (m, 2H), 7.45 (d, J = 8.5 Hz, 1H), 7.31 (d, J = 8.5 Hz, 1H), 7.11 (d, J = 8.9 Hz, 2H), 5.01 (s, 2H); ^13^C NMR (101 MHz, CDCl_3_) δ 147.40, 146.44, 145.38, 135.56, 133.18, 128.88, 127.76, 125.91, 125.61, 124.21, 123.16, 121.67, 119.22, 105.00, 77.16.

*1,3-bis(phenylsulfinyl)-2-naphthyl-amine* (**3aa**): red liquid; ^1^H NMR (400 MHz, CDCl_3_) δ 8.20 (d, J = 8.5 Hz, 1H), 7.67–7.62 (m, 2H), 7.50–7.44 (m, 1H), 7.37–7.30 (m, 2H), 7.17 (t, J = 7.4 Hz, 2H), 7.05 (d, J = 7.3 Hz, 2H), 6.96 (dd, J = 14.3, 7.5 Hz, 5H), 4.57 (s, 2H). ^13^C NMR (101 MHz, CDCl_3_) δ 148.27, 136.59, 136.37, 136.37, 131.58, 129.21, 128.75, 128.58, 128.16, 128.14, 127.55, 127.33, 125.61, 124.80, 123.99, 122.35, 117.43, 104.30. HRMS (APCI): *m*/*z* calcd for C_22_H_17_NO_2_S_2_ (M-H)^+^ 390.0631, found: 390.0627.

## 4. Conclusions

In summary, we developed the iodophor-catalyzed direct disulfenylation of amino naphthalenes with aryl sulfonyl hydrazines. This reaction was able to proceed smoothly under air in the aqueous phase without any additional phase-transfer reagents, and a series of novel aryl sulfides were obtained in moderate to excellent yields. The advantages of this green approach are the use of odorless and readily available sulfur reagents, a wide range of substrates, and gram-scale synthesis.

## Figures and Tables

**Table 1:** Optimization of the reaction conditions ^a^.

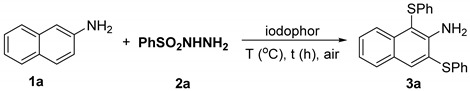					
Entry	1a:2a	Iodophor (mL)/ I_2_ (mmol)	T (°C)	t (h)	Yield (%) ^b^
1	1:2	7/0.14	100	24	55
2	1:2	3/0.06	100	24	58
3	1:2	2/0.04	100	24	60
4	1:2	1/0.02	100	24	57
5	1:2	0.5/0.01	100	24	43
6	1:3	3/0.06	100	24	68
7	1:3	2/0.04	100	24	90
8	1:3	1/0.02	100	24	65
9	1:3	2/0.04	120	24	46
10	1:3	2/0.04	90	24	65
11	1:3	2/0.04	100	12	63
12	1:4	2/0.04	100	24	71

^a^ Reaction conditions: **1a** (0.3 mmol), **2** (x mmol), iodophor (x mL), under air, 100 °C, 24 h. ^b^ Isolated yield.

**Table 2:** Iodophor-catalyzed disulfenylation of *β*-naphthylamine with ArSO_2_NHNH_2_^a^.

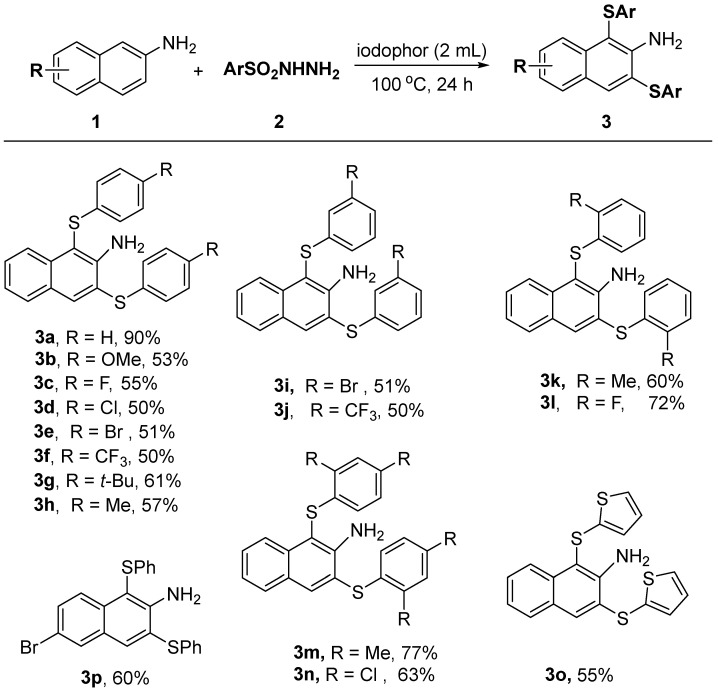

^a^ Reaction conditions: **1** (0.3 mmol), **2** (0.9 mmol), iodophor (2 mL), under air, 100 °C, 24 h.

**Table 3:** Iodophor-catalyzed disulfenylation of *α*-naphthylamine with ArSO_2_NHNH_2_^a^.

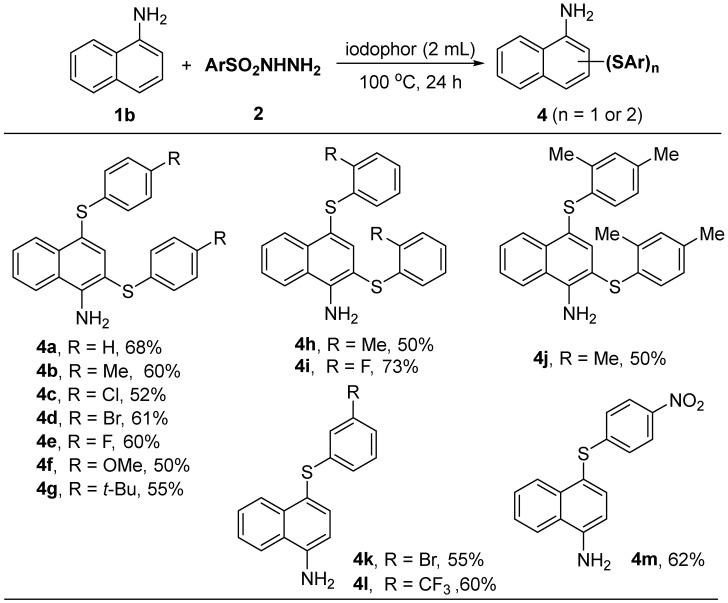

^a^ Reaction conditions: **1b** (0.3 mmol), **2** (0.9 mmol), iodophor (2 mL), under air, 100 °C, 24 h.

## Data Availability

Data are contained within the article.

## Supplementary Materials

The following supporting information can be downloaded at https://www.mdpi.com/article/10.3390/molecules29112411/s1: the charts of ^1^H-, ^13^C-NMR, and HRMS of products.

## References

[1] Mansy S.S., Cowan J.A. (2004). Iron-sulfur cluster biosynthesis: Toward an understanding of cellular machinery and molecular mechanism. Acc. Chem. Res..

[2] Block E. (2013). Fifty years of smelling sulfur. J. Sulfur Chem..

[3] Kochanowska-Karamyan A.J., Hamann M.T. (2010). Marine indole alkaloids: Potential new drug leads for the control of depression and anxiety. Chem. Rev..

[4] Gupta L., Talwar A., Chauhan P.M. (2007). Bis and tris indole alkaloids from marine organisms: New leads for drug discovery. Curr. Med. Chem..

[5] Kondo T., Mitsudo T.-A. (2000). Metal-catalyzed carbon-sulfur bond formation. Chem. Rev..

[6] Shen C., Zhang P., Sun Q., Bai S., Hor T.S.A., Liu X. (2015). Recent advances in C-S bond formation via C-H bond functionalization and decarboxylation. Chem. Soc. Rev..

[7] Dong D.-Q., Hao S.-H., Yang D.-S., Li L.-X., Wang Z.-L. (2017). Sulfenylation of C-H bonds for C-S bond formation under metal-free conditions. Eur. J. Org. Chem..

[8] Xiao F., Chen S., Li C., Huang H., Deng G.-J. (2016). Coppercatalyzed three-component one-pot synthesis of aryl sulfides with sulfur powder under aqueous conditions. Adv. Synth. Catal..

[9] Lin Y.-M., Lu G.-P., Wang G.-X., Yi W.-B. (2016). Odorless, regioselective synthesis of diaryl sulfides and α-thioaryl carbonyls from sodium arylsulfinates via a metal-free radical strategy in water. Adv. Synth. Catal..

[10] Feng Q., Chen D., Hong M., Wang F., Huang S. (2018). Phenyliodine (III) bis (trifluoroacetate)(PIFA)-mediated synthesis of aryl sulfides in water. J. Org. Chem..

[11] Saima S., Equbal D., Lavekar A.G., Sinha A.K. (2016). Cooperative catalysis by bovine serum albumin-iodine towards cascade oxidative coupling-C(sp^2^)-H sulfenylation of indoles/hydroxyaryls with thiophenols on water. Org. Biomol. Chem..

[12] Yang Z., Yan Y., Li A., Liao J., Zhang L., Yang T., Zhou C. (2018). Iodine-mediated sulfenylation of 4-hydroxycoumarins with sulfonyl hydrazides under aqueous conditions. New J. Chem..

[13] Huang X., Chen Y., Zhen S., Song L., Gao M., Zhang P., Li H., Yuan B., Yang G. (2018). Cobalt-catalyzed aerobic cross-dehydrogenative coupling of C-H and thiols in water for C-S formation. J. Org. Chem..

[14] Kumar N., Venkatesh R., Singh S., Kandasamy J. (2023). Potassium Persulfate-Glucose Mediated Synthesis of (3)-S-Arylthioindoles from Indole and Thiophenols in Water. Eur. J. Org. Chem..

[15] Kang X., Yan R., Yu G., Pang X., Liu X., Li X., Xiang L., Huang G. (2015). Iodine-mediated thiolation of substituted naphthols/naphthylamines and aryl sulfonyl hydrazides via C(sp^2^)-H bond functionalization. J. Org. Chem..

[16] Yueting W., Yali L., Jing H., Xuezhen L., Ping L., Jie Z. (2020). TBAI-mediated sulfenylation of arenes with arylsulfonyl hydrazides in DPDME. Tetrahedron.

[17] Makhayeva D.N., Irmukhametova G.S., Khutoryanskiy V.V. (2023). Advances in antimicrobial polymeric iodophors. Eur. Polym. J..

[18] Freeman C., Duan E., Kessler J. (2022). Molecular iodine is not responsible for cytotoxicity in iodophors. J. Hosp. Infect..

[19] Yang F.L., Tian S.K. (2013). Iodine-catalyzed regioselective sulfenylation of indoles with sulfonyl hydrazides. Angew. Chem. Int. Ed..

[20] Yang Y., Zhang S., Tang L., Hu Y., Zha Z., Wang Z. (2018). Catalyst-free thiolation of indoles with sulfonyl hydrazides for the synthesis of 3-sulfenylindoles in water. Green. Chem..

[21] Zhan Z., Ma H., Wei D., Pu J., Zhang Y., Huang G. (2018). Metal-free catalyzed synthesis of the (E)-vinyl sulfones via aromatic olefins with arylsulfonyl hydrazides. Tetrahedron Lett..

[22] Jia F., He J., Wei Y., Liu Y., Gu Y., Vaccaro L., Liu P. (2022). C4-sulfenylation of 4-iodine-1*H*-pyrazole-5-amine with arylsulfonyl hydrazide in water. Mol. Catal..

