# Supplementary material for: Iodophor-Catalyzed Disulfenylation of Amino Naphthalenes with Aryl Sulfonyl Hydrazines
Source: Molecules. 2024 May 21;29(11):2411. doi: 10.3390/molecules29112411 (PMC11173667; doi:10.3390/molecules29112411)
Supplement: Supplementary file 1 [file molecules-29-02411-s001.zip › molecules-2982999-supplementary.pdf]

Supporting Information for

Iodophor-Catalyzed Disulfenylation of Amino Naphthalenes with Aryl Sulfonyl Hydrazines

Yutong Yuan, Jing He, Xiaowei Ma *, Sheng Han and Yan Liu *

State Key Laboratory Incubation Base for Green Processing of Chemical Engineering, School of Chemistry and Chemical Engineering, Shihezi University, Shihezi 832004, China; 18616149673@163.com (Y.Y.); hejing@shzu.edu.cn (J.H.); hansheng654321@sina.com (S.H.)

* Correspondence: mxw_tea@shzu.edu.cn (X.M.); liuyan@shzu.edu.cn (Y.L.); Tel.: +86-0993-2057213 (Y.L.)

Copies of ^{1}H NMR and ^{13}C NMR spectra…………S1–S58

1,3-bis(phenylthio)naphthalen-2-amine (3a)

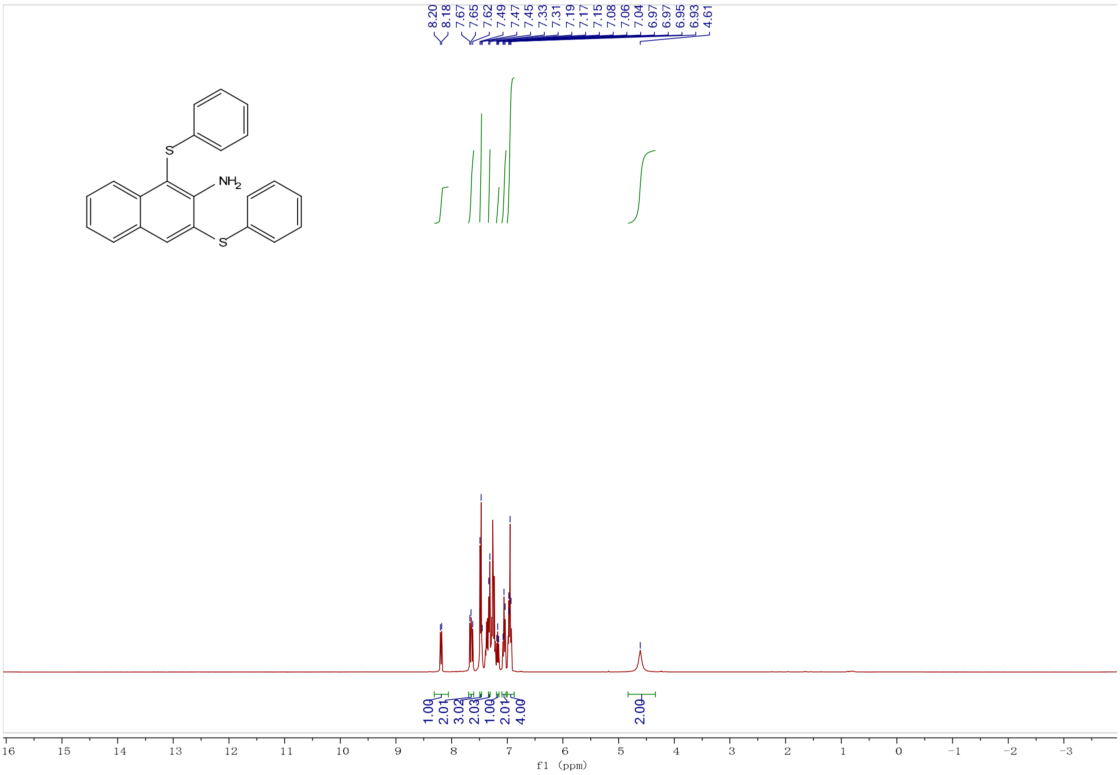

Figure S1

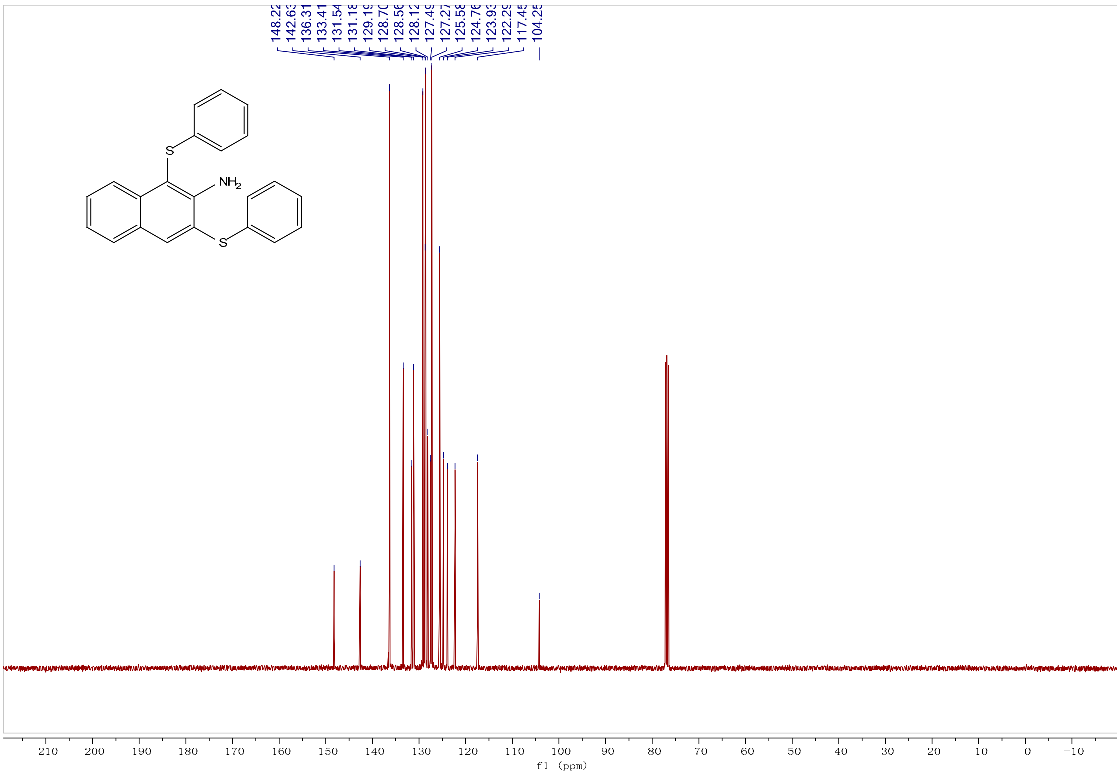

Figure S2

1,3-bis((4-methoxyphenyl)thio)naphthalen-2-amine (3b)

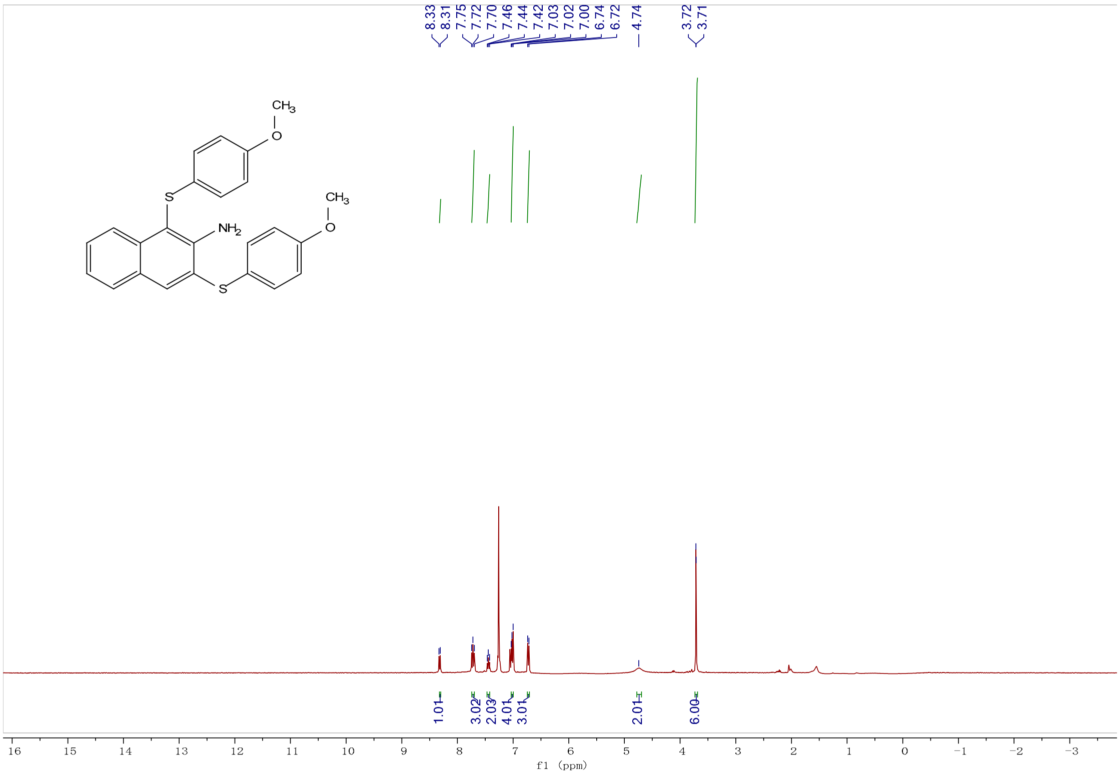

Figure S3

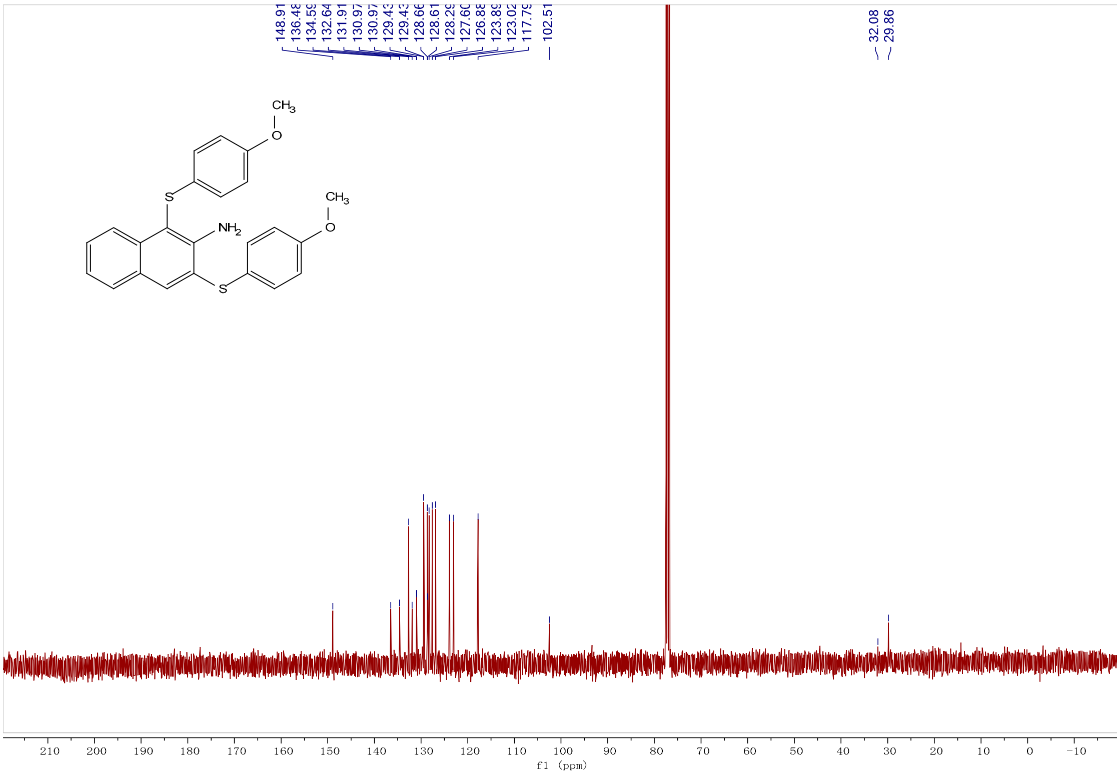

Figure S4

1,3-bis((4-fluorophenyl)thio)naphthalen-2-amine (3c)

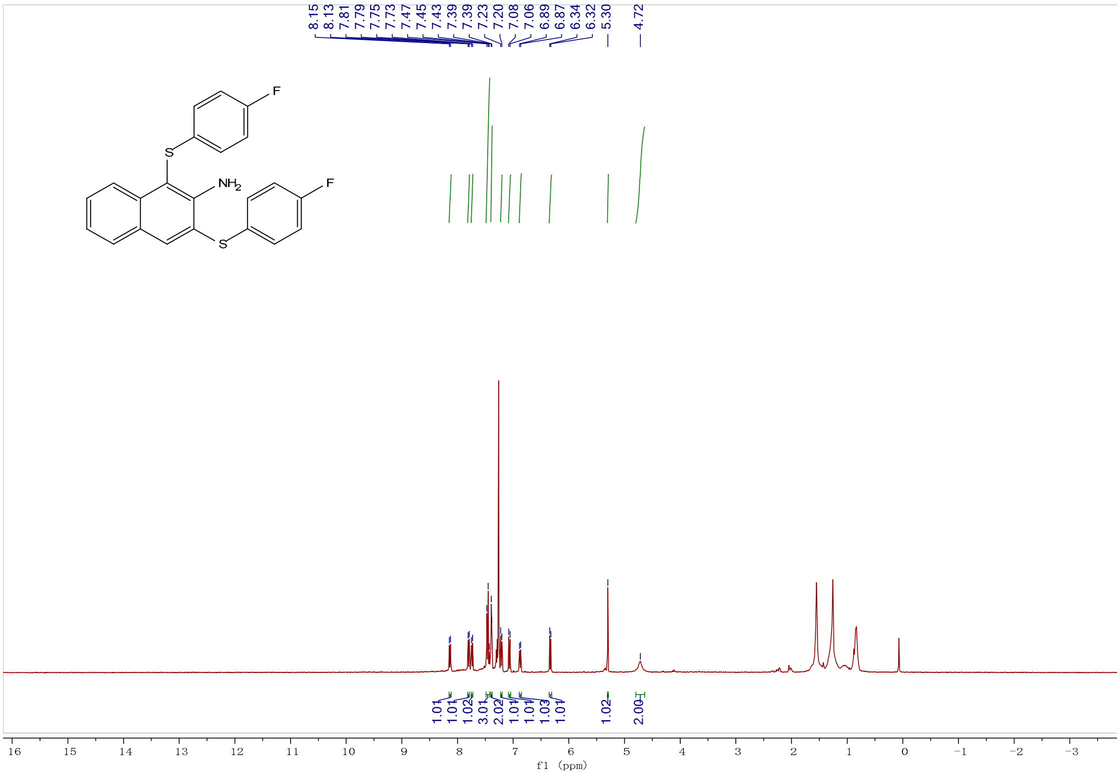

Figure S5

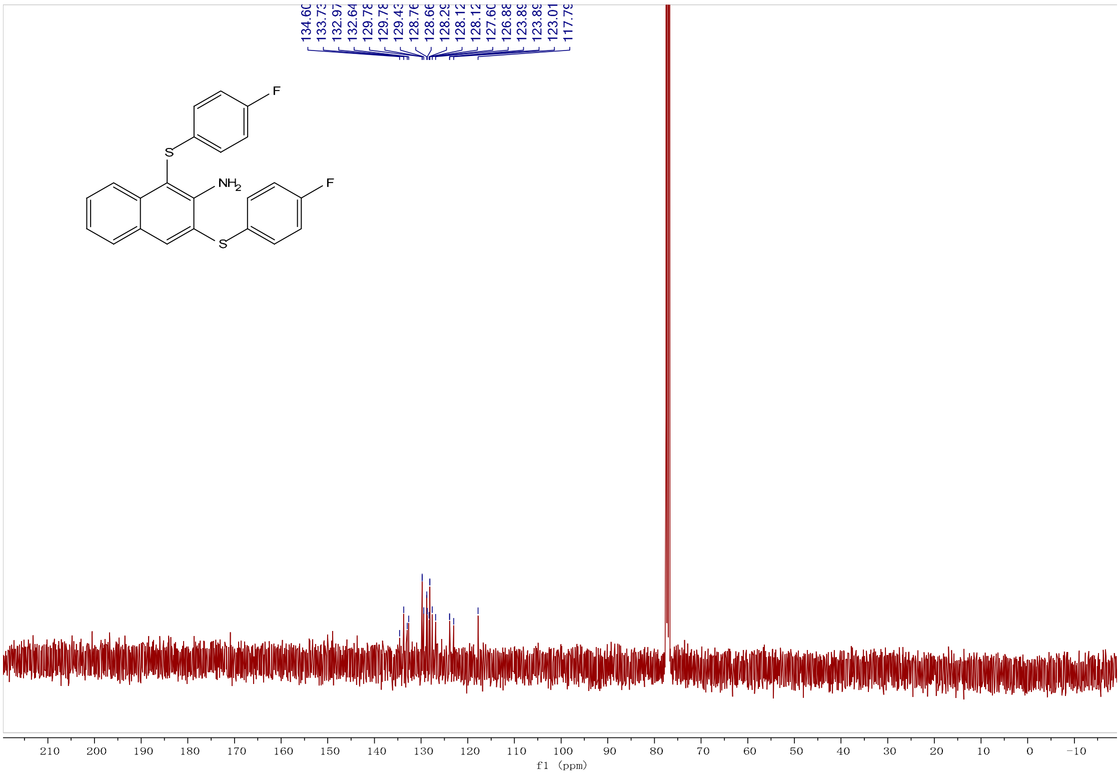

Figure S6

1,3-bis((4-chlorophenyl)thio)naphthalen-2-amine (3d)

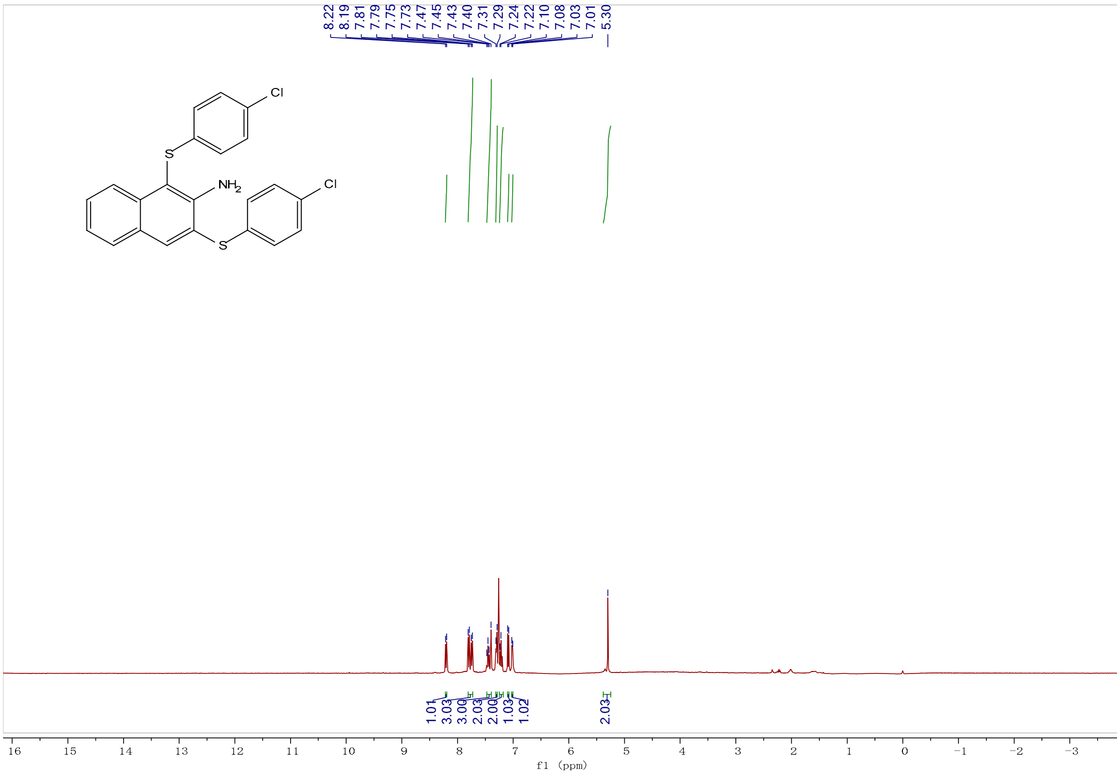

Figure S7

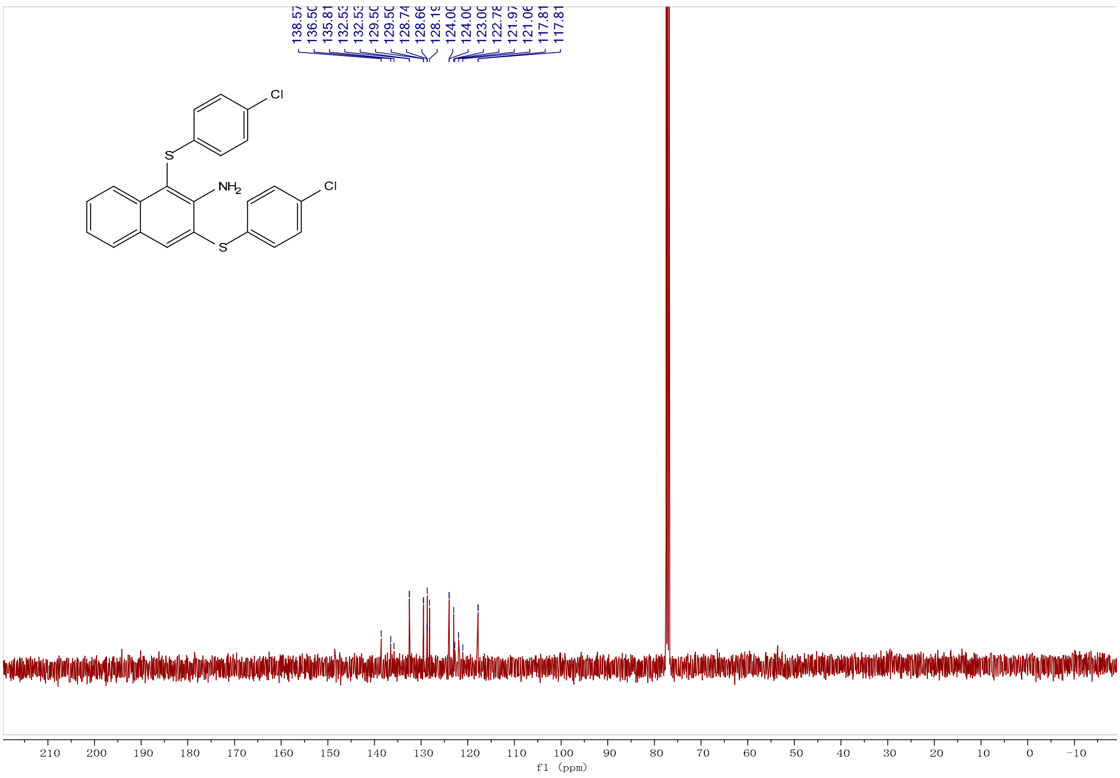

Figure S8

1,3-bis((4-bromophenyl)thio)naphthalen-2-amine (3e)

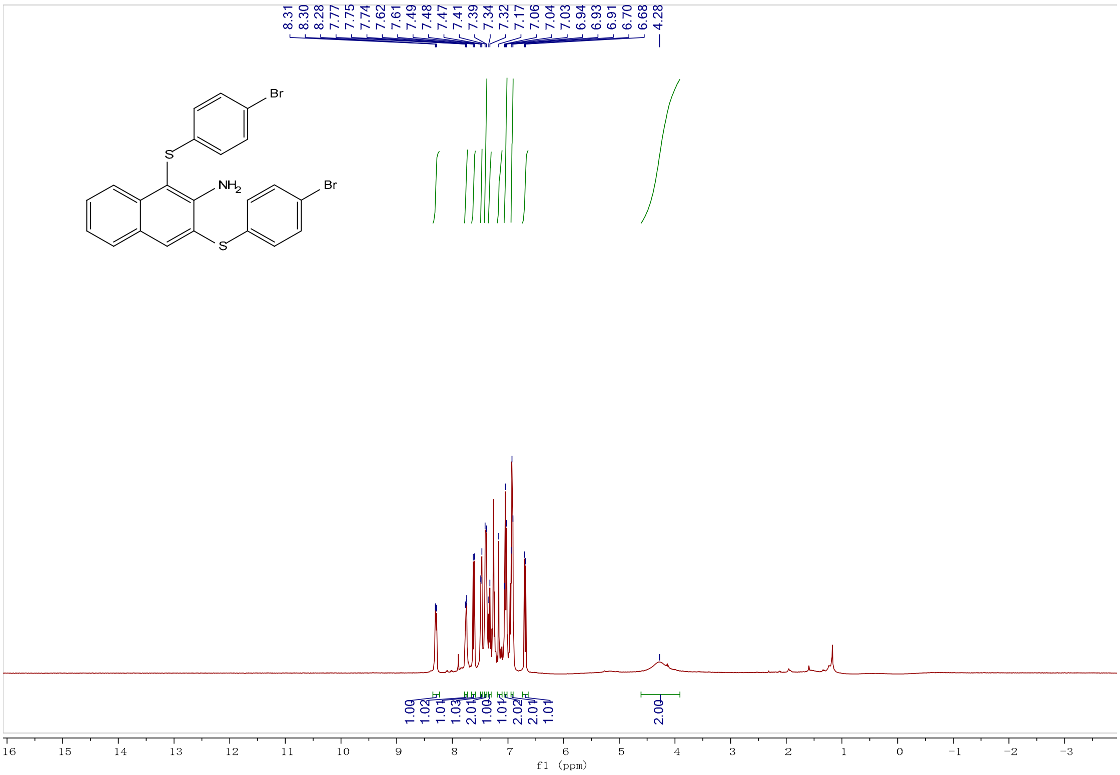

Figure S9

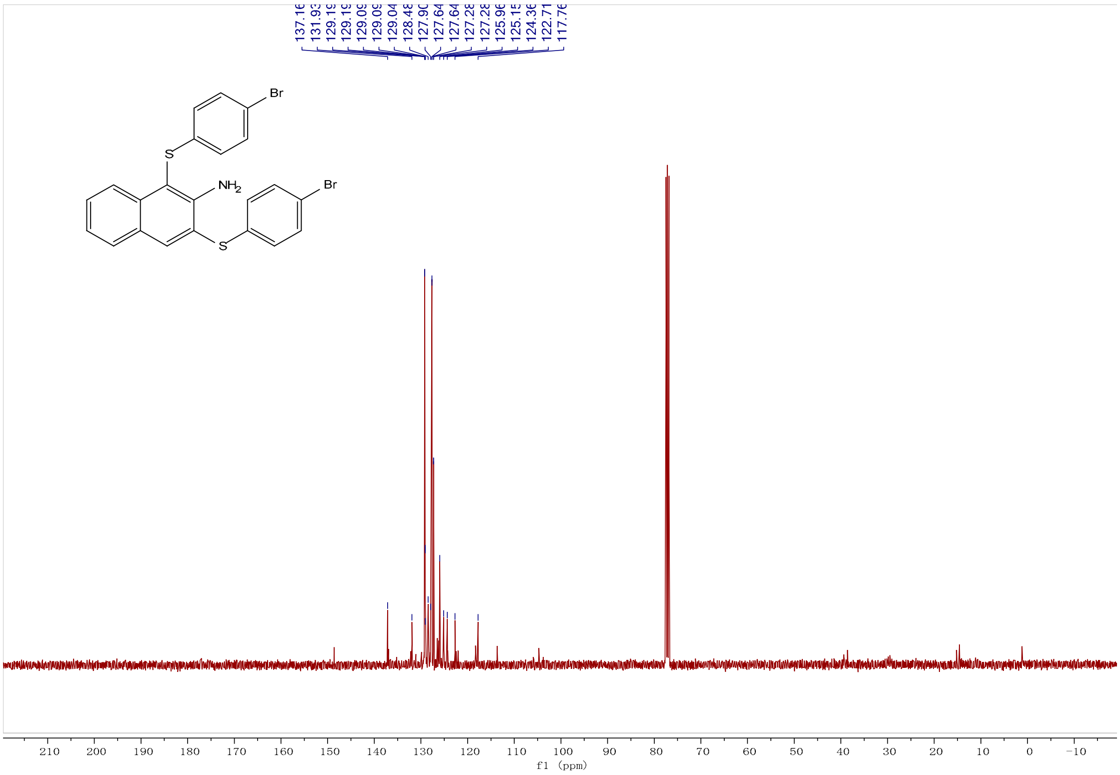

Figure S10

1,3-bis((4-(trifluoromethyl)phenyl)thio)naphthalen-2-amine (3f)

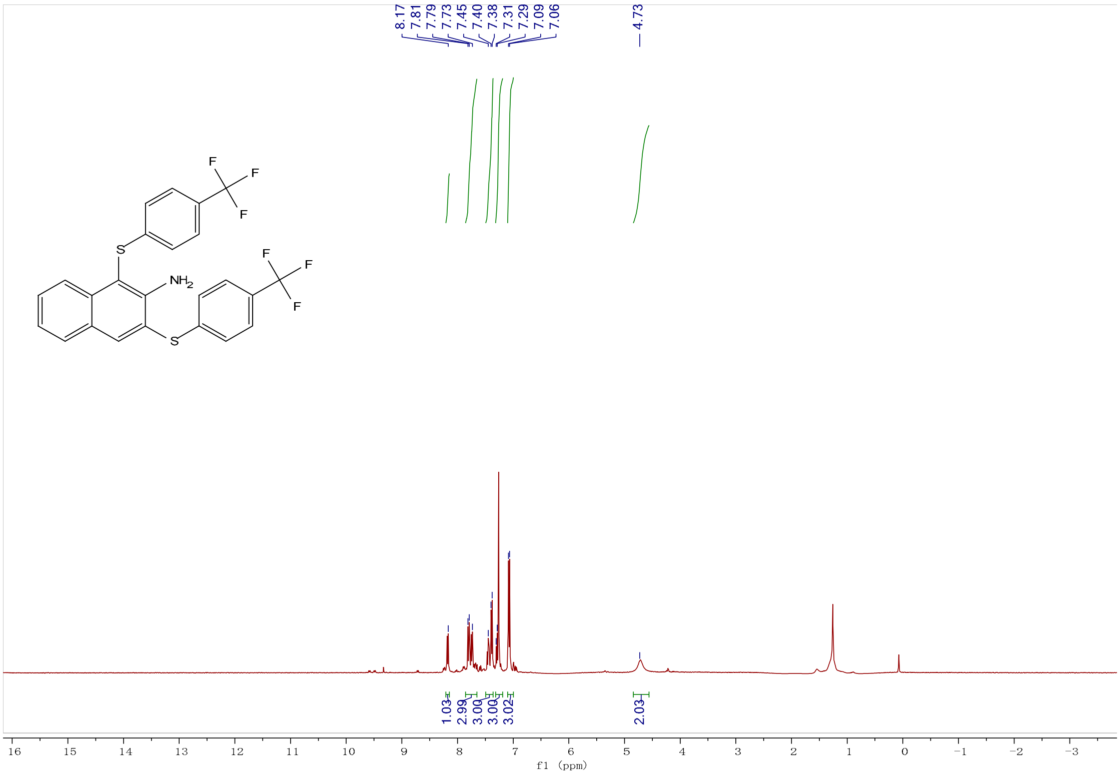

Figure S11

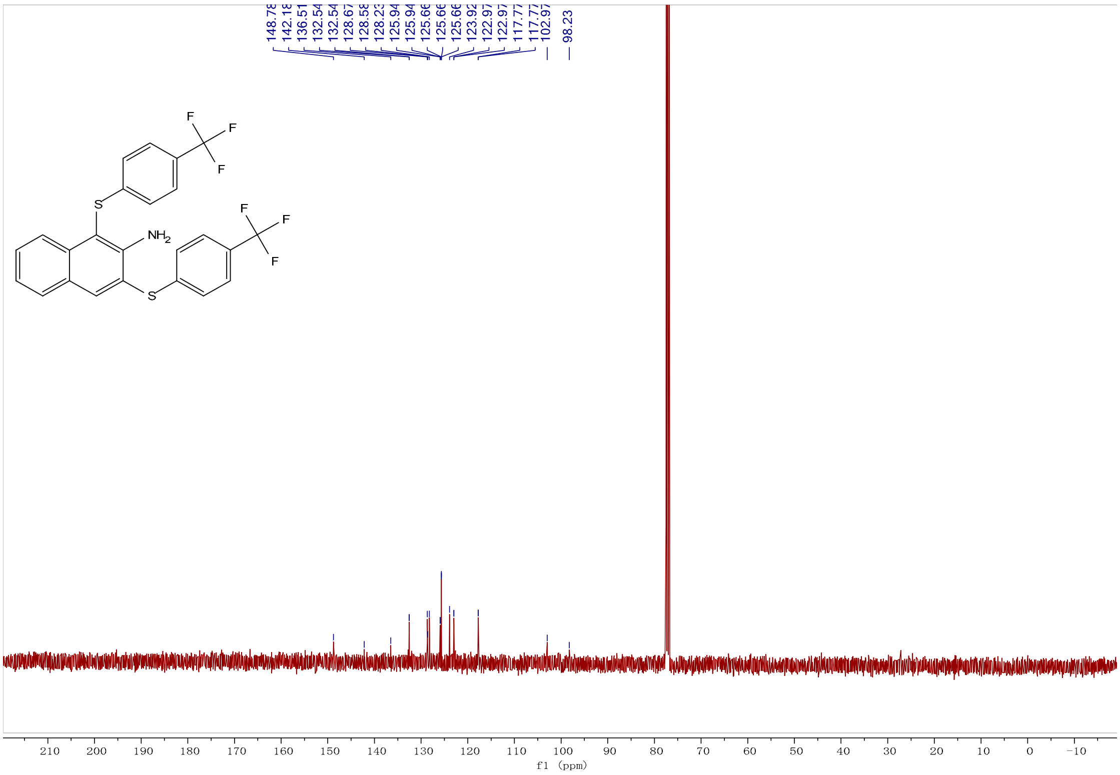

Figure S12

1,3-bis((4-(tert-butyl)phenyl)thio)naphthalen-2-amine (3g)

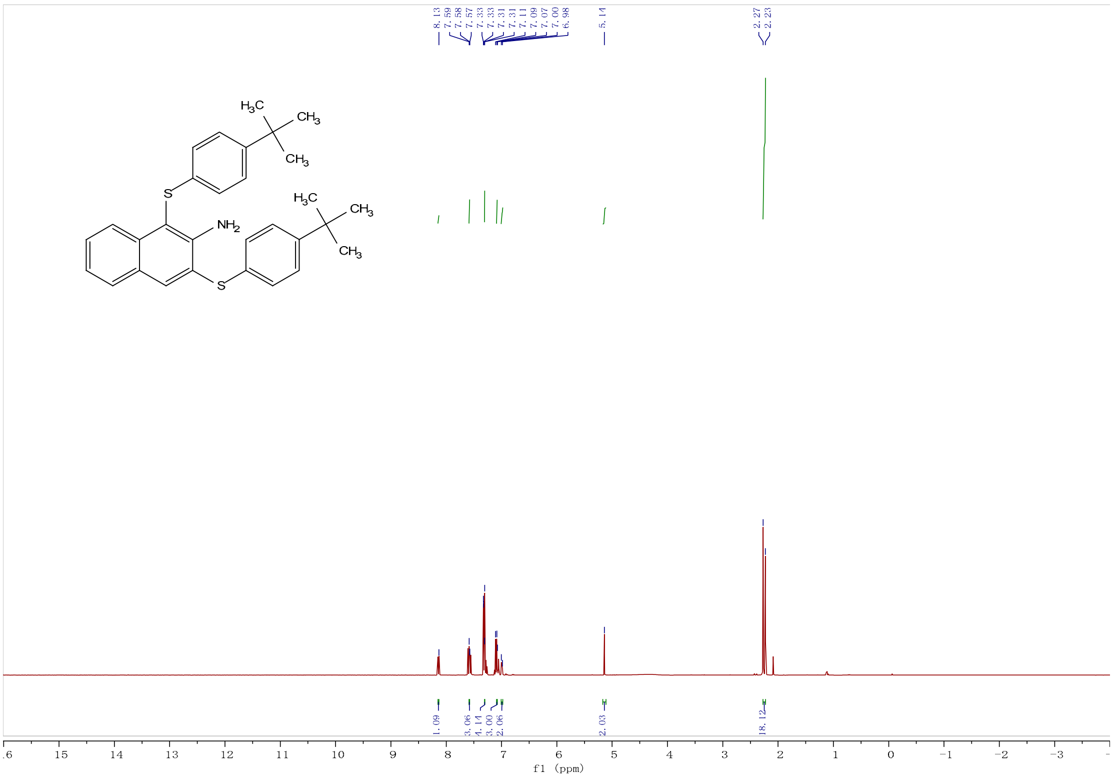

Figure S13

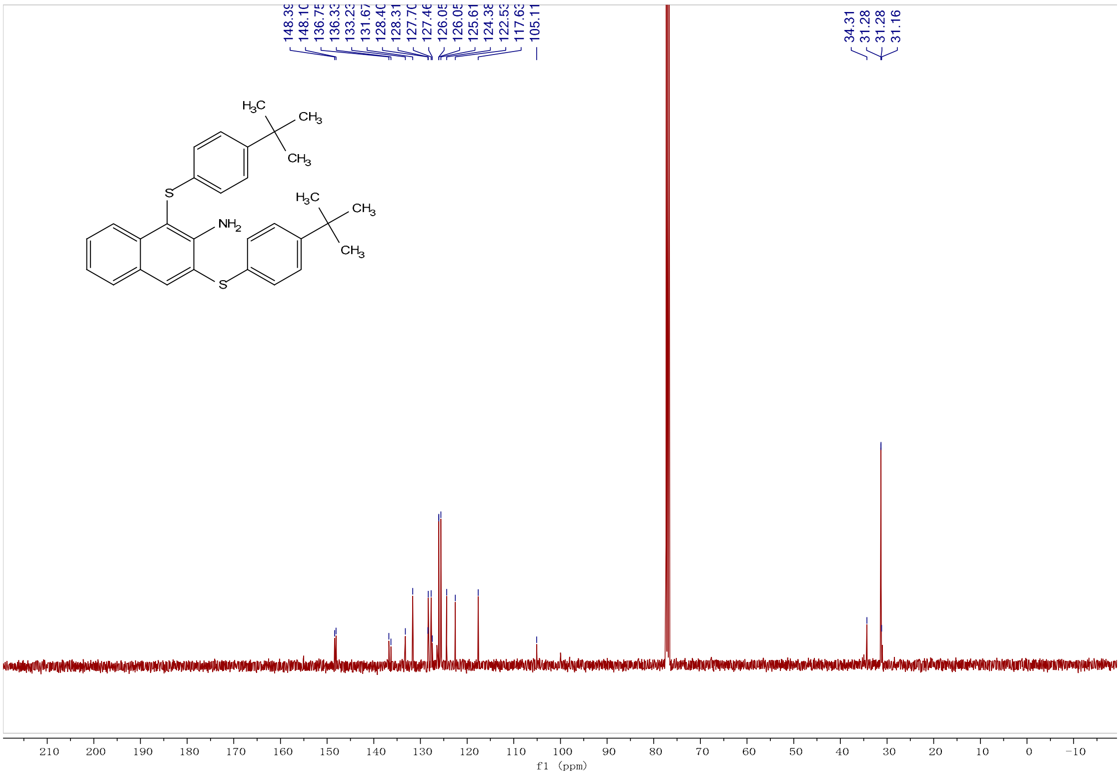

Figure S14

1,3-bis(p-tolylthio)naphthalen-2-amine (3h)

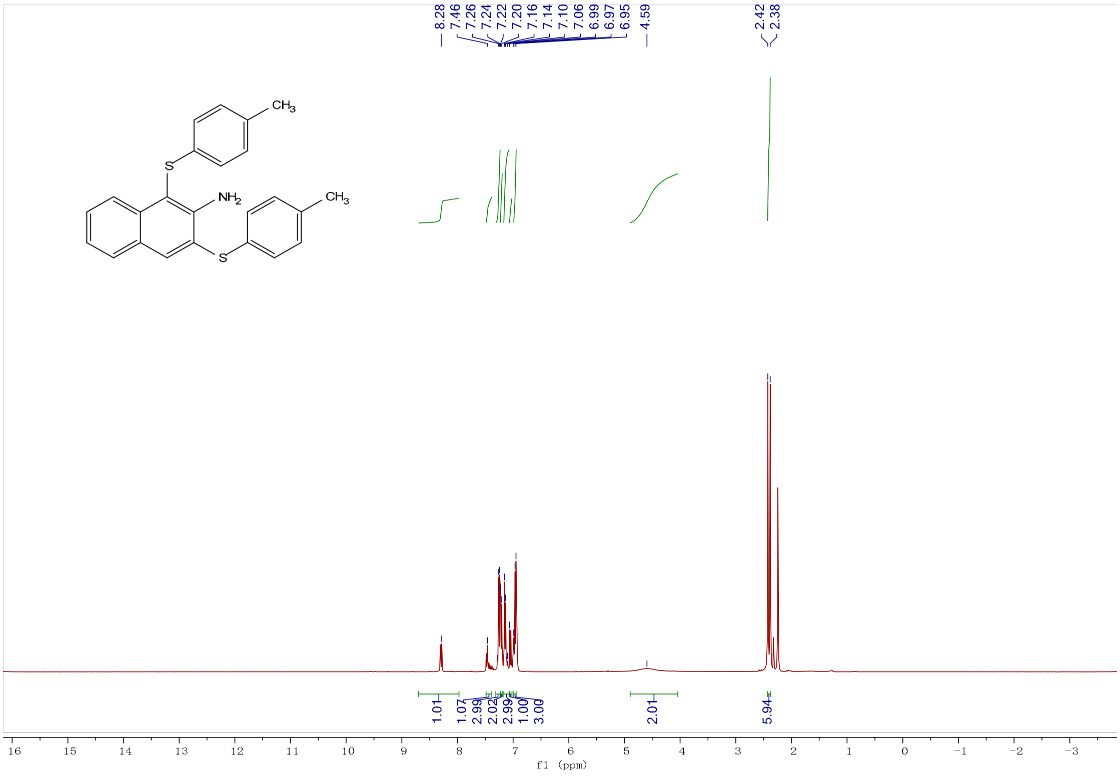

Figure S15

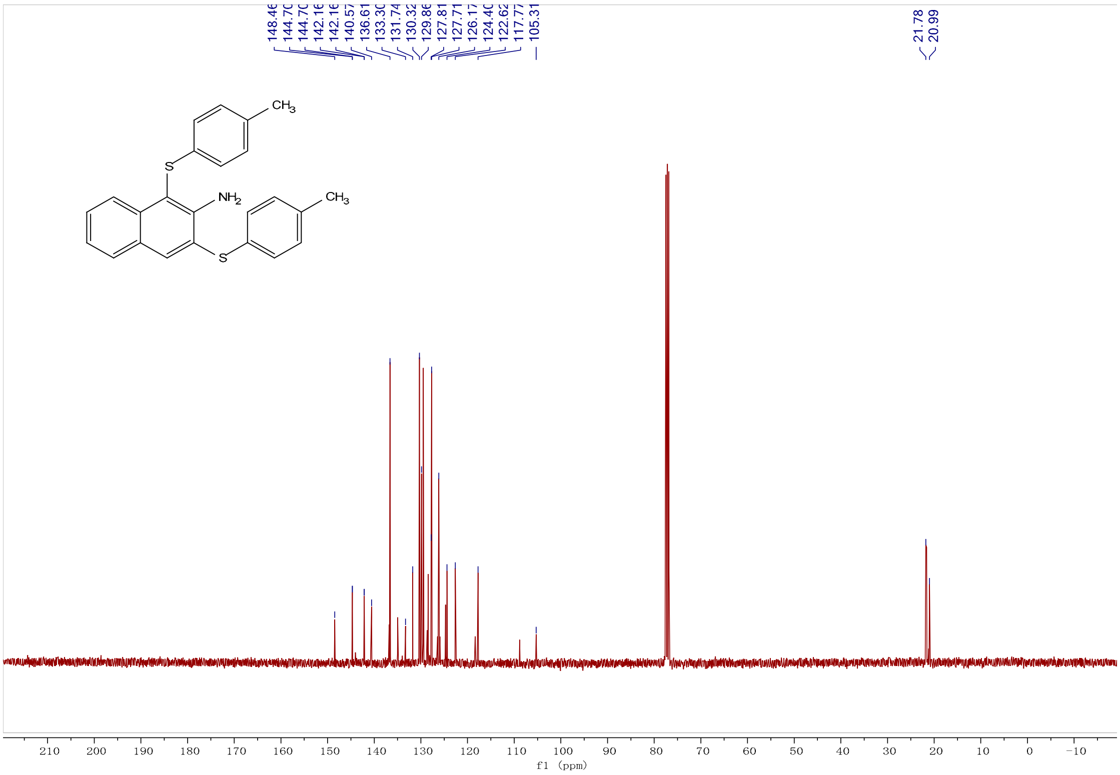

Figure S16

1,3-bis((3-bromophenyl)thio)naphthalen-2-amine (3i)

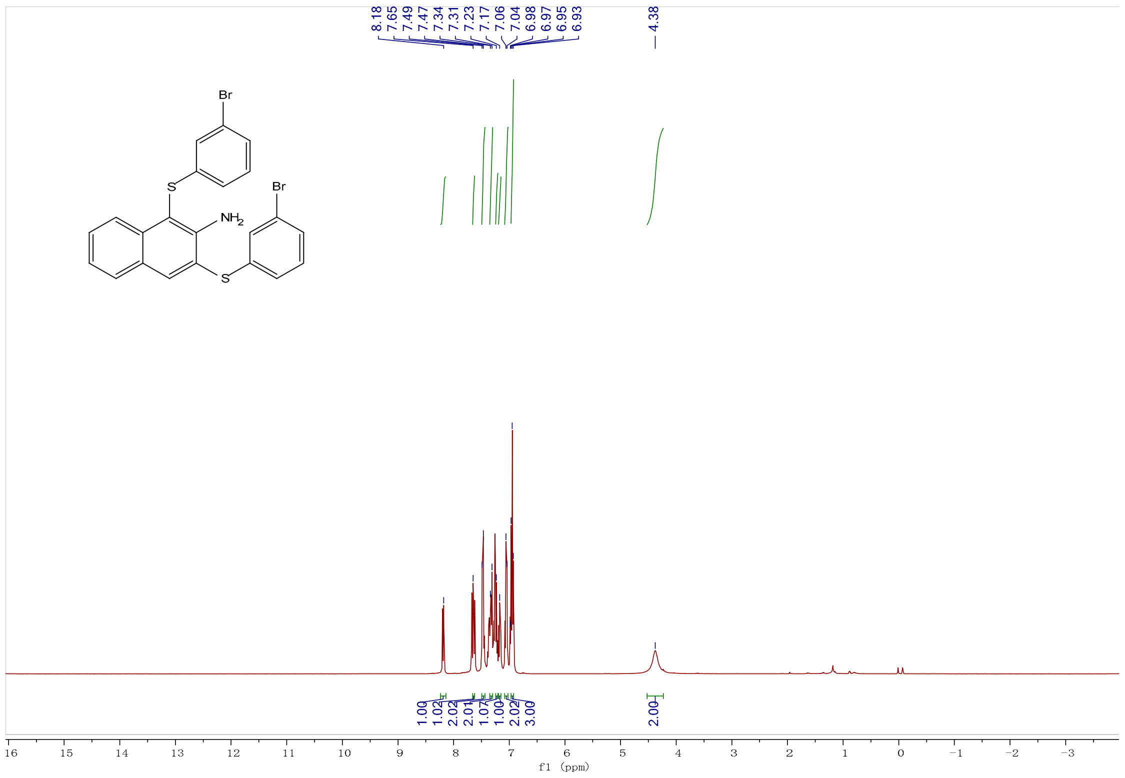

Figure S17

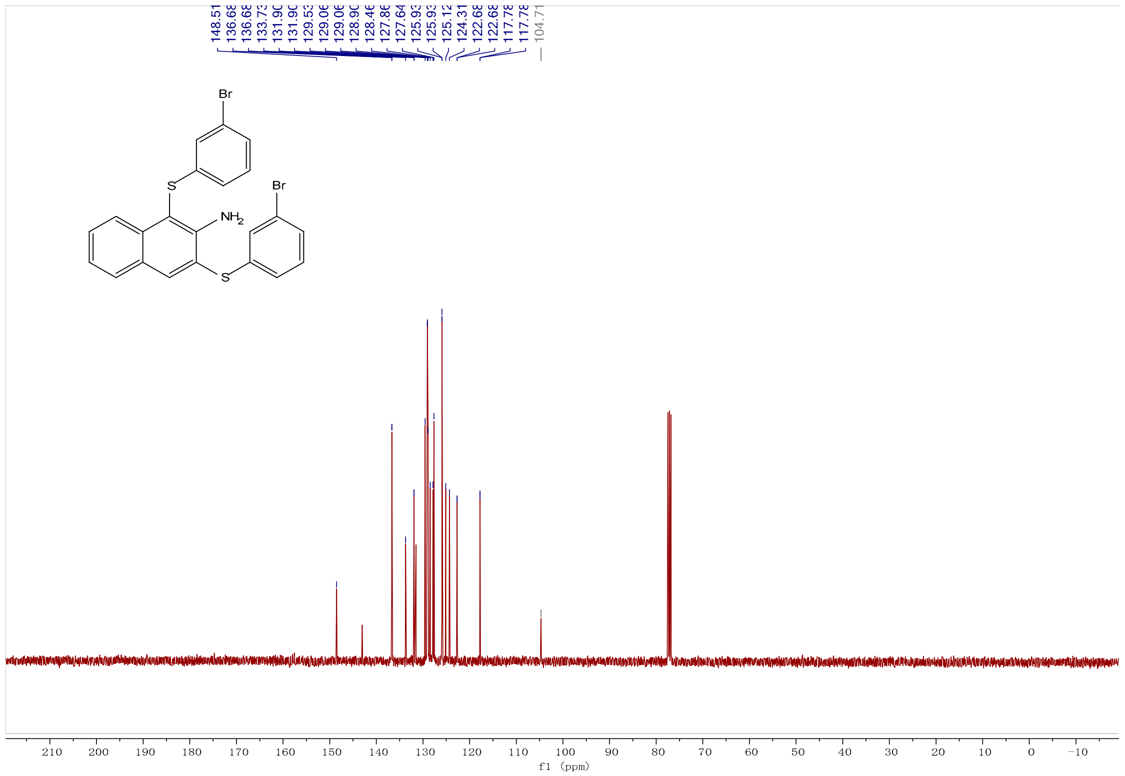

Figure S18

1,3-bis((3-(trifluoromethyl)phenyl)thio)naphthalen-2-amine (3j)

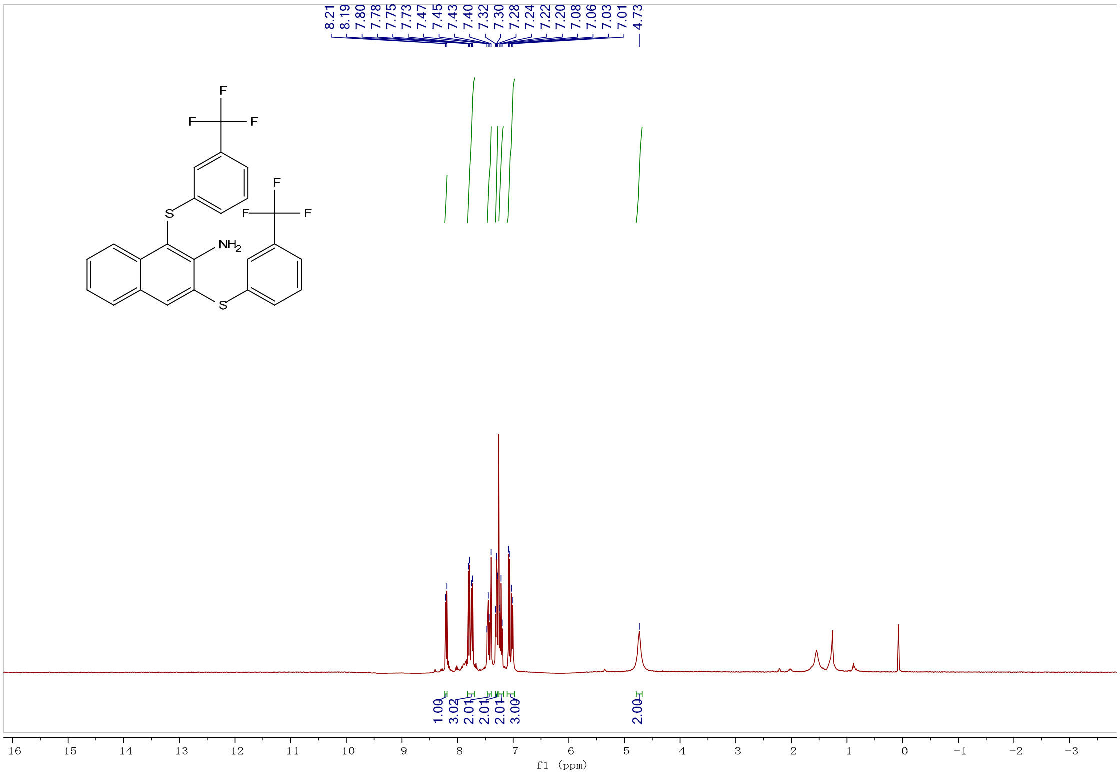

Figure S19

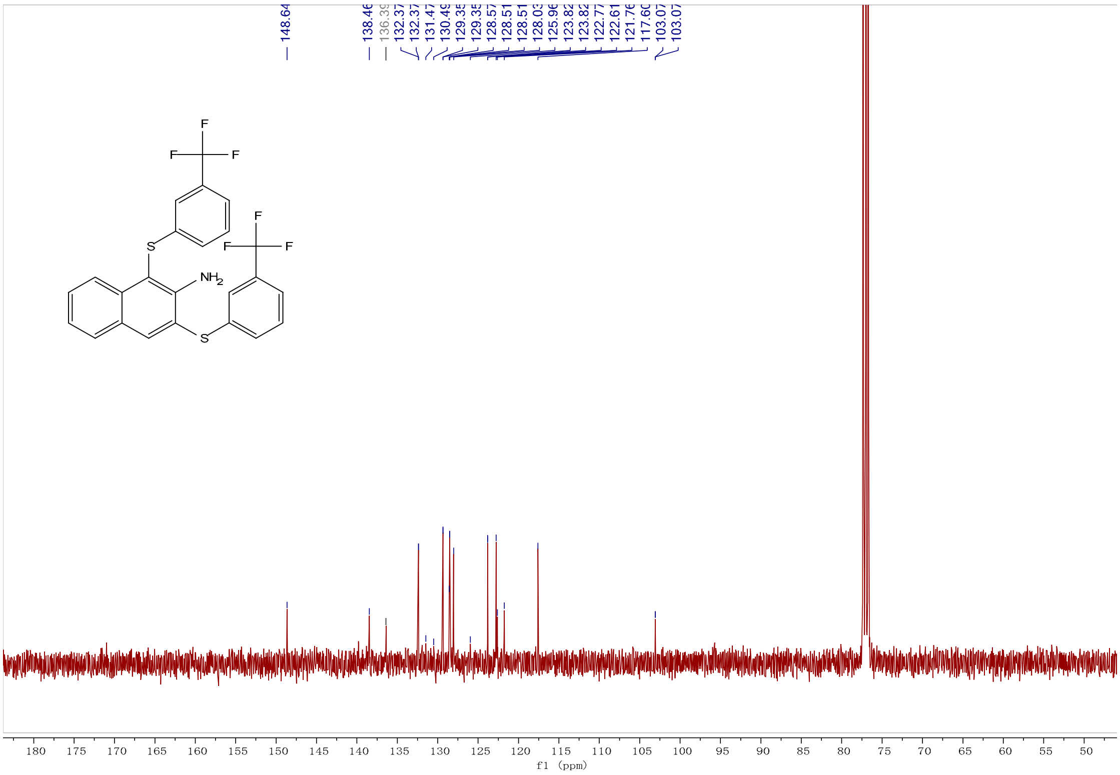

Figure S20

1,3-bis(o-tolylthio)naphthalen-2-amine (3k)

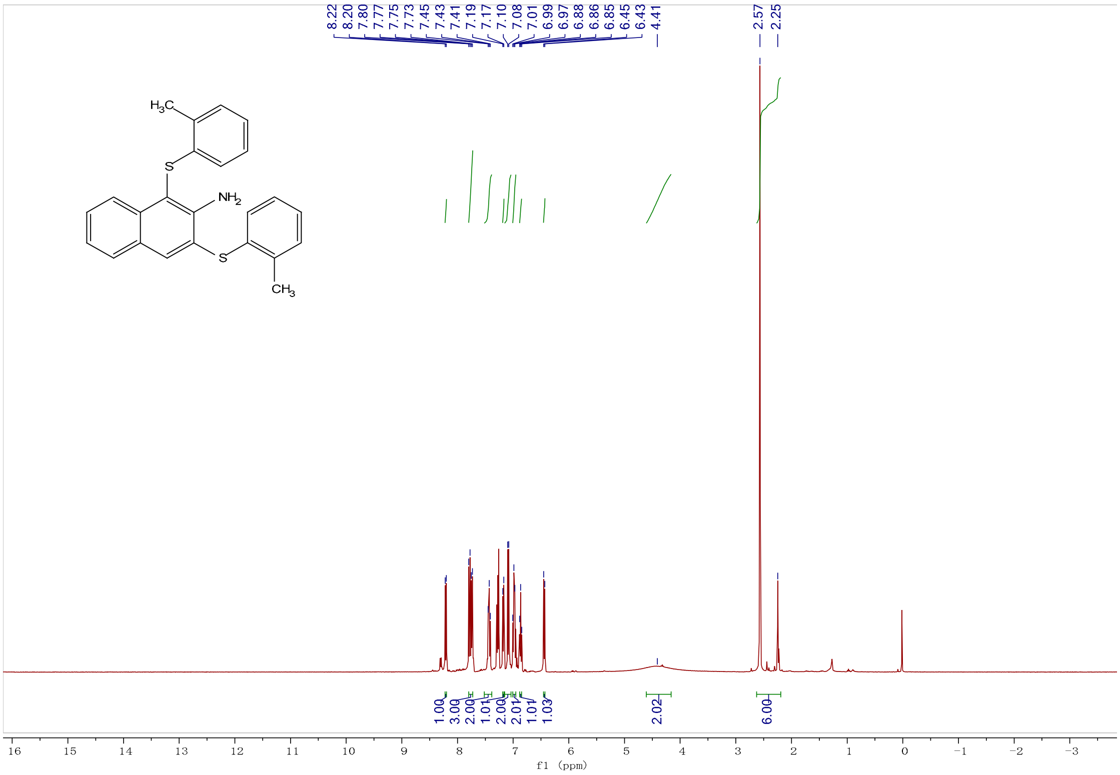

Figure S21

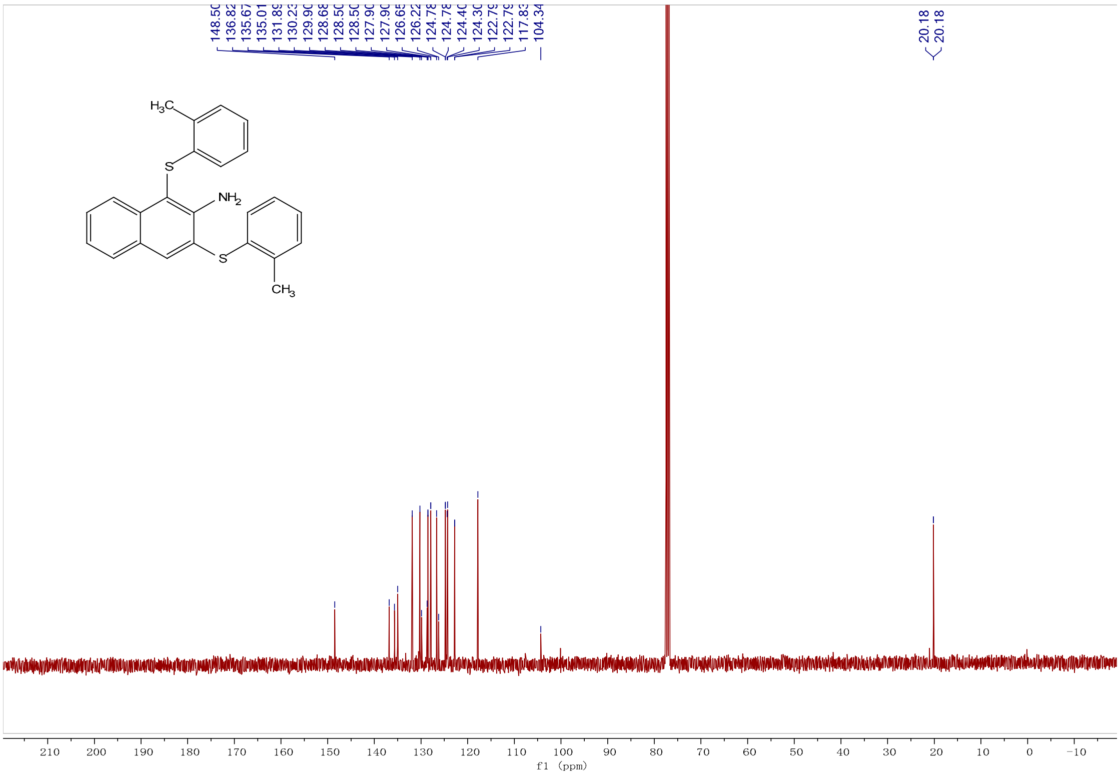

Figure S22

1,3-bis((2-fluorophenyl)thio)naphthalen-2-amine (3l)

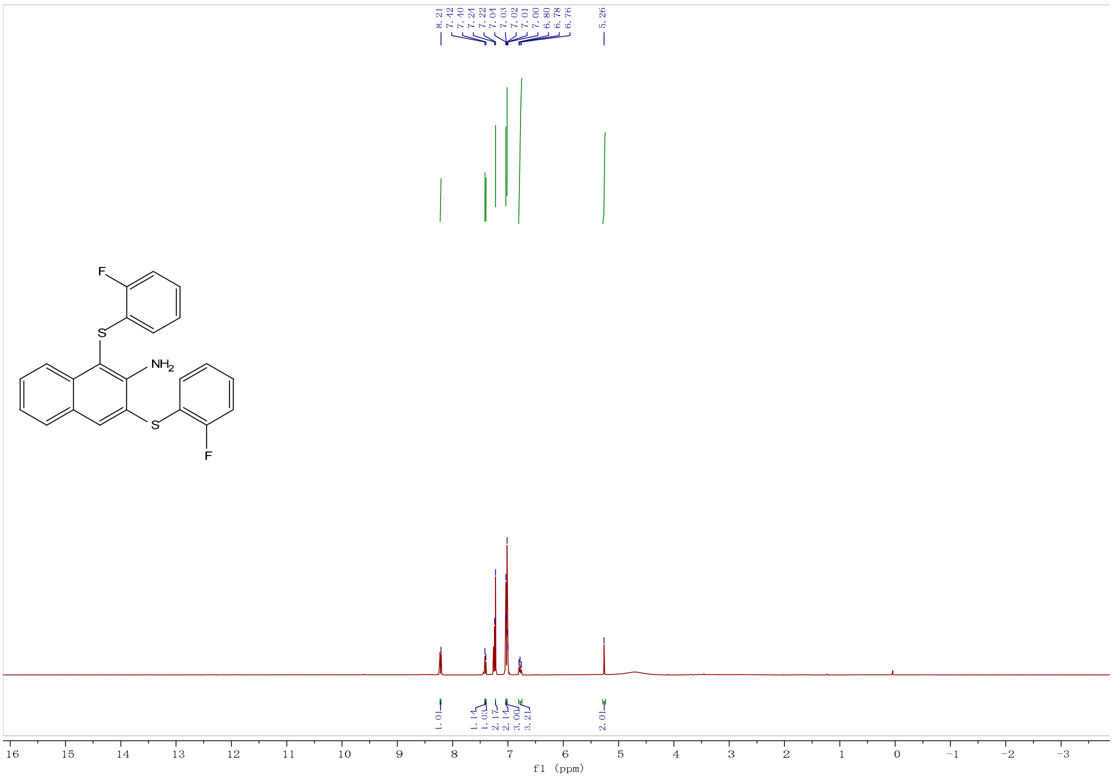

Figure S23

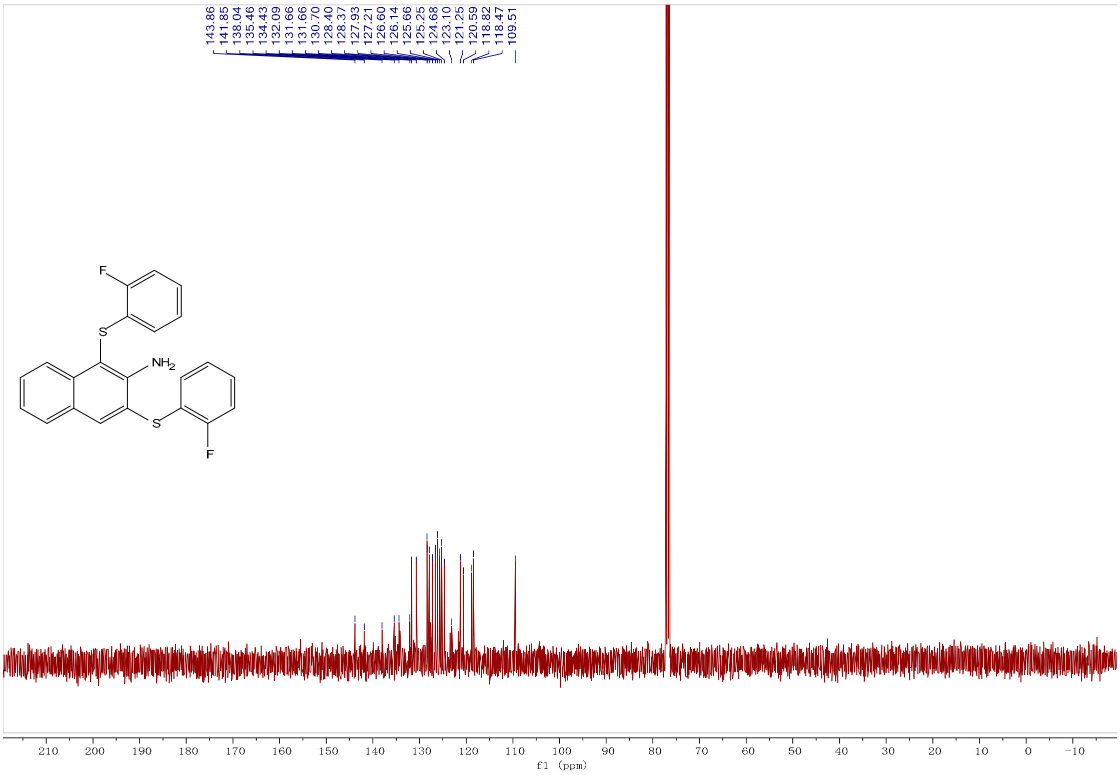

Figure S24

1,3-bis((2,4-dimethylphenyl)thio)naphthalen-2-amine (3m)

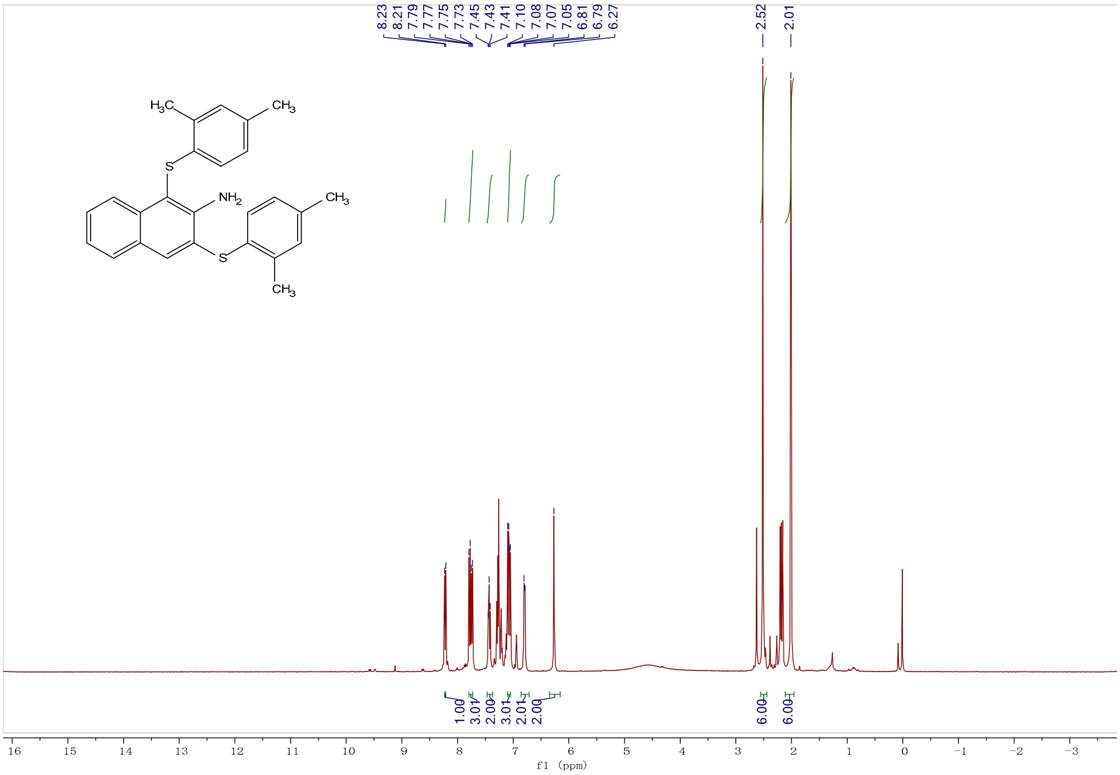

Figure S25

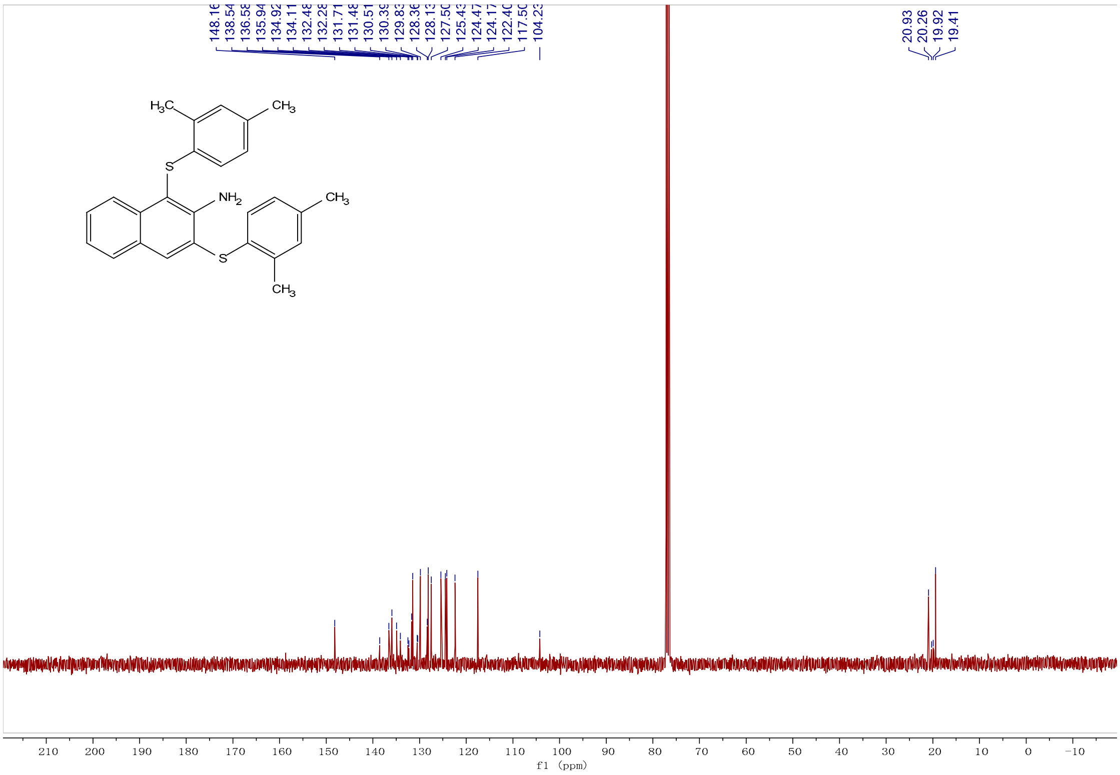

Figure S26

1,3-bis((2,4-dichlorophenyl)thio)naphthalen-2-amine (3n)

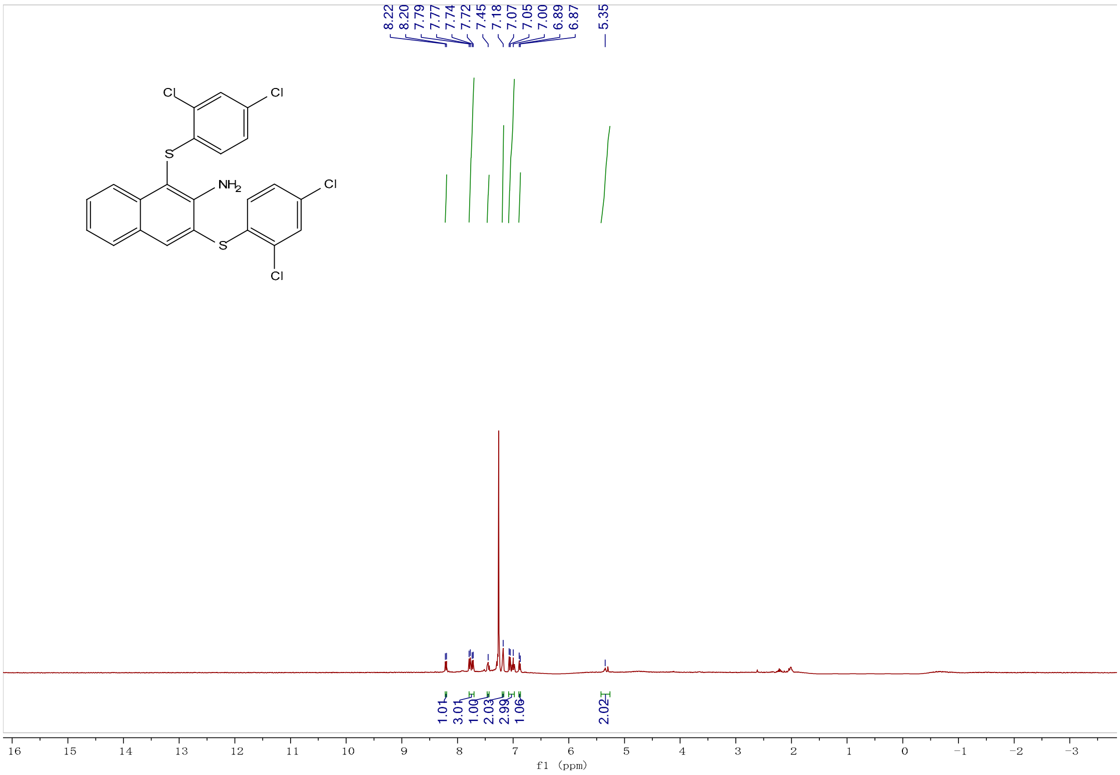

Figure S27

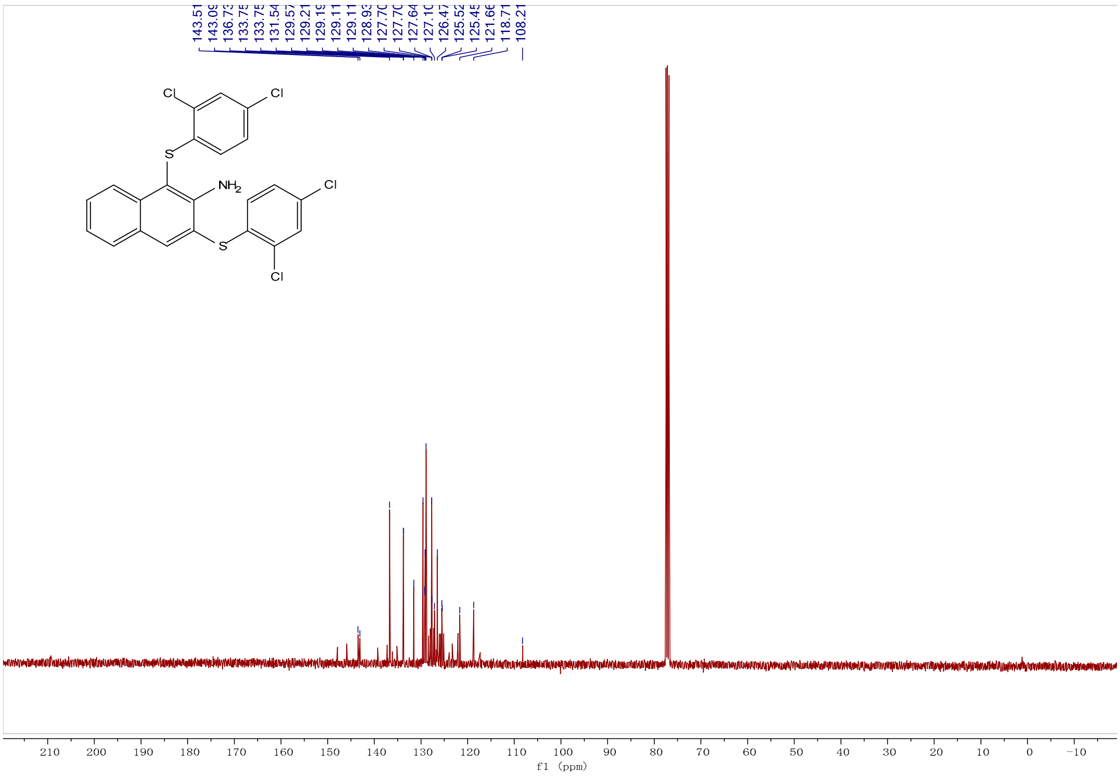

Figure S28

1,3-bis(thiophen-2-ylthio)naphthalen-2-amine (3o)

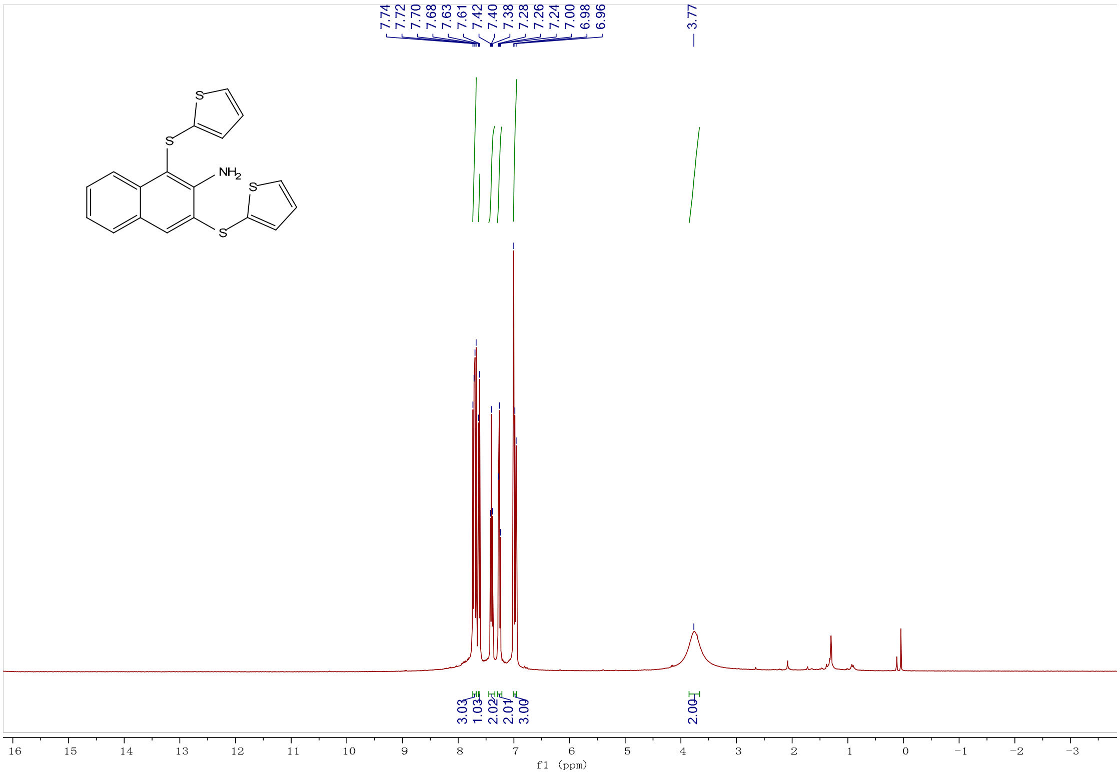

Figure S29

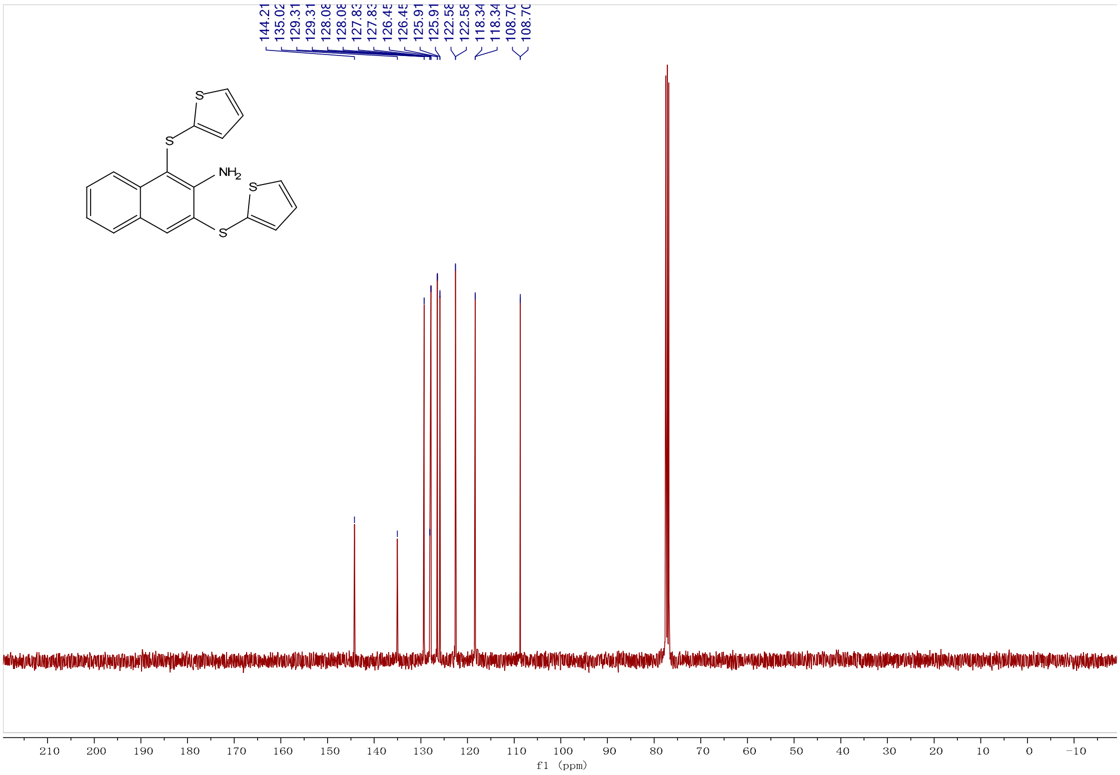

Figure S30

6-bromo-1,3-bis(phenylthio)naphthalen-2-amine (3p)

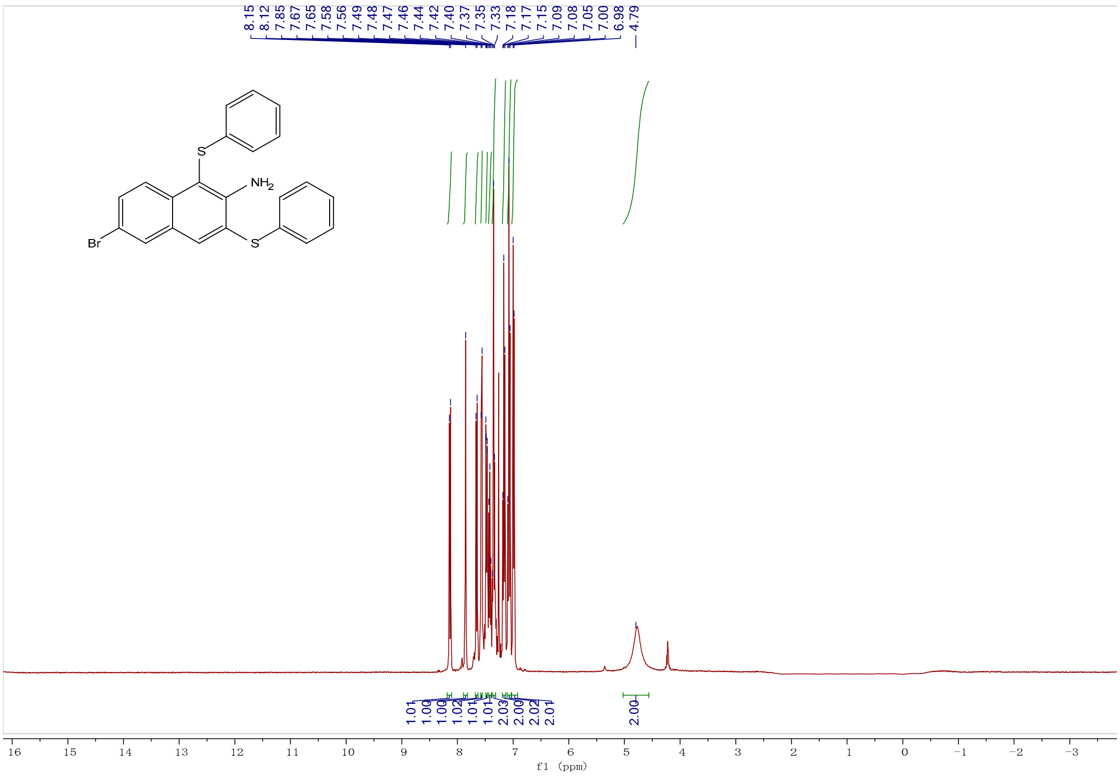

Figure S31

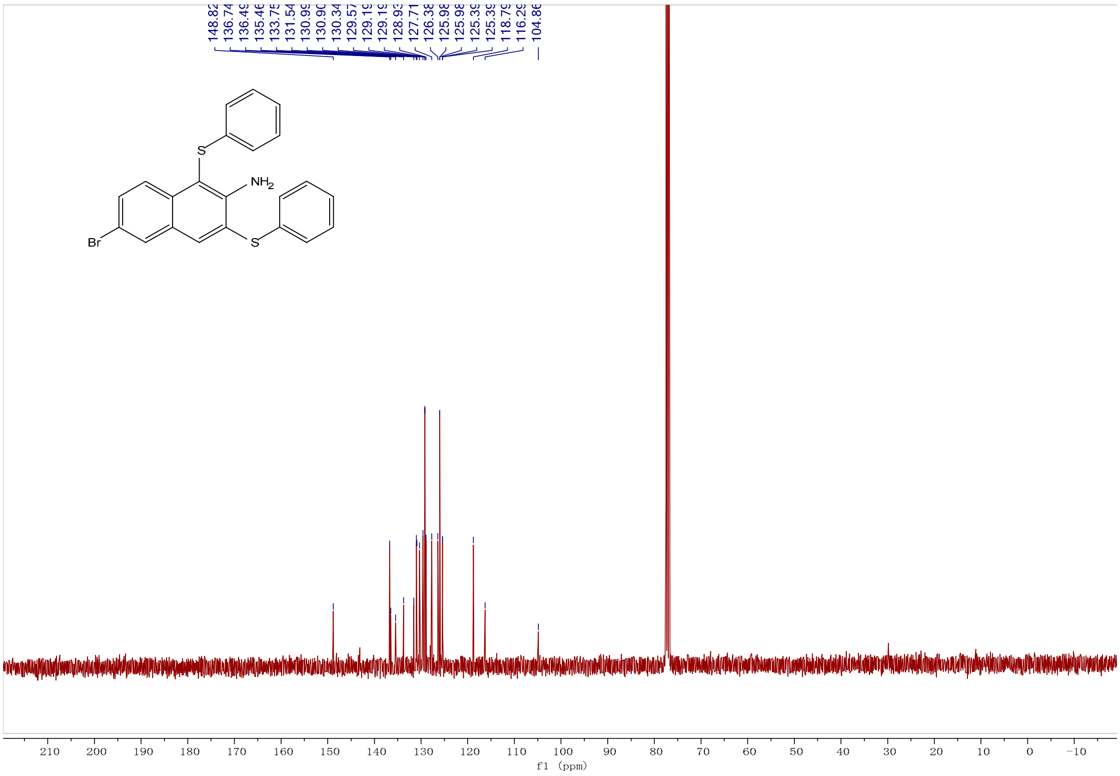

Figure S32

2,4-bis(phenylthio)naphthalen-1-amine (4a)

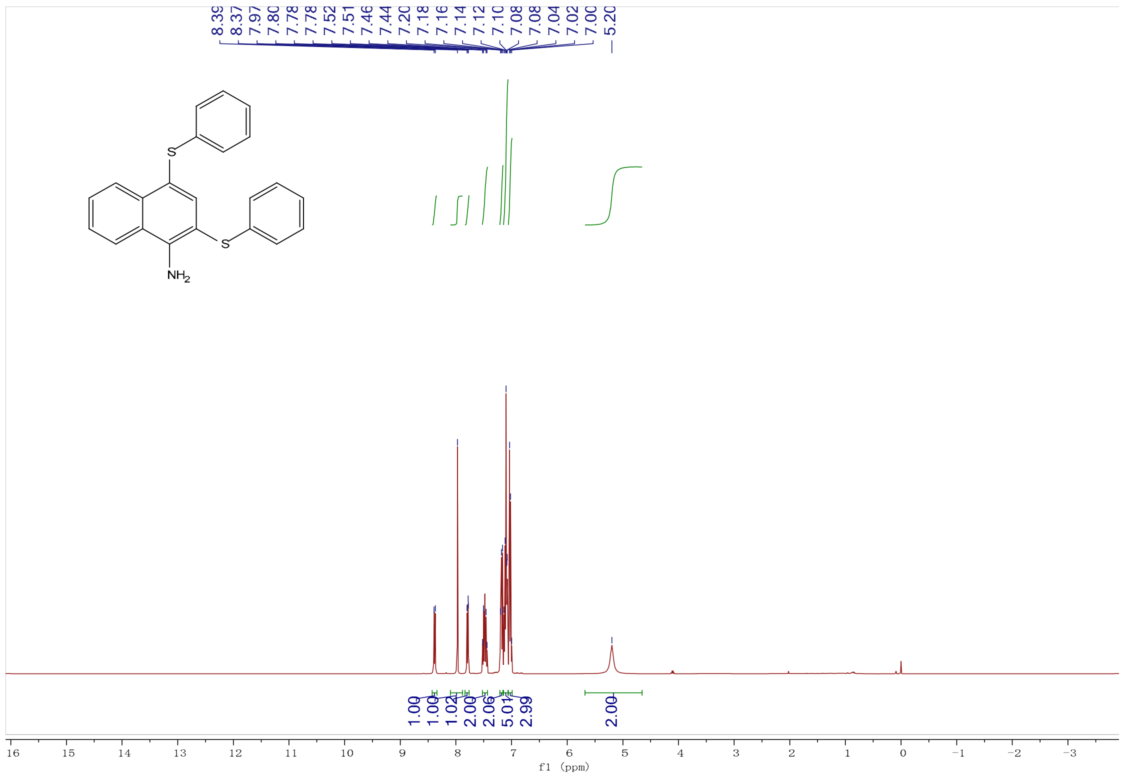

Figure S33

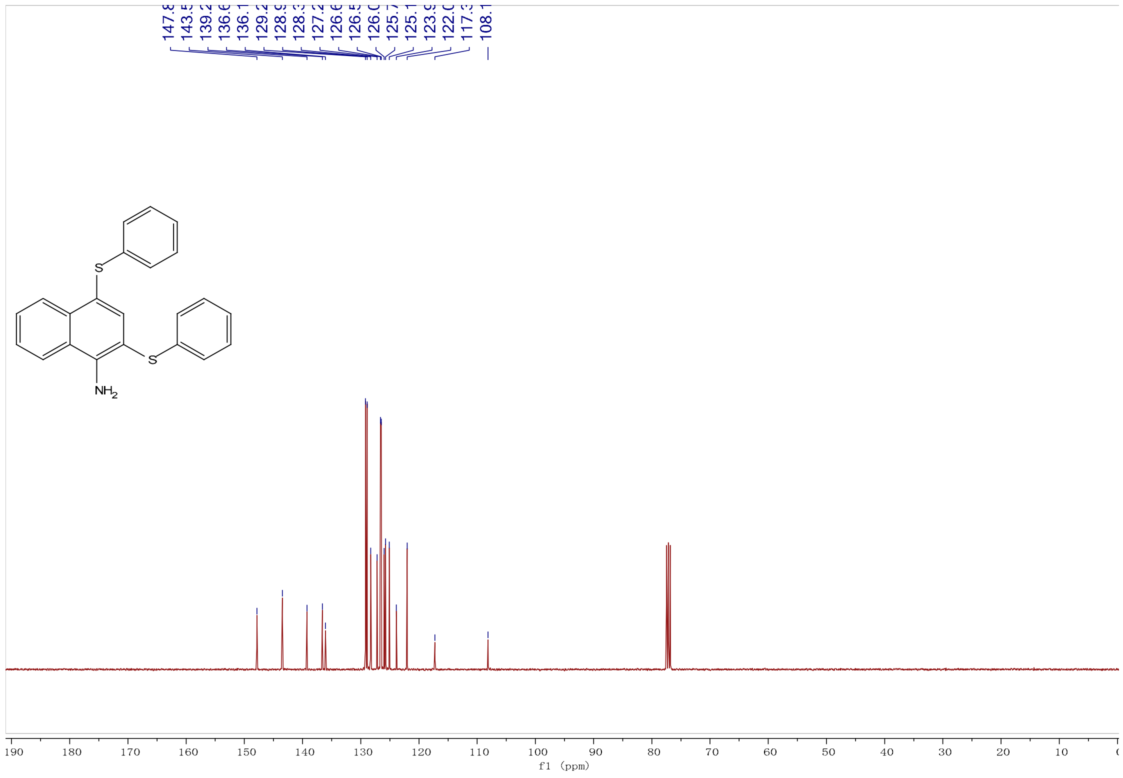

Figure S34

2,4-bis(p-tolylthio)naphthalen-1-amine (4b)

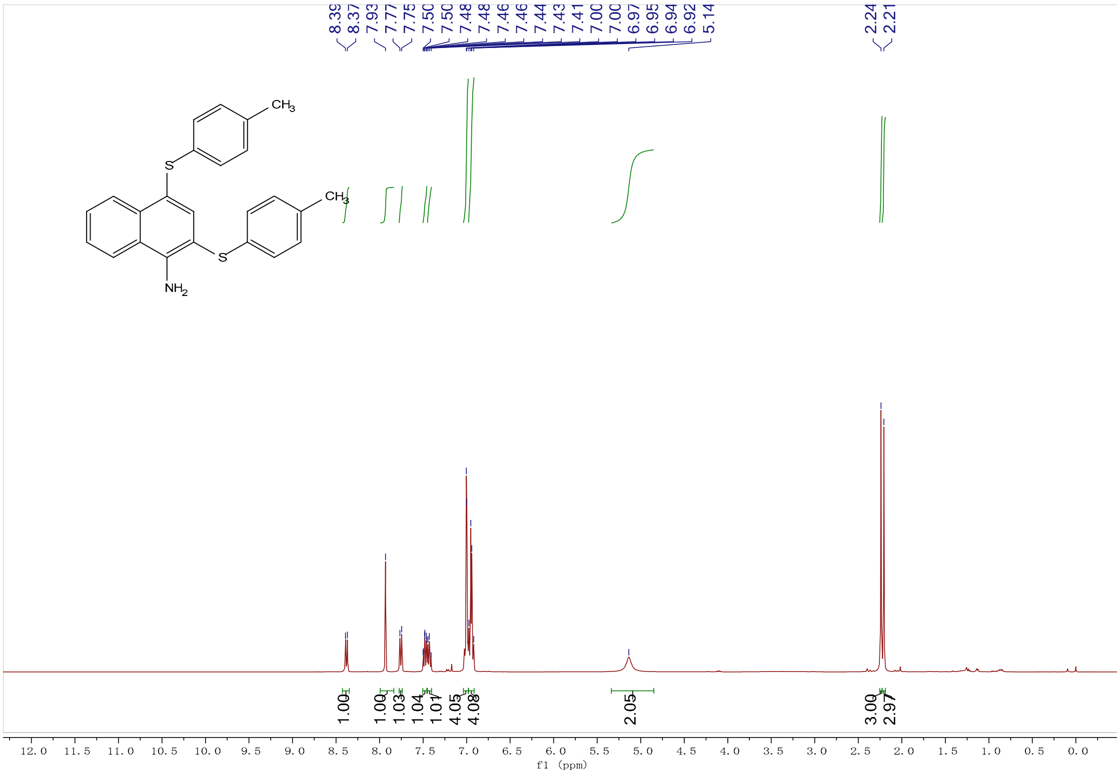

Figure S35

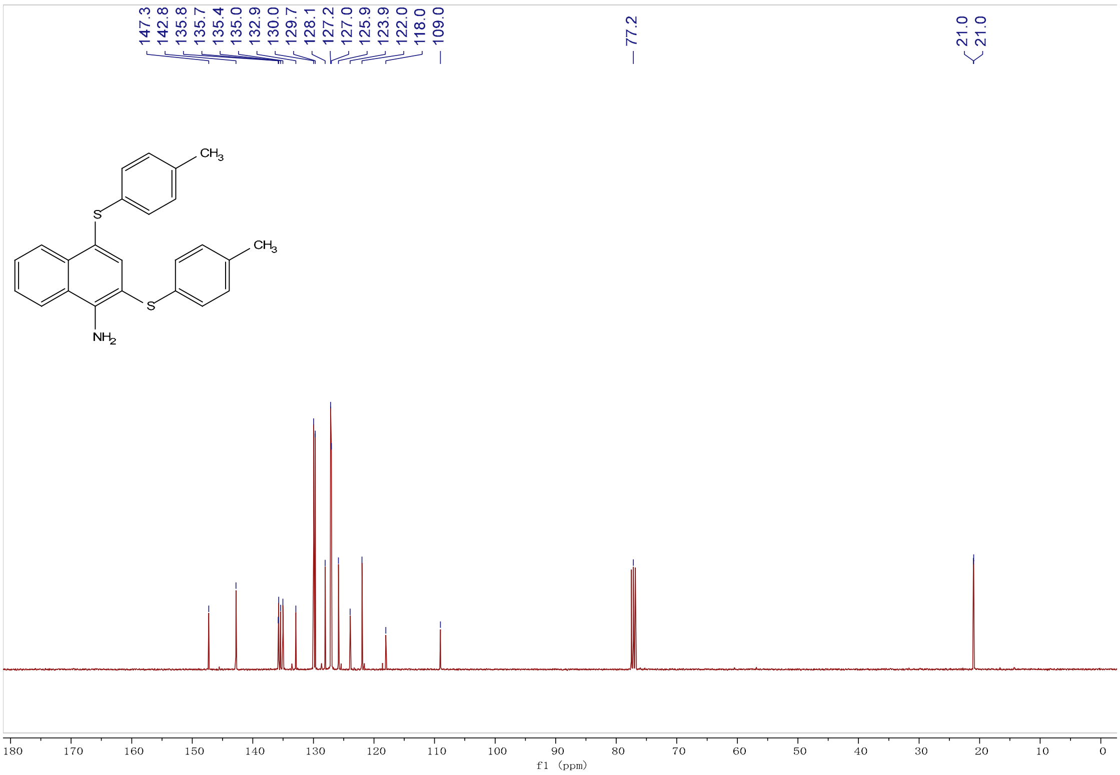

Figure S36

2,4-bis((4-chlorophenyl)thio)naphthalen-1-amine (4c)

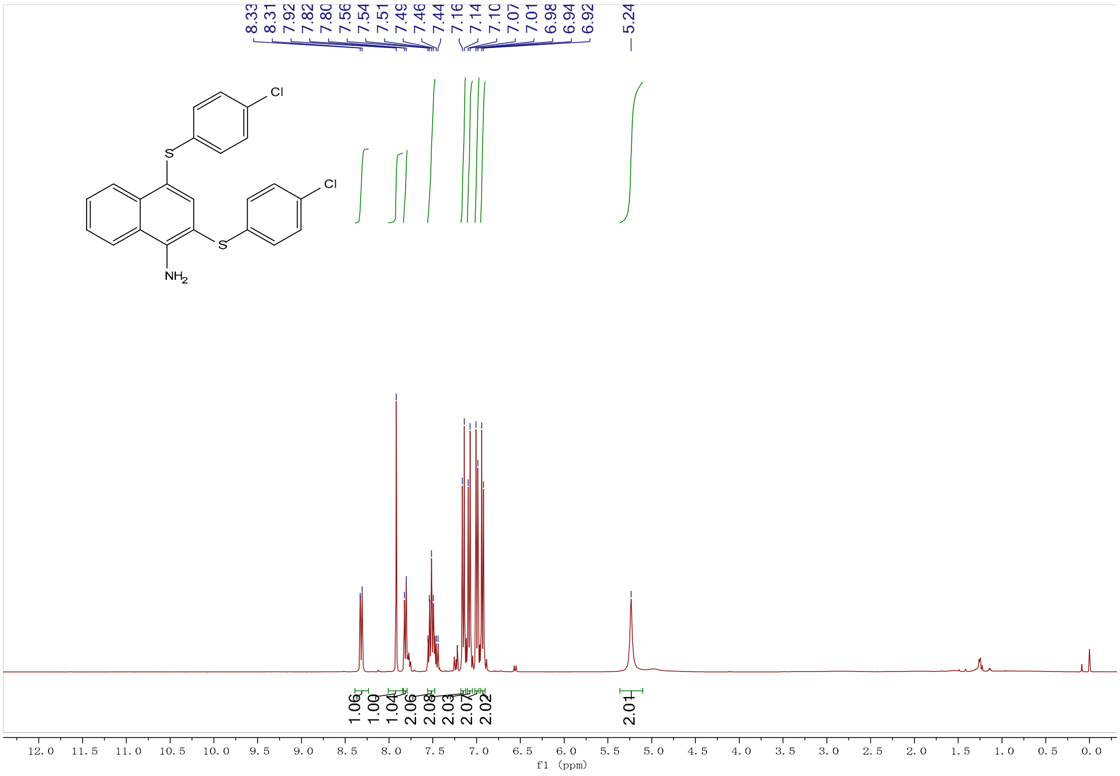

Figure S37

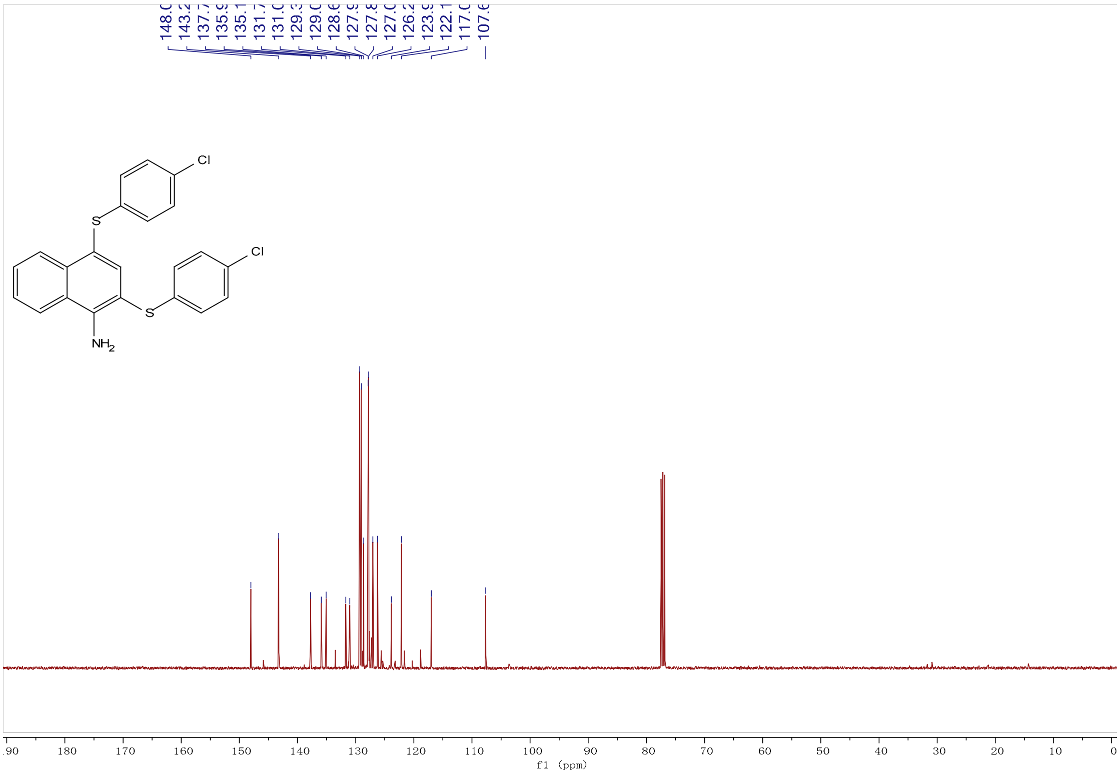

Figure S38

2,4-bis((4-bromophenyl)thio)naphthalen-1-amine (4d)

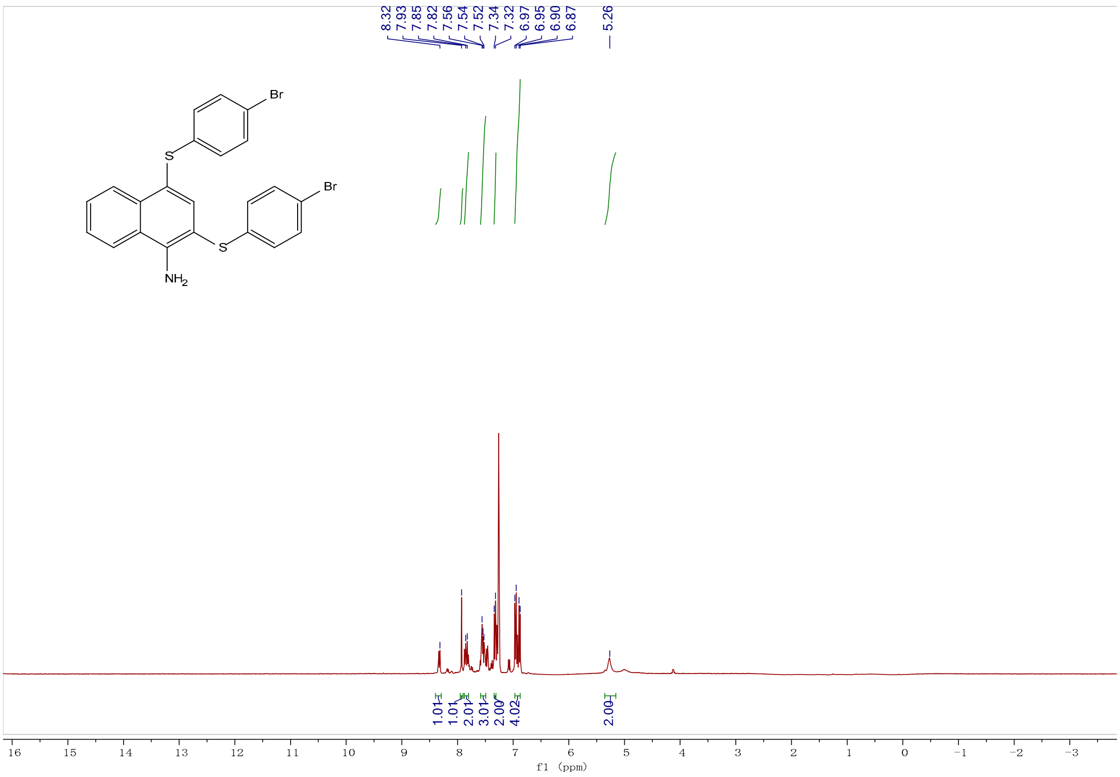

Figure S39

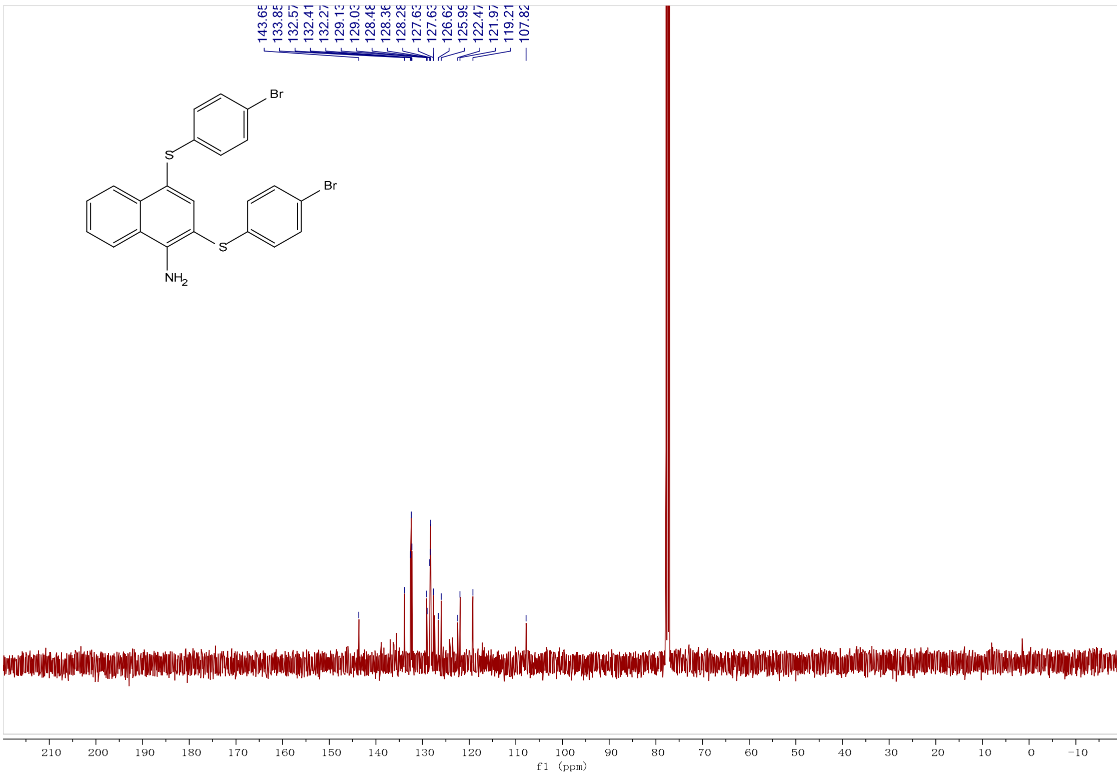

Figure S40

2,4-bis((4-fluorophenyl)thio)naphthalen-1-amine (4e)

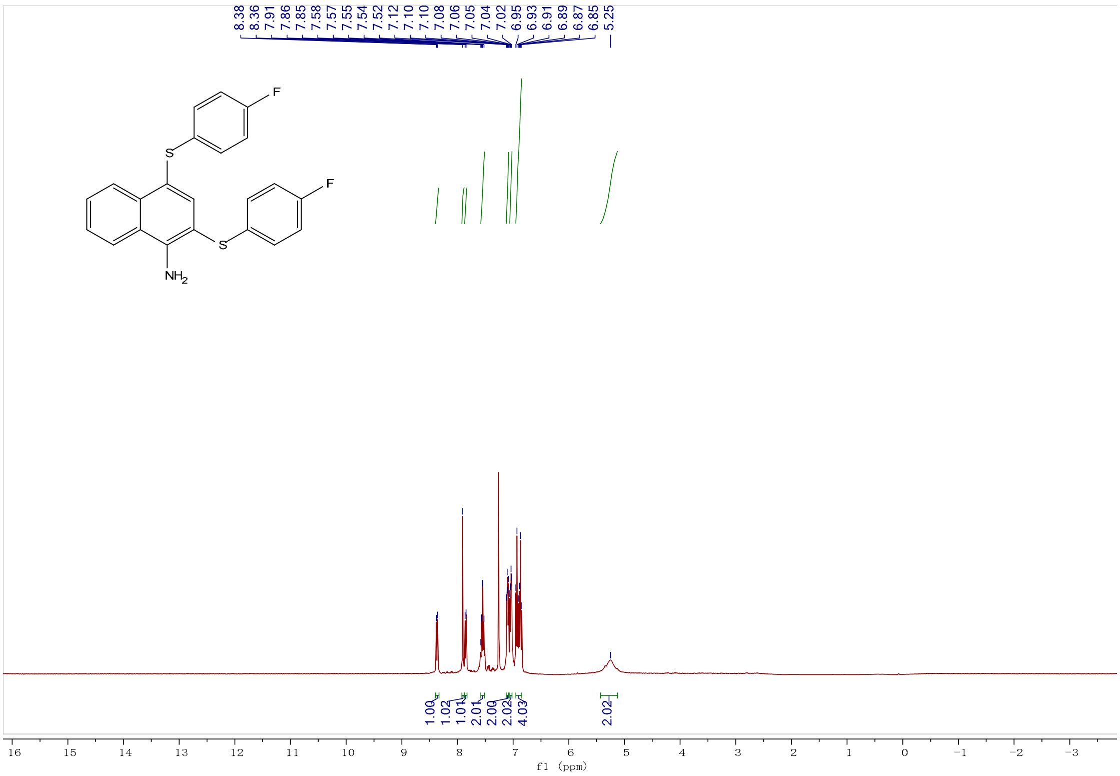

Figure S41

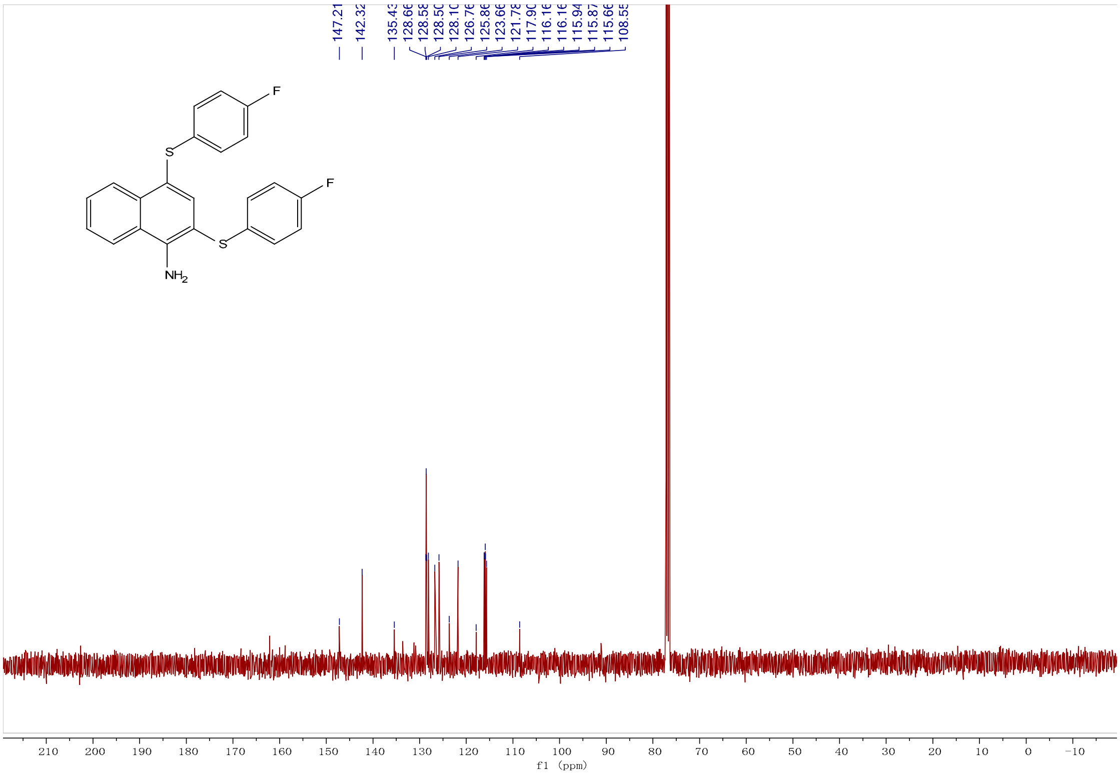

Figure S42

2,4-bis((4-methoxyphenyl)thio)naphthalen-1-amine (4f)

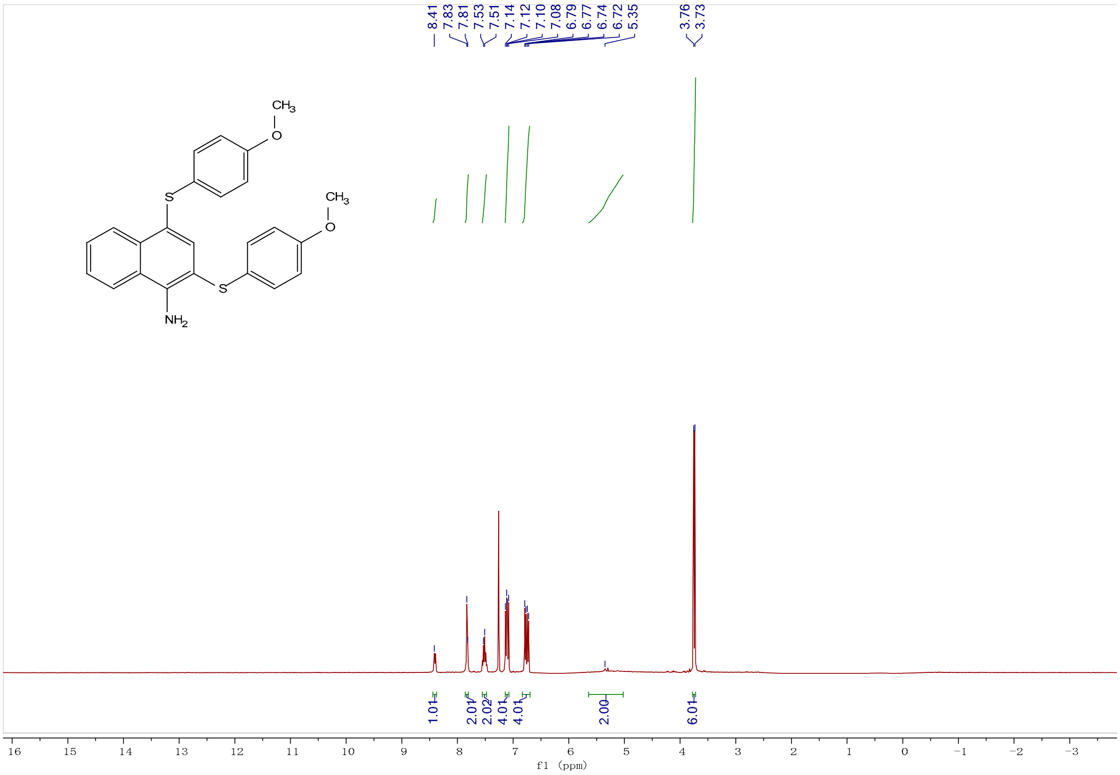

Figure S43

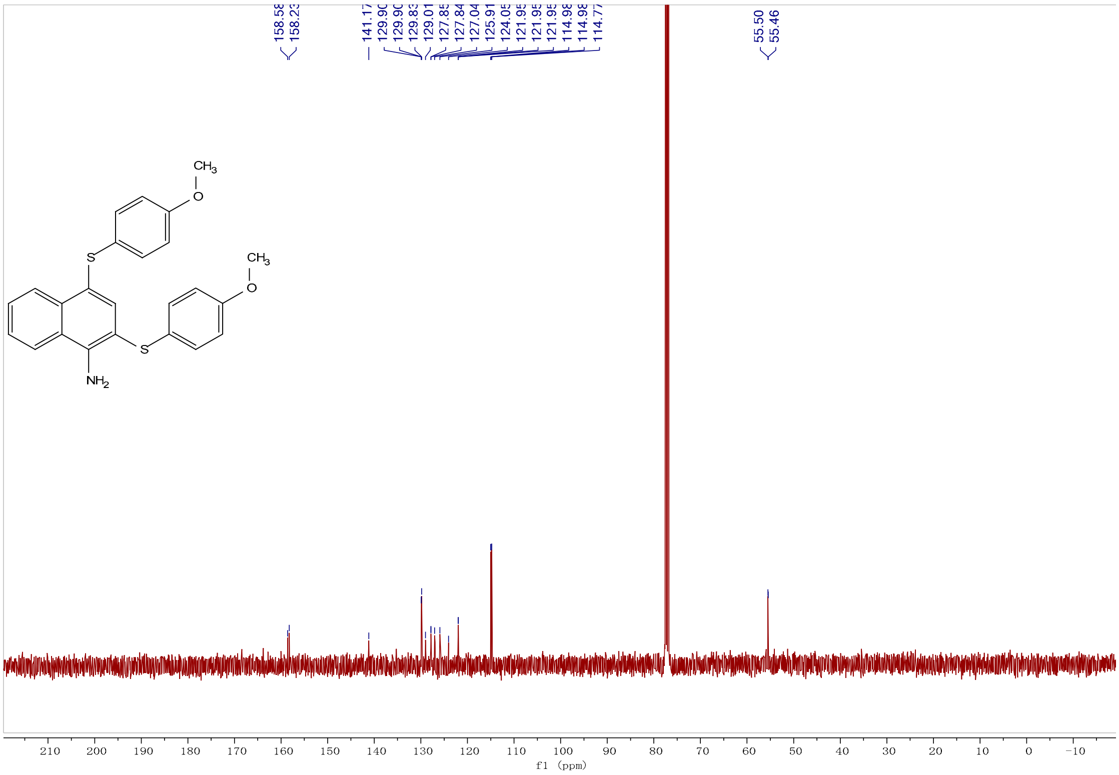

Figure S44

2,4-bis((4-(tert-butyl)phenyl)thio)naphthalen-1-amine (4g)

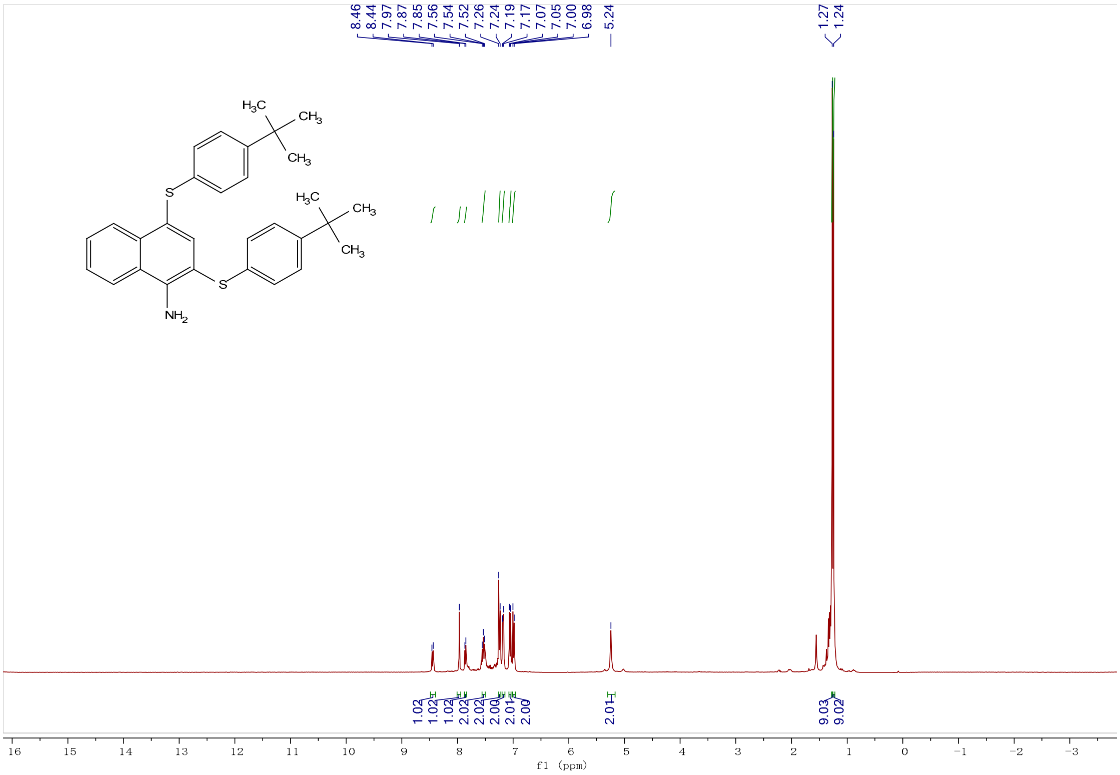

Figure S45

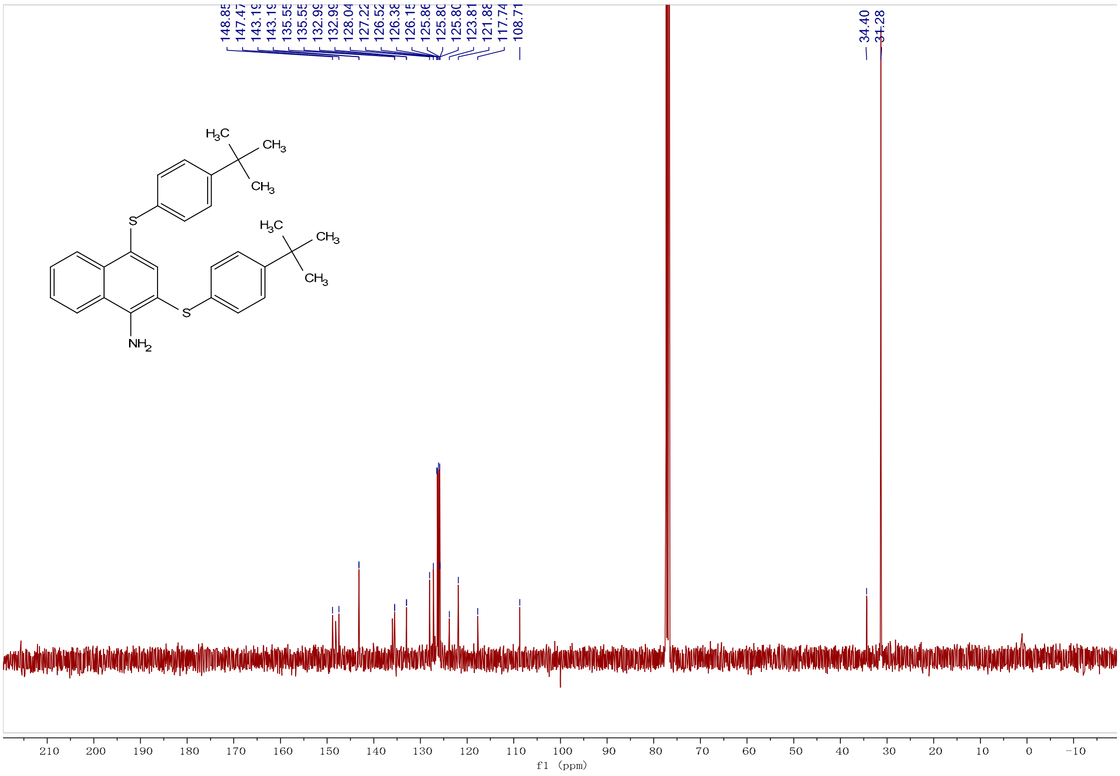

Figure S46

2,4-bis(o-tolylthio)naphthalen-1-amine (4h)

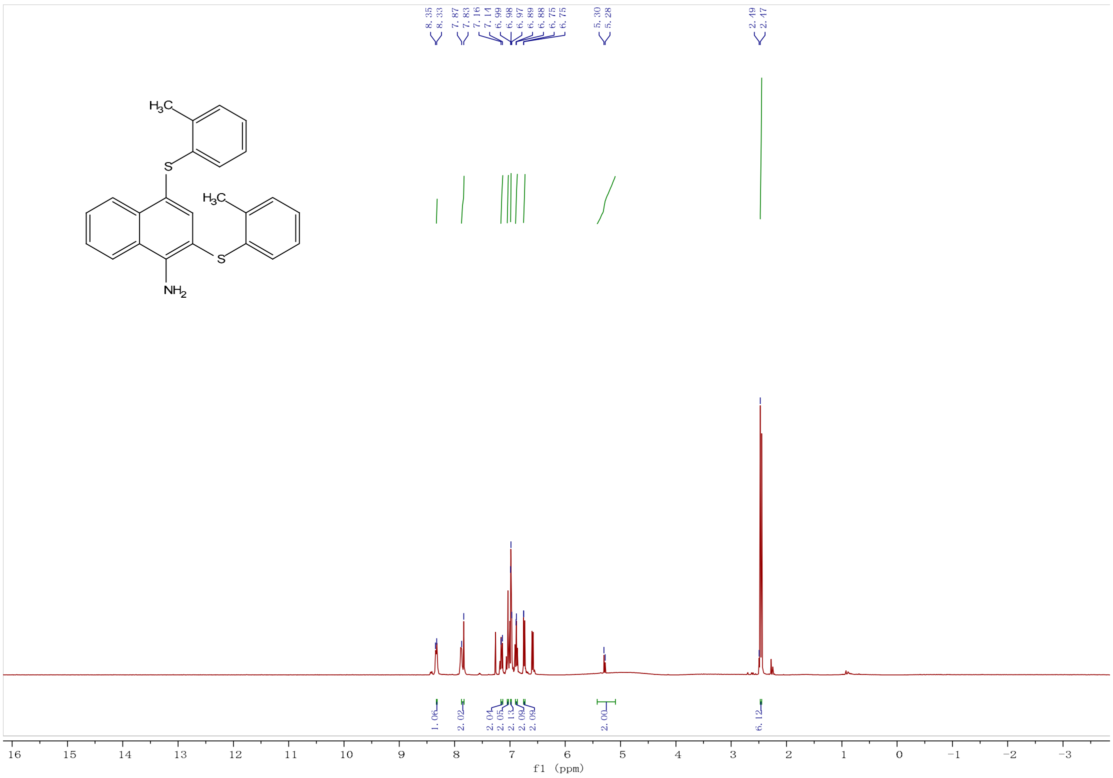

Figure S47

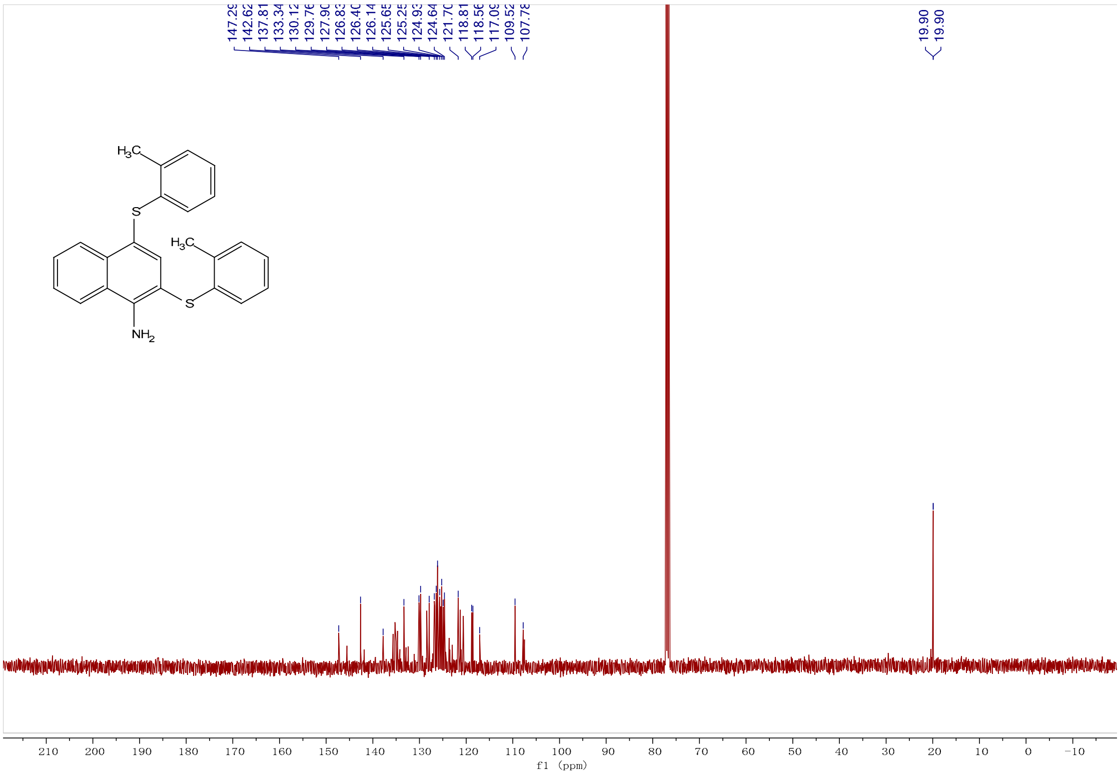

Figure S48

2,4-bis((2-fluorophenyl)thio)naphthalen-1-amine (4i)

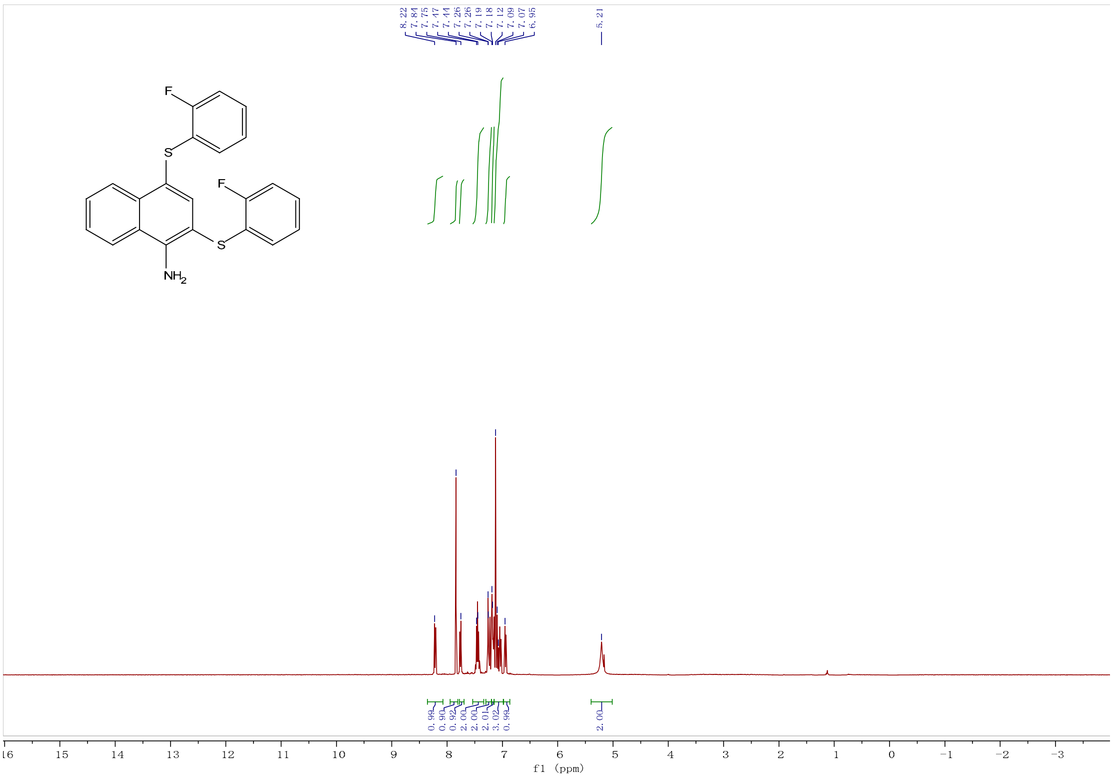

Figure S49

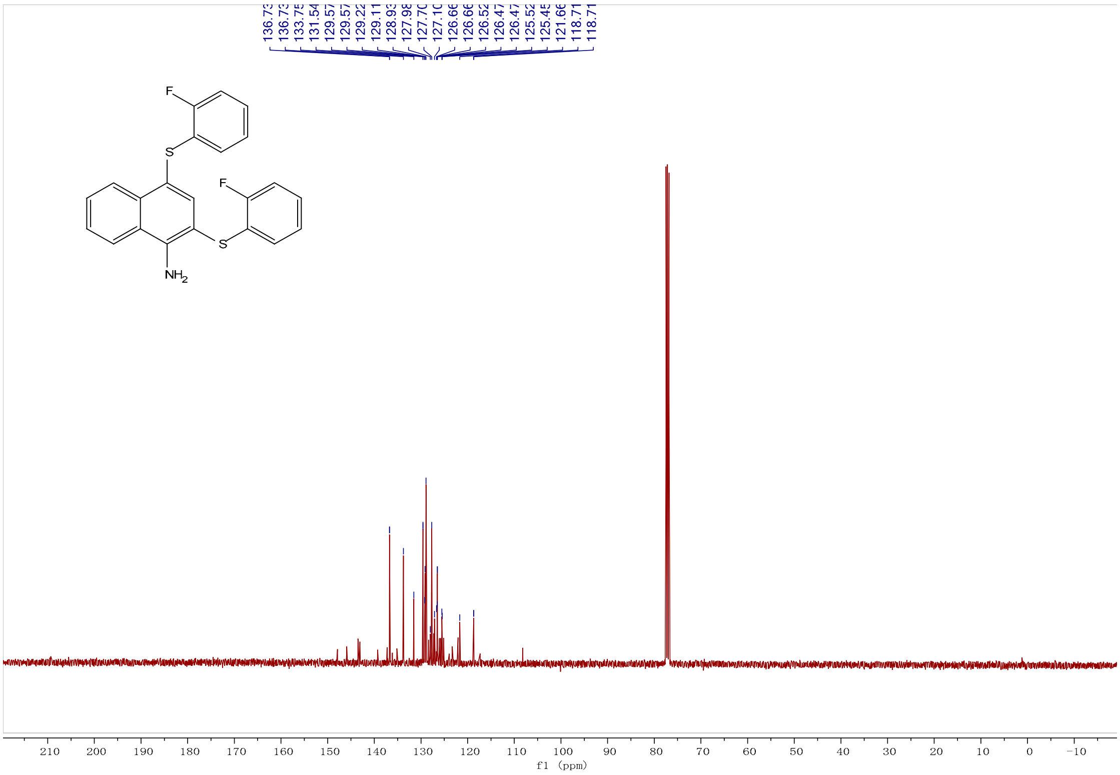

Figure S50

2,4-bis(thiophen-2-ylthio)naphthalen-1-amine (4j)

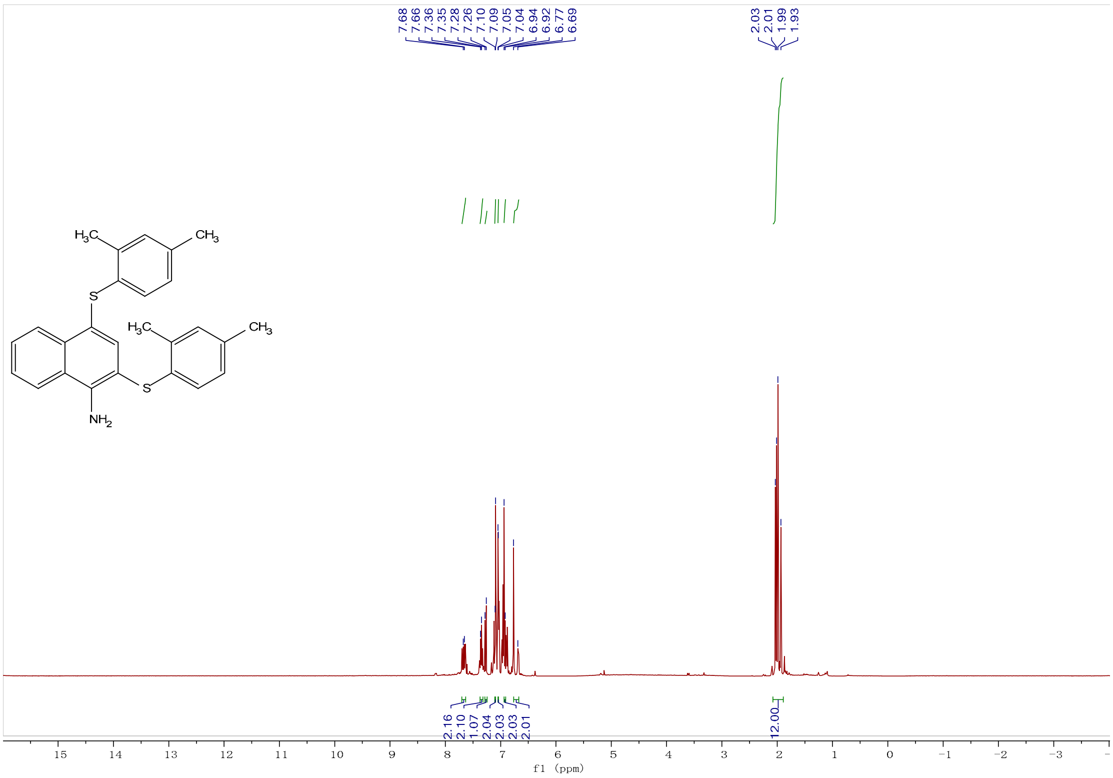

Figure S51

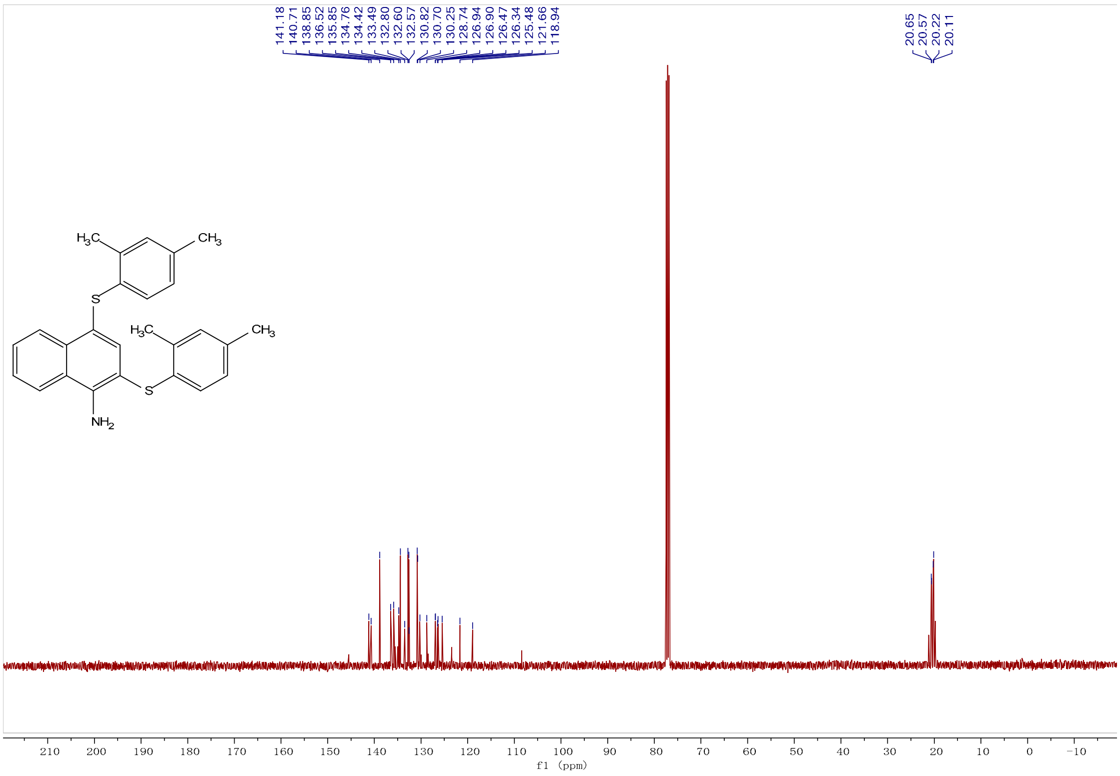

Figure S52

4-((3-bromophenyl)thio)naphthalen-1-amine (4k)

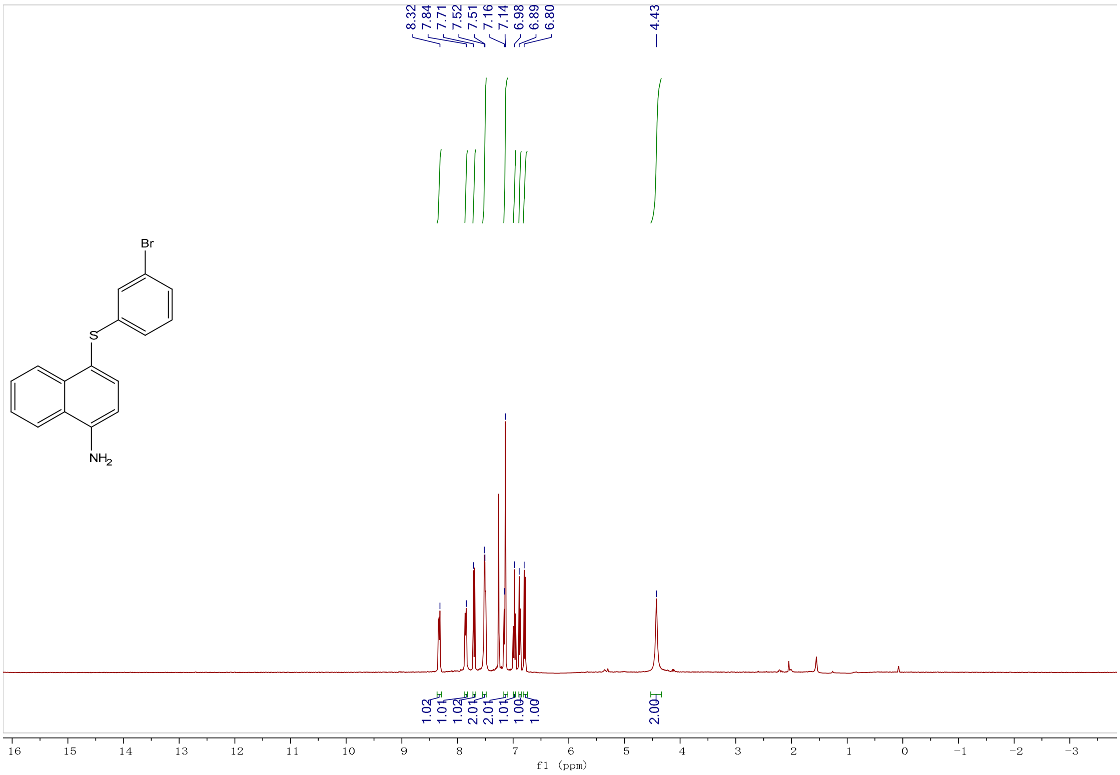

Figure S53

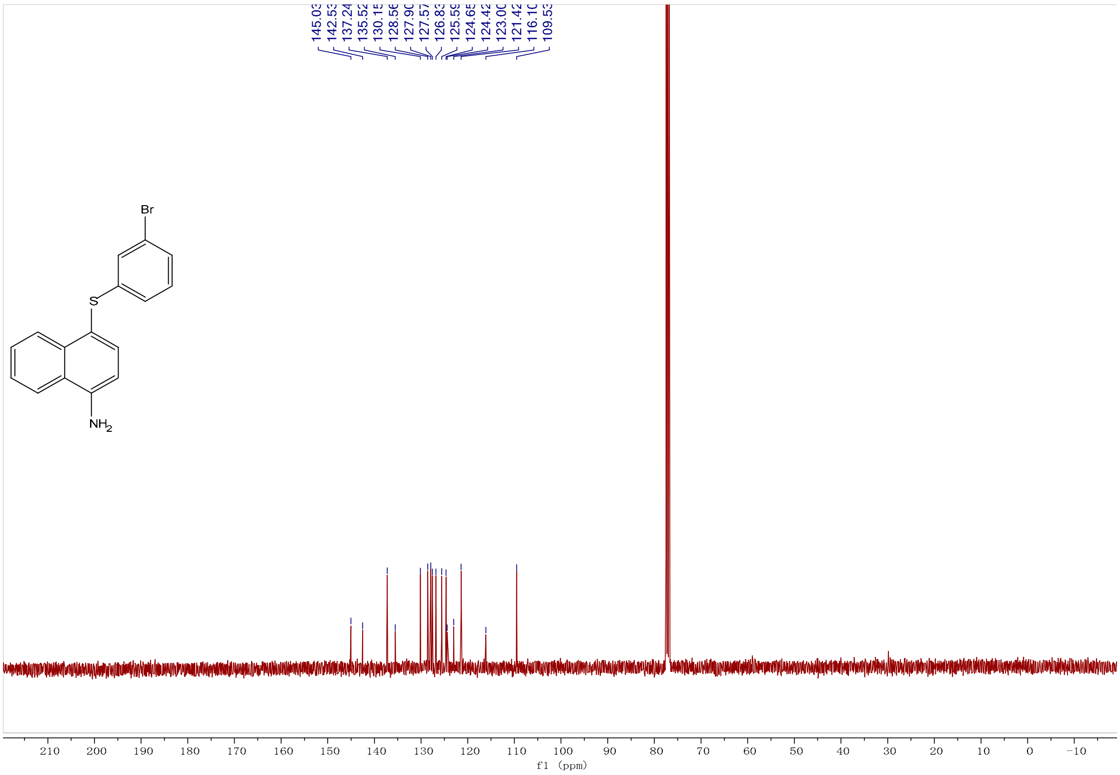

Figure S54

[4-((3-(trifluoromethyl)phenyl)thio)naphthalen-1-amine] [4l]

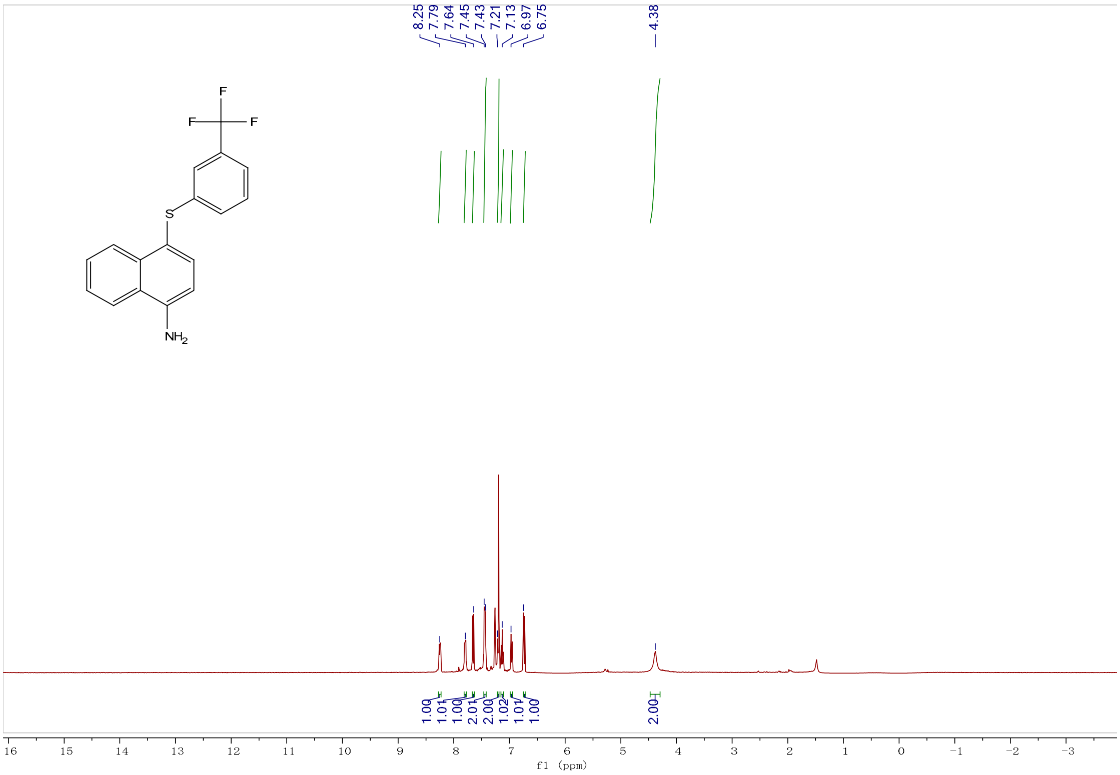

Figure S55

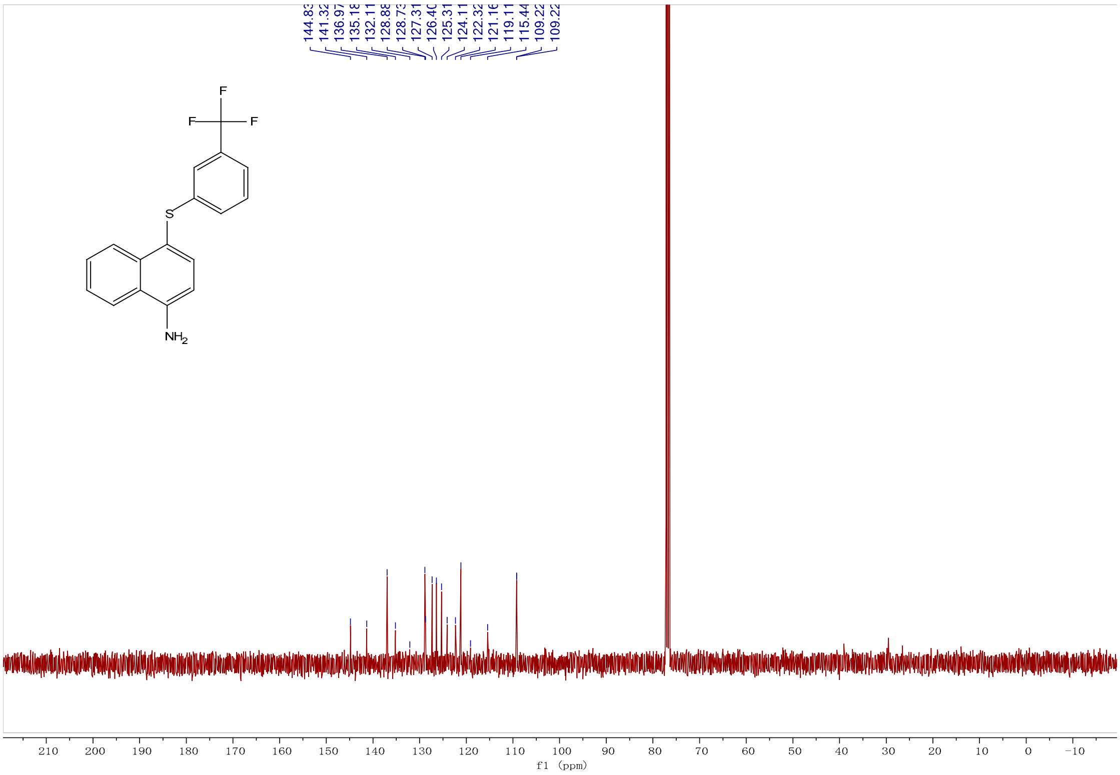

Figure S56

1,3-bis(phenylsulfinyl)naphthalen-2-amine (3aa)

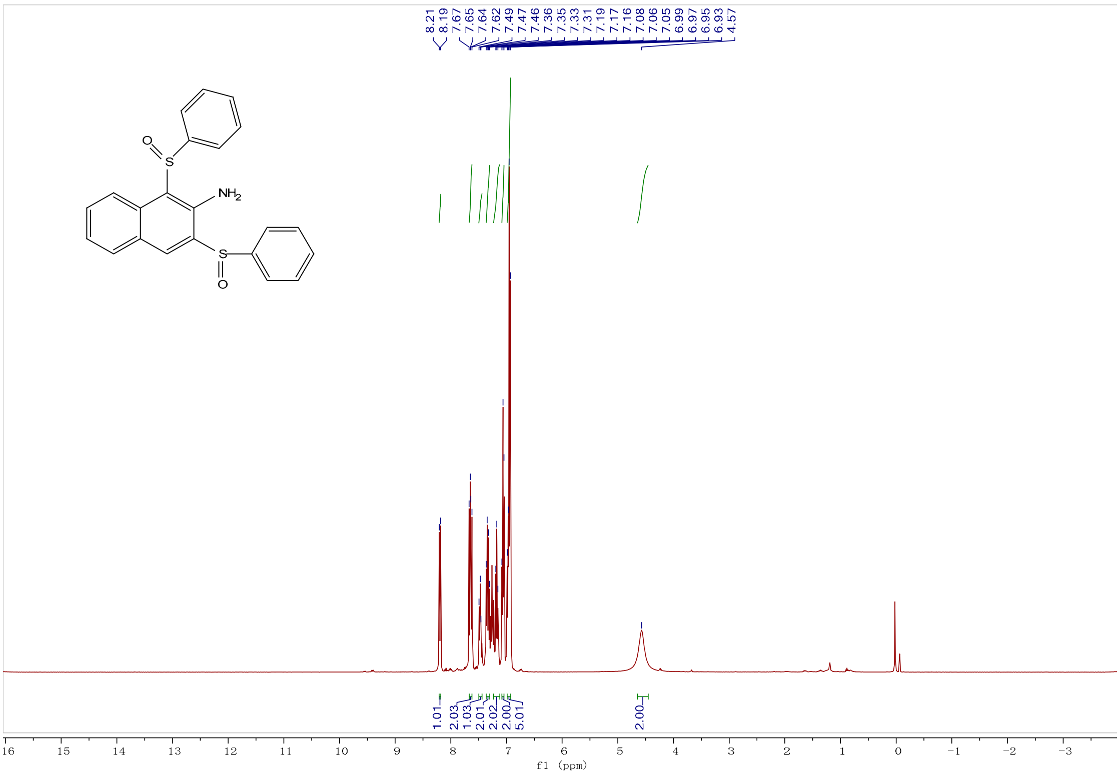

Figure S57

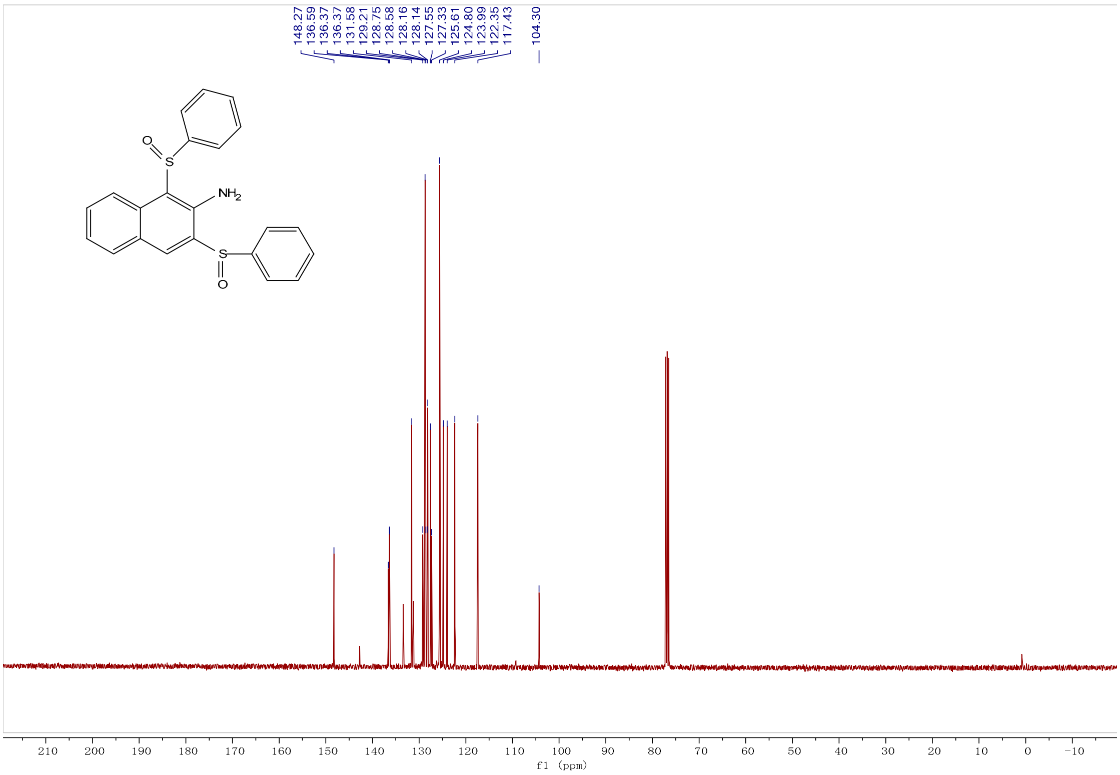

Figure S58
